# Changes in Potential Suitable Habitats, Species Turnover, and Conservation Gaps of Wild Plants in the Tarim Basin, China

**DOI:** 10.1002/ece3.74393

**Published:** 2026-09-23

**Authors:** Qiong Chen, Ziwei Dou, Chunxia Wei, Lijun Zhu, Jie Wang, Bei Li, Juntuan Zhai, Zhijun Li

**Affiliations:** ^1^ Xinjiang Production & Construction Corps Key Laboratory of Protection and Utilization of Biological Resources in Tarim Basin Alar China; ^2^ College of Life Science and Technology Tarim University, Research Center of Populus Euphratica Alar China; ^3^ Administration of the Xinjiang Tarim Populus Euphratica National Nature Reserve Korla China

**Keywords:** climate change, conservation gaps, potential species richness, species turnover, Tarim Basin, wild plants

## Abstract

Climate change is reshaping potential suitable habitats, species richness, and plant communities in arid regions. However, multi‐species assessments of distributional responses, species turnover, and conservation gaps for wild plants remain limited. Using distribution records of 608 wild plant species and 12 environmental variables, we employed the biomod2 ensemble species distribution model to predict potential suitable habitats under current conditions and three future climate scenarios for the 2050s and 2070s. Based on these predictions, we analyzed potential species richness, species turnover, conservation gaps, and habitat loss risks. The results demonstrated that the model possessed excellent predictive performance. Potential suitable habitats were mainly constrained by moisture and thermal conditions and were also influenced by human activities. Current potential species richness exhibited a pattern of higher richness in mountain and foothill transition zones and lower richness in the basin interior. Under future scenarios, areas of high potential richness are projected to continue shrinking and become increasingly fragmented, and the centroid of potential richness is projected to shift northeastward. As emission intensity increases and time advances, potential suitable habitats for most species are expected to contract and habitat loss risks to increase, with herbaceous plants being more sensitive than woody plants. Further examination of 10 representative species of conservation or ecological relevance showed deteriorating habitat‐loss risk from the 2050s to the 2070s, including several nationally or regionally protected wild plants. Hotspots of species turnover are concentrated in the central‐western basin, the northern foothill zone, and the oasis‐desert ecotone. The spatial overlap between conservation priority areas and existing nature reserves is only 1.94%, indicating a clear mismatch between the current reserve network and regions where future species composition is expected to change substantially. Future conservation efforts should focus on mountain‐basin transition zones, the foothill oasis‐desert ecotone, and high‐turnover conservation gaps, while optimizing spatial coverage and ecological connectivity. The findings provide a scientific basis for wild plant diversity conservation, precise management, and optimization of conservation system gaps in the Tarim Basin.

## Graphical abstract

Using ensemble species distribution models for 608 wild plant species, this study assessed future changes in potential suitable habitats, species richness, species turnover, and conservation gaps in the Tarim Basin under climate change. The results show shrinking and fragmented high‐richness areas, increasing habitat loss risk, and high species‐turnover hotspots mainly in the central‐western basin, northern foothill zones, and oasis‐desert ecotones. Only 1.94% of conservation priority areas overlap with existing nature reserves, highlighting the need for dynamic and adaptive conservation planning.

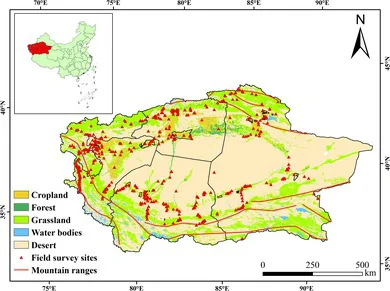

## 1 Introduction

Climate change is one of the major drivers of global biodiversity loss and ecosystem reorganization, and its impacts are expected to intensify in the coming decades (Wang et al. 2023). Rising temperatures, altered precipitation regimes, and more frequent extreme climatic events can reduce species' suitable ranges, exacerbate habitat fragmentation and loss, and alter ecological processes and community composition, thereby reshaping biodiversity patterns (Hao et al. 2024). Under high‐emission scenarios, the probability of global warming exceeding 1.5°C–2.0°C increases substantially, which may further accelerate species extinction and ecosystem reorganization (Zhou et al. 2025). Geographic distribution is a key spatial attribute of a species, shaped by long‐term interactions with its environment (Pound et al. 2020). Global climate change has already caused pronounced shifts in the geographic distributions of many species (Sun et al. 2020). Therefore, understanding the mechanisms underlying species' responses to climate change is essential for maintaining ecosystem stability and developing scientifically based conservation strategies (Chen, Shao, et al. 2025). Human activities also play an important role in shaping biodiversity patterns, particularly through disturbances such as overgrazing and human‐induced fires (Perrino et al. 2021).

Arid and semi‐arid regions are among the terrestrial ecosystems most sensitive to climate change because of strong water limitation, low ecological resilience, and high environmental variability (Lan et al. 2022). Xinjiang is an important part of the Central Asian arid region, where climate warming and hydrological changes have already affected regional vegetation dynamics and ecosystem functioning (Yao et al. 2019). The mountain–oasis–desert composite ecosystem of the Tarim Basin is characterized by pronounced topographic and moisture gradients, resulting in marked spatial differentiation in plant distributions among different ecological units. Previous studies have shown that vegetation distribution in the Tarim Basin is closely associated with regional topography, with clear differences among mountainous areas, piedmont transition zones, oasis–desert ecotones, and the basin interior (Li et al. 2023). Studies on seed plants in Xinjiang have shown that species richness is concentrated mainly in mountainous areas, whereas inland basins such as the Tarim Basin have relatively low species richness (Huang et al. 2018). Research on woody plants in Xinjiang has further demonstrated that species diversity patterns are closely related to major mountain systems and their surrounding topographic units (Zhang and Zhang 2014). Therefore, focusing on the differences in plant distribution patterns across various ecological units in the Tarim Basin helps clarify the spatial characteristics of plant diversity in extremely arid regions at the regional scale and provides a foundation for biodiversity conservation under climate change.

Hotspot and conservation gap analyses are useful to integrate species diversity, endemism, and habitat characteristics for identifying conservation priorities (Xu et al. 2017). Previous works on the topic show that the existing nature reserve network in Xinjiang inadequately covers plant diversity hotspots. Clear conservation gaps occur in the core area of the study area and in other county‐level regions, indicating a spatial mismatch between the current conservation system and plant diversity hotspots (Huang et al. 2018). This phenomenon is not limited to this region. A global‐scale study found that more than 60% of extrapolated vascular plant diversity hotspots lie outside existing protected areas, highlighting substantial gaps in the current protected‐area network (Daru 2024). At the regional scale, a recent study in the Azores identified several endemic‐richness hotspots that were either only partially encompassed by existing protected areas or remained entirely unprotected, further demonstrating spatial gaps between areas of high plant endemism and the current protected‐area network (Roxo et al. 2026). Although protected areas are intended to provide refugia for native biodiversity (Perrino et al. 2013), previous studies have often focused on specific groups such as rare, endangered, or medicinal plants (Hu, Ji, et al. 2025). However, spatial patterns of diversity often differ among taxonomic and functional groups (Yang et al. 2022). Under the combined pressures of global change and human activities, continuing habitat degradation and fragmentation make the scientific identification of conservation priority areas an urgent challenge (Yang et al. 2026). Hence, identifying priority conservation areas and assessing conservation gaps are important for optimizing regional protected area networks and improving conservation efficiency.

Species distribution is influenced by multiple factors, including bioclimatic conditions, topography, soil, hydrological processes, and human activities; these factors collectively determine habitat suitability and long‐term population viability (Zhang et al. 2025). Species distribution models (SDMs) are important tools for quantifying climatic responses and projecting changes in potential species distributions (Wang et al. 2025). Predictions from single algorithms are subject to algorithmic uncertainty, whereas ensemble modeling can improve projection robustness (Cao et al. 2024; Kang et al. 2025). Given the complex environmental gradients in the Tarim Basin, ensemble models are more applicable than single‐algorithm approaches. biomod2 is a widely used ensemble SDM framework that enhances the robustness of geographic distribution projections by integrating predictions from multiple algorithms (Zhu et al. 2025; Thuiller 2014).

Based on this context, the present study focuses on the mountain‐oasis‐desert complex system of the Tarim Basin and uses the biomod2 ensemble species distribution model to systematically assess potential species richness, species turnover, conservation gaps, and habitat loss risks of wild plants under climate change. The study addresses the following questions: (1) Which environmental factors contribute most to the prediction of potential suitable habitats for wild plants in the Tarim Basin and may explain the spatial variation in potential species richness patterns? (2) Under different future climate scenarios, what spatial characteristics will changes in potential species richness patterns and potential suitable habitats exhibit? (3) Will future species turnover hotspots spatially align with existing nature reserves, and which areas should be designated as conservation priority zones and critical conservation gaps? The findings provide a scientific basis for plant diversity conservation, nature reserve optimization, and adaptive management in extremely arid regions, and offer guidance for conservation response strategies for vulnerable ecosystems under global climate change.

## 2 Study Area and Materials

### 2.1 Study Area

**FIGURE 1:**
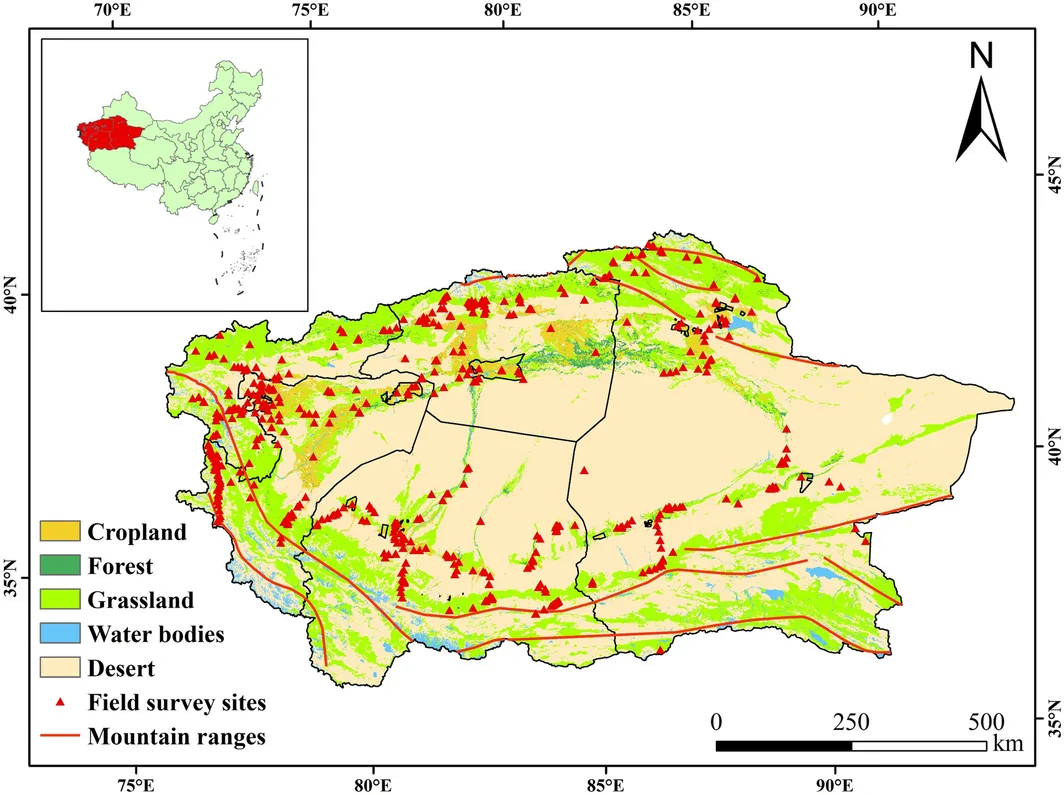
Location and overview of the study area in the Tarim Basin, China. This map is based on a map [GS (2024) 0650] downloaded from the National Geographic Information Public Service Platform and used in accordance with its terms of use. The base map has not been modified.

The Tarim Basin is located in southern Xinjiang, China (34°20′–43°39′ N, 71°39′–93°45′ E), and is the largest inland basin in China. It is surrounded by major mountain systems, including the southern slopes of the Tianshan Mountains, the northern foothills of the Kunlun Mountains, and the Altun Mountains, while the Taklamakan Desert occupies most of the basin interior, forming a typical enclosed inland‐basin landscape (Figure 1). From the basin margins toward the interior, the major landscape units comprise mountainous areas, piedmont alluvial fans, river corridors, oasis–desert ecotones, and interior desert areas.

The region has a temperate continental arid climate characterized by scarce precipitation and strong evaporation. In the upper reaches of the Tarim River, the mean annual temperature is approximately 10.6°C–11.5°C, annual precipitation ranges from 50 to 70 mm, and annual pan evaporation exceeds 2100 mm (Zeng et al. 2020). Climatic conditions are even drier in the Taklimakan Desert, where annual precipitation is less than 25 mm and potential evapotranspiration is approximately 1000 mm (Wen et al. 2024). Regional water availability mainly depends on mountain precipitation and snowmelt, river runoff, and groundwater recharge.

Published field surveys have documented a range of characteristic desert‐riparian and oasis–desert transition communities in the Tarim Basin, including communities dominated by Populus euphratica, Tamarix spp., *Phragmites australis* , *Halocnemum strobilaceum* , Halostachys caspica, and Alhagi sparsifolia. To provide ecological context for the modeled species, 10 representative plant communities previously reported from the Tarim Basin, together with their major associated species and typical habitats, were compiled from published field‐based vegetation studies and were not newly classified in the present study (Zeng et al. 2020; Zhu et al. 2024; Huang et al. 2018; Huang and Wang 2018) (Table 1). These communities were compiled from published field‐based vegetation studies and were not newly classified in the present study.

**TABLE 1:** Representative plant communities, major associated species, and typical habitats and distributions reported from the Tarim Basin.

Representative plant community	Major associated species	Focal deteriorating species in this study	Typical habitat and distribution
*Populus euphratica–Tamarix ramosissima–Phragmites australis* association group	*Populus pruinosa*, *Alhagi sparsifolia*, *Lycium ruthenicum*, *Glycyrrhiza inflata*, *Karelinia caspia*, and others	*P. euphratica*, *T. ramosissima* , *P. australis* , *P. pruinosa* , *Alhagi camelorum* , *L. ruthenicum*, *G. inflata*	Desert riparian habitat in the Tarim Basin under extremely arid conditions
*Populus euphratica–Tamarix* spp. riparian community (TWINSPAN Class 1)	*Lycium ruthenicum*, *Halostachys caspica*, *Halimodendron halodendron* , *Alhagi sparsifolia*, *Karelinia caspia*, *Halocnemum strobilaceum* , and others	*P. euphratica*, *T. ramosissima* , *L. ruthenicum*, *A. camelorum*	Upper Tarim River; near the river channel toward the oasis
*Populus* spp.–*Tamarix* spp. riparian community (TWINSPAN Class 2)	*Alhagi sparsifolia*, *Karelinia caspia*, *Calamagrostis pseudophragmites*, *Poacynum hendersonii*, *Phragmites australis* , and others	*P. euphratica*, *P. pruinosa* , *T. ramosissima* , *A. camelorum* , *P. australis*	Upper Tarim River; near the river channel toward the desert
*Populus* spp.– *Tamarix ramosissima* riparian community (TWINSPAN Class 3)	*Alhagi sparsifolia*, *Glycyrrhiza inflata*, *Apocynum venetum*	*P. euphratica*, *P. pruinosa* , *T. ramosissima* , *A. camelorum* , *G. inflata*	Upper Tarim River; approximately 5 km from the river channel
*Tamarix* spp.–*Haloxylon ammodendron* community (TWINSPAN Class 4)	*Halogeton arachnoideus*, *Salsola ruthenica*	*T. ramosissima*	Upper Tarim River; dry desert‐side habitat approximately 23 km from the river channel
*Tamarix ramosissima* ‐dominated community	*Halocnemum strobilaceum* , *Halostachys caspica*, *Phragmites australis* , *Alhagi sparsifolia*	*T. ramosissima* , *P. australis* , *A. camelorum*	Oasis–desert transition zone on the northern margin of the Tarim Basin; sandy soil
*Halocnemum strobilaceum* ‐dominated community	*Halostachys caspica*, *Phragmites australis* , *Tamarix ramosissima*	*P. australis* , *T. ramosissima*	Oasis–desert transition zone on the northern margin of the Tarim Basin; sandy loam
*Halostachys caspica*‐dominated community	*Halocnemum strobilaceum* , *Tamarix ramosissima* , *Phragmites australis*	*T. ramosissima* , *P. australis*	Oasis–desert transition zone on the northern margin of the Tarim Basin; sandy loam
*Phragmites australis* ‐dominated community	*Halocnemum strobilaceum* , *Halostachys caspica*, *Alhagi sparsifolia*, *Karelinia caspia*	*P. australis* , *A. camelorum*	Oasis–desert transition zone on the northern margin of the Tarim Basin; sandy loam
*Alhagi sparsifolia*‐dominated community	*Halocnemum strobilaceum* , *Phragmites australis* , *Halostachys caspica*	*A. camelorum* , *P. australis*	Oasis–desert transition zone on the northern margin of the Tarim Basin; sandy loam

*Note:* The listed communities were compiled from published field‐based vegetation studies and were not newly classified in the present study. “Focal deteriorating species” refers to the representative species listed in Table 2 that showed increasing habitat‐loss risk from the 2050s to the 2070s. Major associated species represent selected characteristic species reported in the original studies rather than complete species inventories.

### 2.2 Acquisition and Screening of Species Distribution Data

Species distribution data were compiled from field surveys, herbarium specimens, and public databases. Field surveys used a nested plot design. A total of 438 main plots (20 m × 20 m) were established in the study area. Within each main plot, three woody plant subplots (5 m × 10 m) and three herbaceous plant subplots (1 m × 10 m) were nested. Species composition, geographic coordinates, and related information were recorded, and voucher specimens were collected. Herbarium records were obtained from the specimen collections of Shihezi University, the Xinjiang Uygur Autonomous Region Institute of Materia Medica, and other institutions. Public records were mainly obtained from the Chinese Virtual Herbarium (https://www.cvh.ac.cn/) and the Global Biodiversity Information Facility (https://www.gbif.org/). The initial dataset contained 55,125 raw occurrence records representing 2110 species. A complete inventory of the 2110 initially compiled species, including their family, genus, life form, and inclusion status in the final modeling dataset, was compiled to facilitate verification of the species data (Table S1).

To ensure data quality for modeling, we first used the R package “CoordinateCleaner” to remove duplicate records, nonnumeric coordinates, and invalid coordinates (Zizka et al. 2019). We then retained only species with at least 10 valid latitude‐longitude records after quality control, resulting in 23,682 valid distribution records for 1029 species. To reduce the effects of sampling bias and spatial autocorrelation on model fitting, we used the R package “spThin” to spatially thin the occurrence records of these 1029 species (Aiello‐Lammens et al. 2015). Specifically, a minimum distance threshold of 5 km was applied to each species. For pairs of records separated by less than 5 km, one record was randomly retained. The thinning procedure was repeated, and the solution that retained the largest number of records was selected as the final dataset. After spatial thinning, 650 species met the requirements for subsequent modeling. Of these, 608 species successfully completed model construction and were included in subsequent analyses; the remaining species were excluded because of insufficient effective sample sizes or because model evaluation metrics did not meet the preset accuracy thresholds. Previous surveys documented approximately 1657 wild plant taxa belonging to 81 families and 459 genera in the Tarim Basin (Li et al. 2013). The initial dataset compiled in this study contained 2110 species from 89 families and 557 genera, representing a multi‐source candidate species pool rather than a complete floristic inventory of the basin. After data screening and spatial thinning, 650 species met the modeling requirements, of which 608 were successfully modeled and retained for analysis 93.5%. The final 608 species comprised 60 families and 261 genera, including 487 herbaceous and 121 woody species. Although the 608 species do not constitute a complete representation of the flora of the Tarim Basin, their broad taxonomic and life‐form coverage provides a reasonable basis for regional‐scale multi‐species analyses.

### 2.3 Acquisition and Filtering of Environmental Data

We collected multidimensional environmental variables representing climate, topography, soil, hydrology, and human activities. Data sources and processing procedures were as follows.

1. Climate data: Nineteen bioclimatic variables for the baseline climate period (1970–2000) and future climate scenarios were obtained from WorldClim v2.1 (https://worldclim.org/) at a spatial resolution of 30 arc‐seconds. To account for differences among climate models and projection uncertainty, future climate data were derived from the Beijing Climate Center Climate System Model (BCC‐CSM2‐MR) from Coupled Model Intercomparison Project Phase 6 (CMIP6) (Zhao et al. 2024). Three Shared Socioeconomic Pathways (SSPs) were selected: SSP1‐2.6 (low radiative forcing), SSP3‐7.0 (medium radiative forcing), and SSP5‐8.5 (high radiative forcing).
2. Topographic data: Topographic data were obtained from the Geospatial Data Cloud platform (http://www.gscloud.cn/). Elevation, slope, and aspect were extracted using geographic information system (GIS) tools.
3. Soil data: Soil variables were obtained from the Harmonized World Soil Database v2.0 (HWSD v2.0; https://www.fao.org/soils‐portal/).
4. Hydrological data: Hydrological data were obtained from the Resource and Environmental Science Data Center of the Chinese Academy of Sciences (https://www.resdc.cn/). The Euclidean distance from each grid cell to the nearest river was calculated and used as a hydrological variable.
5. Human footprint (HPF): The 2009 Global Human Footprint dataset published by Venter et al. (2016) was used.

**FIGURE 2:**
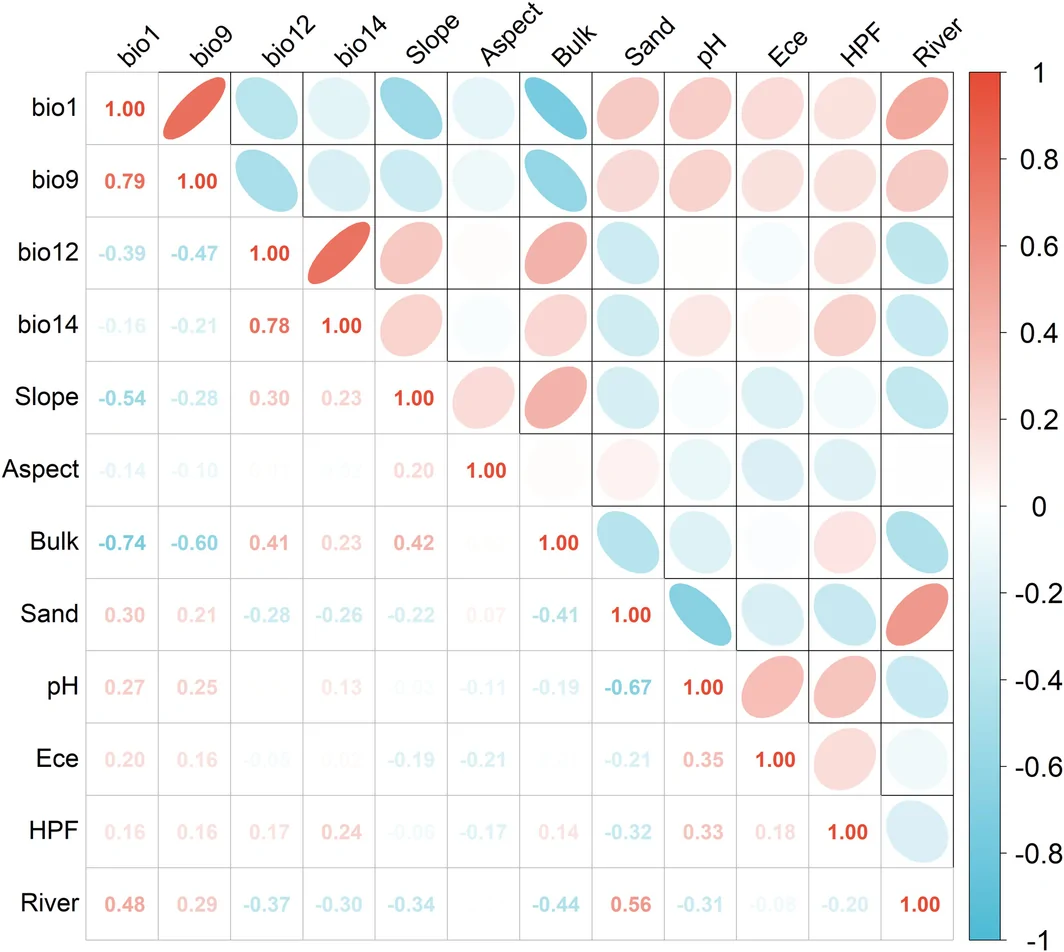
Pearson correlation analysis of environmental variables.

To ensure consistency and comparability among environmental variables, all nonclimatic variables were resampled to a spatial resolution of 30 arc‐seconds and standardized to the WGS 84 geographic coordinate reference system (EPSG:4326), ensuring that all layers had the same spatial resolution, coordinate system, and spatial extent. Species occurrence records were retained as geographic point coordinates rather than being resampled, and each occurrence point was spatially matched to the corresponding 30‐arc‐second environmental raster cell during model fitting. For future projections, only the climatic variables were updated according to the different SSP scenarios and time periods, whereas topographic, soil, hydrological, and human‐footprint variables were treated as static baseline predictors and held constant across all future projections. Because multicollinearity among environmental variables can reduce model accuracy and increase overfitting, we used the “vifcor” and “vifstep” functions in the R package “usdm” to remove highly correlated variables with absolute Pearson correlation coefficients > 0.8 and variance inflation factors (VIF) > 10 (Chen et al. 2019). After filtering, 12 mutually independent environmental variables were retained for model construction and projection (Figure 2).

### 2.4 Model Optimization and Accuracy Evaluation

#### 2.4.1 Generation of Pseudo‐Absence Points

To reduce spatial sampling bias in species distribution data, we generated pseudo‐absence points using the target‐group background approach (Phillips et al. 2009). Specifically, occurrence points of all nontarget species were used as the background pool. For each target species, a 20‐km buffer was created around its occurrence points. Occurrence points of the target species within this buffer were removed from the background pool, and background points outside the buffer were retained as candidate pseudo‐absence points (VanDerWal et al. 2009). We then randomly sampled, without replacement, the same number of pseudo‐absence points as the number of occurrence points for the target species. This procedure was repeated three times to generate three independent pseudo‐absence datasets (Čengić et al. 2020). This approach makes pseudo‐absence points and occurrence points more comparable in terms of sampling bias, allowing the model to focus more on environmental differences rather than differences in sampling intensity and reducing spatial bias associated with survey accessibility.

#### 2.4.2 Model Algorithms and Training

To combine the predictive advantages of different algorithms, we used the “biomod2” package in R 4.3.3 for species distribution modeling. From an initial set of 10 algorithms, six algorithms with relatively strong predictive performance were selected for subsequent analyses: generalized linear model (GLM), generalized additive model (GAM), generalized boosted model (GBM), flexible discriminant analysis (FDA), multivariate adaptive regression splines (MARS), and maximum entropy with Lasso regularization (MAXNET). Based on occurrence records and the three pseudo‐absence datasets, models were trained and evaluated using cross‐validation. In each run, 70% of the samples were randomly assigned to the training set and the remaining 30% to the validation set (Thuiller et al. 2009). This process was repeated 20 times to reduce uncertainty caused by random data partitioning. Combining three pseudo‐absence datasets, six algorithms, and 20 repetitions, 360 base models were constructed for each species. Model accuracy was evaluated using the area under the receiver operating characteristic curve (AUC) and the true skill statistic (TSS); values closer to 1 indicate better predictive performance.

#### 2.4.3 Building an Integrated Model

To improve projection robustness, base models with TSS ≤ 0.6 or AUC ≤ 0.8 were excluded. For each algorithm, the model with the highest TSS was selected, and ensemble predictions were generated using a TSS‐weighted mean. To avoid excessive extrapolation, we used the “clampingmask” function in biomod2 to identify areas where environmental conditions differed substantially from those in the observed distribution area of each species, and predictions in these areas were masked (Biancolini et al. 2024). We extracted the relative contribution values of environmental variables from each species model and aggregated the results for the 608 successfully modeled species to calculate the comprehensive relative contribution of each environmental variable, which was used to assess their overall importance in predicting potential suitable habitats across multiple species. Potential species richness was derived by overlaying the binary suitability grids for the 608 species and was classified into four categories—areas with no predicted suitable species, areas with low potential richness, areas with moderate potential richness, and areas with high potential richness—using the Jenks natural breaks method. To ensure comparability across different scenarios, the classification thresholds from the current scenario were adopted as a uniform standard and applied to all future scenarios. Based on the potential species richness rasters described above, spatial shifts in the overall richness pattern under future climate change were quantified using the Mean Center tool in ArcGIS 10.8. The spatial centroid of potential species richness was calculated for the current period and for the 2050s and 2070s under SSP1‐2.6, SSP3‐7.0, and SSP5‐8.5, with the potential species richness value of each raster cell used as the weight. The centroid under current climate conditions was used as a common reference, and the spatial displacement of each future centroid relative to the current centroid was calculated. Migration distance and direction were determined from changes in centroid location, allowing comparison of spatial shifts in the overall potential species richness pattern among future scenarios and time periods.

#### 2.4.4 Dynamic Changes in Suitable Habitat and Species Risk Assessment Based on Habitat Loss

To quantify changes in potential suitable habitat for each species under future climate scenarios, we used species‐specific binary potential suitable habitat grids to calculate the percentage change in potential suitable habitat (CSH) and the percentage loss of habitat (LSH) using the “terra” R package (Sun et al. 2025). For each species, we calculated the number of current potential suitable habitat pixels (A_current_), the number of future potential suitable habitat pixels (A_future_), and the number of overlapping pixels (A_overlap_). Because all rasters had the same resolution and spatial extent, pixel counts were directly compared. The two metrics were calculated as follows:

(1) $$\mathrm{CSH}\left(\%\right)=\frac{A_{\text{future}}-A_{\text{current}}}{A_{\text{current}}}\times 100$$

(2) $$\mathrm{LSH}\left(\%\right)=100-\frac{A_{\text{overlap}}}{A_{\text{current}}}\times 100$$

CSH quantifies the expansion or contraction of a species' potential suitable habitat; positive values indicate expansion, whereas negative values indicate contraction. LSH represents the proportion of current potential suitable habitat that will be lost under future climate scenarios; higher values indicate more severe habitat loss. We used LSH ≥ 30% as the threshold for classifying species as being at high risk (Ye et al. 2025). LSH ≥ 80% was used as the threshold for severe habitat loss. Species were ranked according to the number of times they reached the severe‐loss threshold across the three climate scenarios. Species reaching the threshold under three, two, one, and zero scenarios were assigned scores of 1, 2, 3, and 4, respectively. By comparing scores between the 2050s and 2070s, risk status was classified as deteriorated, stable, or improved.

#### 2.4.5 Identification of Species Turnover Hotspots and Conservation Gap Analysis

Based on the current and future binary grids of potential suitable habitats for all species in the study area, we calculated species turnover on a pixel‐by‐pixel basis using R (Ye et al. 2025). For each pixel, we calculated current potential species richness (SR), the number of predicted suitable species lost (L), and the number of predicted suitable species gained (G). SR represents the number of species predicted to have potential suitable habitat in that pixel under current climate conditions; L represents the number of species currently predicted as suitable but no longer predicted as suitable in the future; G represents the number of species not currently predicted as suitable but newly predicted as suitable in the future. In a binary raster, a pixel value of 1 indicates that the species is predicted to have potential suitable habitat in that pixel, while a value of 0 indicates no predicted suitable habitat. Species turnover was calculated as:

(3) $$\text{Turnover}\left(\%\right)=\frac{\text{L}+\text{G}}{\mathrm{SR}+\text{G}}\times 100$$

The Getis‐Ord Gi* statistic was used to identify spatial clustering patterns of species turnover (Ord and Getis 1995). Hotspot maps from the six future climate scenarios were overlaid, and areas identified as hotspots under any scenario were extracted as the comprehensive hotspot area. This area was treated as the conservation priority area under future climate change. The comprehensive hotspot area was then spatially overlaid with nature reserves in the study area to identify protected hotspots and conservation gaps. To compare spatial patterns of species turnover among climate scenarios, the SSP1‐2.6 scenario for the 2050s was used as the baseline. Classification thresholds were determined using the Jenks natural breaks method and then applied uniformly to all other scenarios to ensure comparability. Nature reserve data were obtained from the Resource and Environment Science and Data Center (https://www.resdc.cn/Default.aspx), and the boundaries of nature reserves within the Tarim Basin were extracted according to the extent of the study area. According to the administrative‐level information provided in the source dataset, the reserves included in the analysis comprised national‐level, Xinjiang Uygur Autonomous Region‐level (provincial‐level), and municipal‐level nature reserves.

## 3 Results

### 3.1 Model Performance Evaluation

**FIGURE 3:**
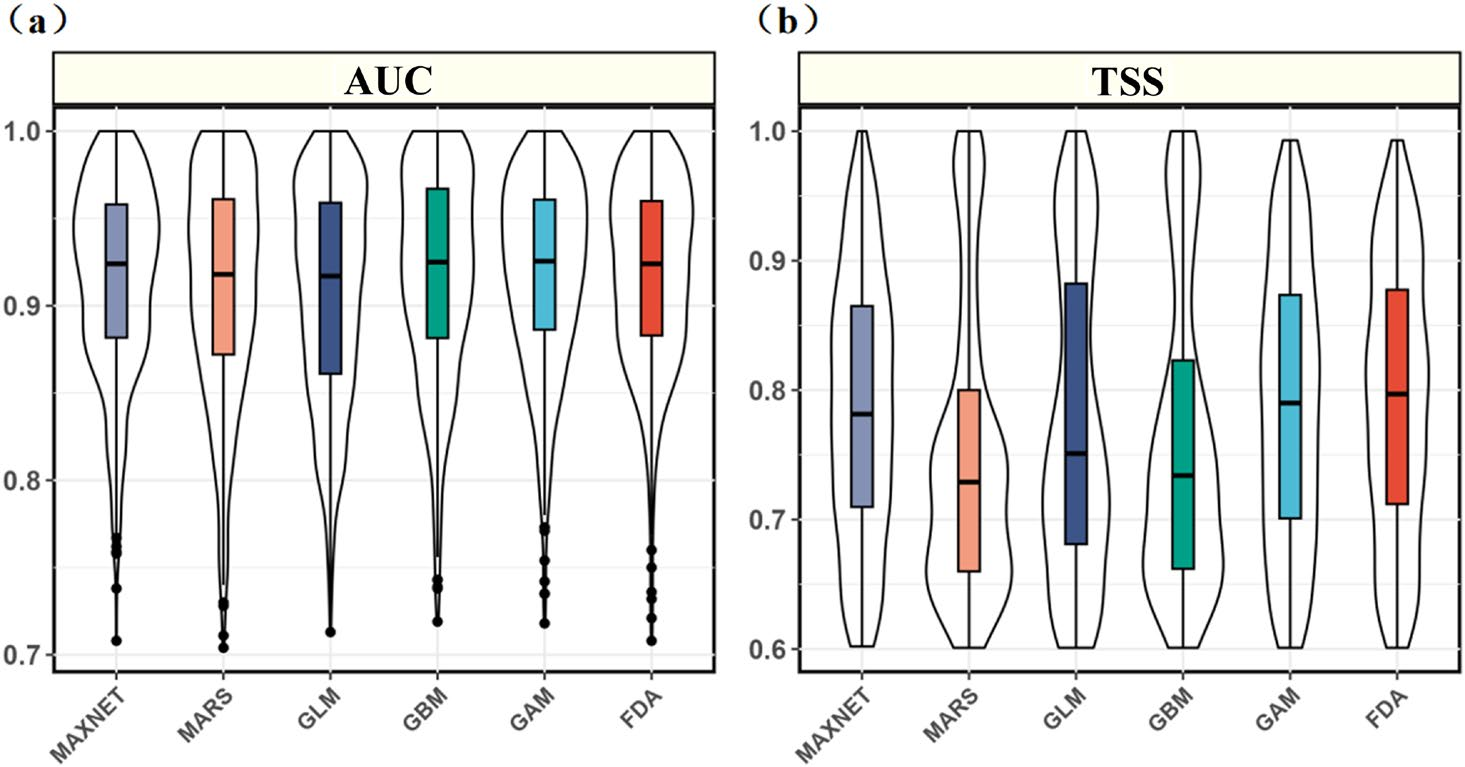
Predictive performance of different modeling algorithms evaluated by AUC and TSS values.

Model evaluation results showed that all models had high overall predictive accuracy (Figure 3). AUC values were generally high and varied little among algorithms, indicating strong discriminatory ability. TSS values were greater than 0.70 for all models, suggesting that the projections were reliable and stable. In summary, the integrated model developed in this study provides a reliable basis for predicting potential suitable habitats for each species and further supports the identification of potential species richness, species turnover, and conservation gaps based on multi‐species binary classification results.

### 3.2 Current and Future Potential Species Richness Patterns

**FIGURE 4:**
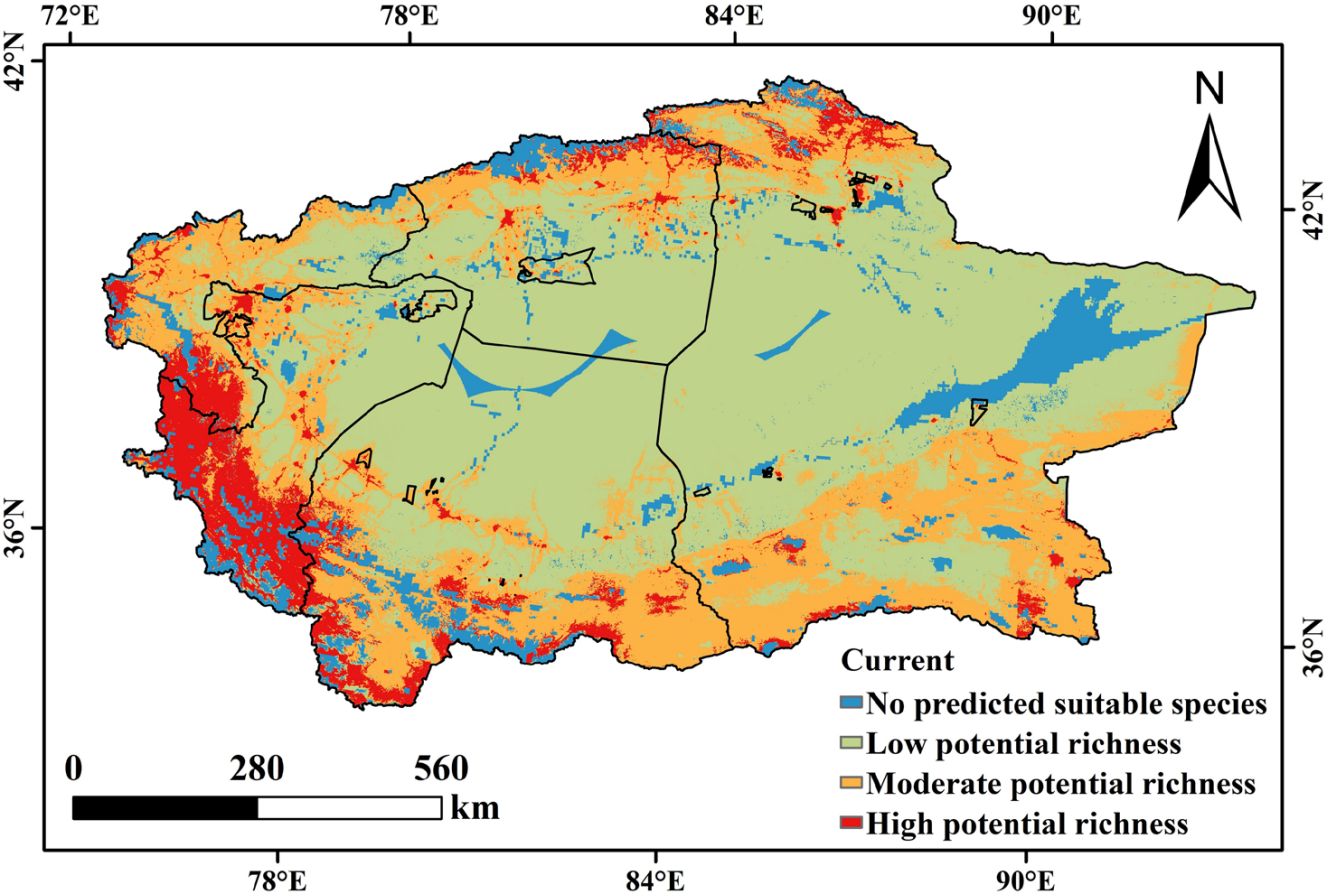
Spatial distribution of potential species richness under current climate scenarios.

Based on the spatial overlay of binary rasters for 608 species, the potential species richness pattern of wild plants in the Tarim Basin exhibited marked spatial heterogeneity (Figure 4). Under current climatic conditions, areas of high potential richness were primarily distributed in the western and southwestern mountainous regions and their northern margins, with localized clusters on the southern slopes of the Tianshan Mountains, the northern foothills of the Kunlun Mountains, and the foreland oasis‐desert transition zones. Areas of moderate potential richness were mostly distributed along mountain‐basin transition zones and basin margins. Areas of low potential richness were more widely distributed, mainly along the basin margins and surrounding regions. Areas with no predicted suitable species were concentrated in the interior of the Tarim Basin, with scattered occurrences along the edges of some high‐altitude mountains and in extremely arid areas. Overall, the spatial pattern of wild plants in the Tarim Basin is characterized by higher richness in mountainous and foothill transition zones and lower richness in the basin interior.

**FIGURE 5:**
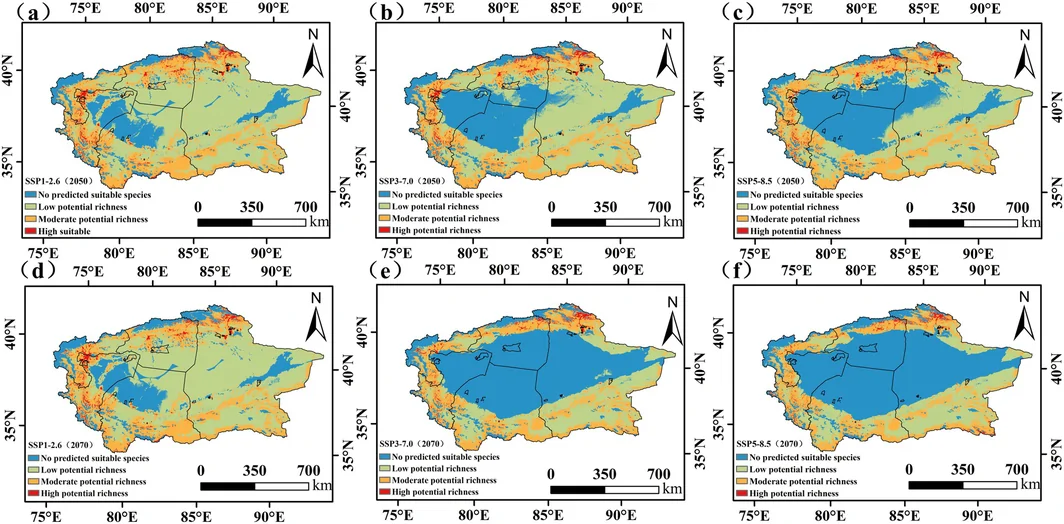
Spatial distribution of potential species richness under future climate scenarios. Panels (a–c): 2050s under SSP1‐2.6, SSP3‐7.0, and SSP5‐8.5; panels (d–f): 2070s under SSP1‐2.6, SSP3‐7.0, and SSP5‐8.5.

**FIGURE 6:**
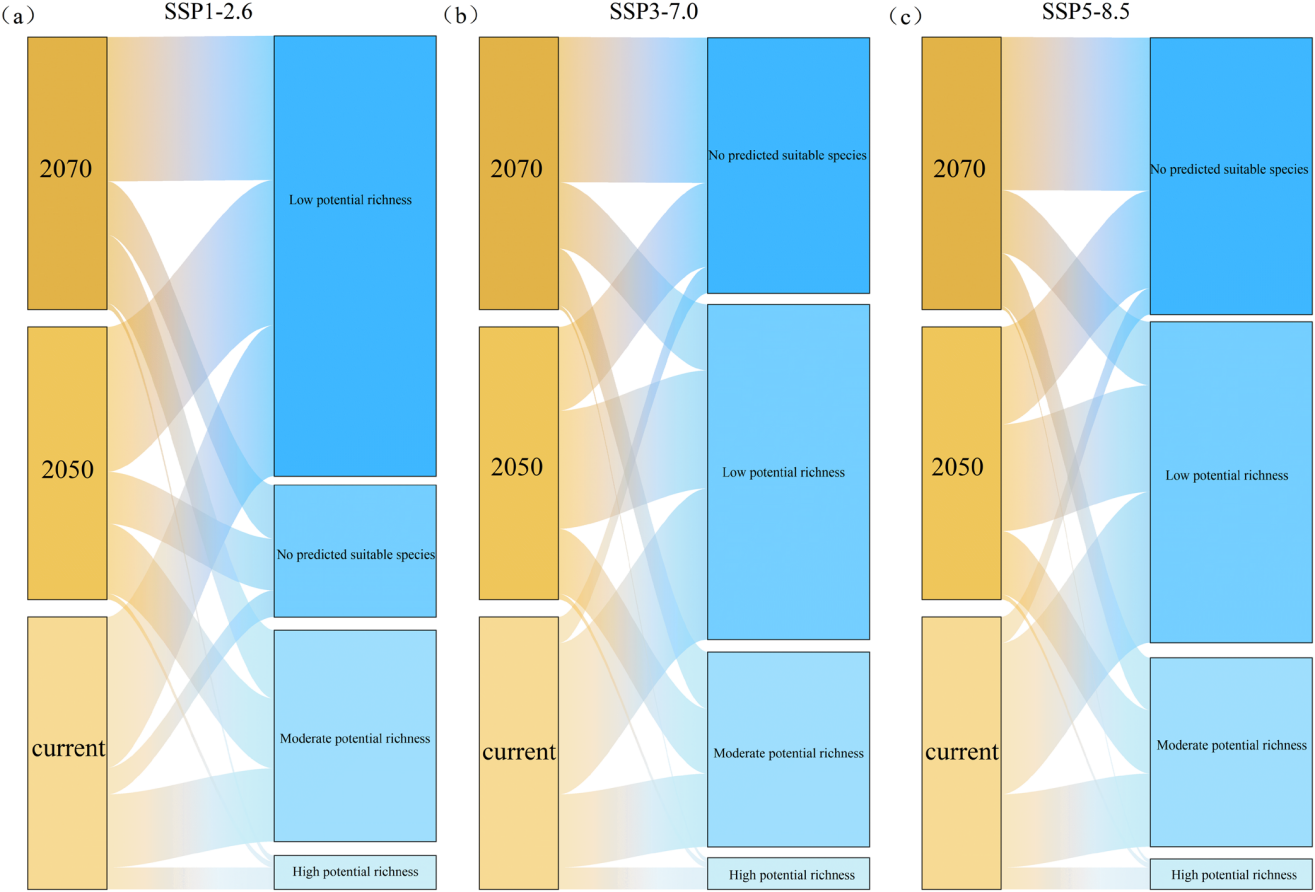
Changes in the area structure of potential species richness classes under different climate scenarios. Panels (a–c) correspond to SSP1‐2.6, SSP3‐7.0, and SSP5‐8.5.

Under future climate scenarios, potential richness patterns remained largely consistent with current conditions, but richness levels and area structure showed substantial adjustments (Figures 5 and 6). Areas of low potential richness continued to account for a large proportion under all scenarios. Areas with no predicted suitable species showed an overall expansion trend, which was most pronounced under the SSP3‐7.0 and SSP5‐8.5 scenarios in the 2070s. Areas of high potential richness continued to shrink and became increasingly fragmented, while areas of moderate potential richness exhibited relatively gradual changes.

**FIGURE 7:**
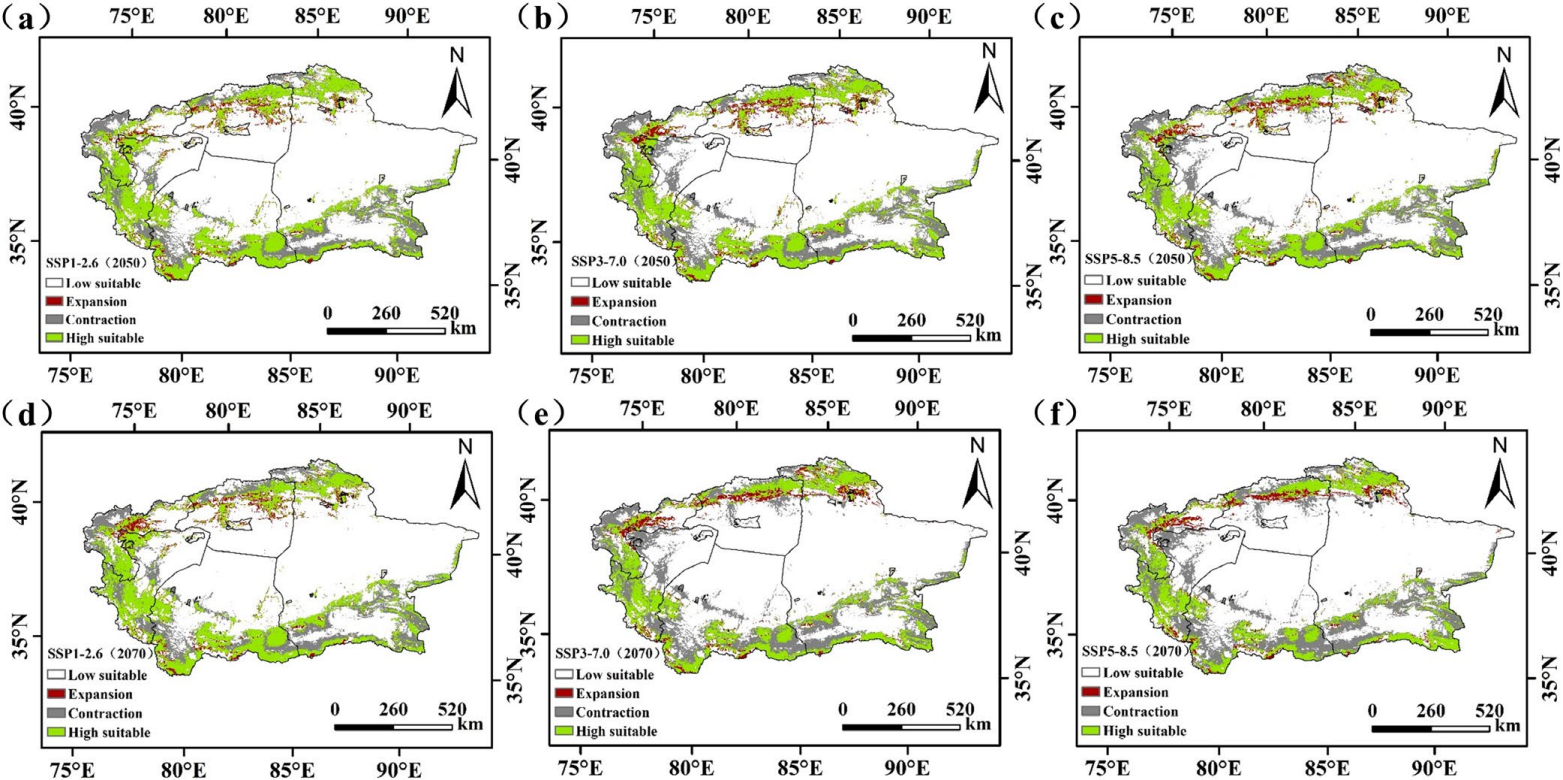
Spatial distribution of changes in potential species richness under different climate scenarios. Panels (a–c): 2050s under SSP1‐2.6, SSP3‐7.0, and SSP5‐8.5; panels (d–f): 2070s under SSP1‐2.6, SSP3‐7.0, and SSP5‐8.5.

The results on changes in potential richness indicate that future trends will be dominated by a decline in potential richness. Declining areas were primarily located along basin margins, in mountain‐basin transition zones, and on the peripheries of current areas of moderate and high potential richness, while increasing areas were mostly scattered along local mountain margins and in oasis‐desert transition zones (Figure 7). Overall, as emission scenarios intensify and time progresses, the potential richness of wild plants in the Tarim Basin shows a trend toward decline and fragmentation.

**FIGURE 8:**
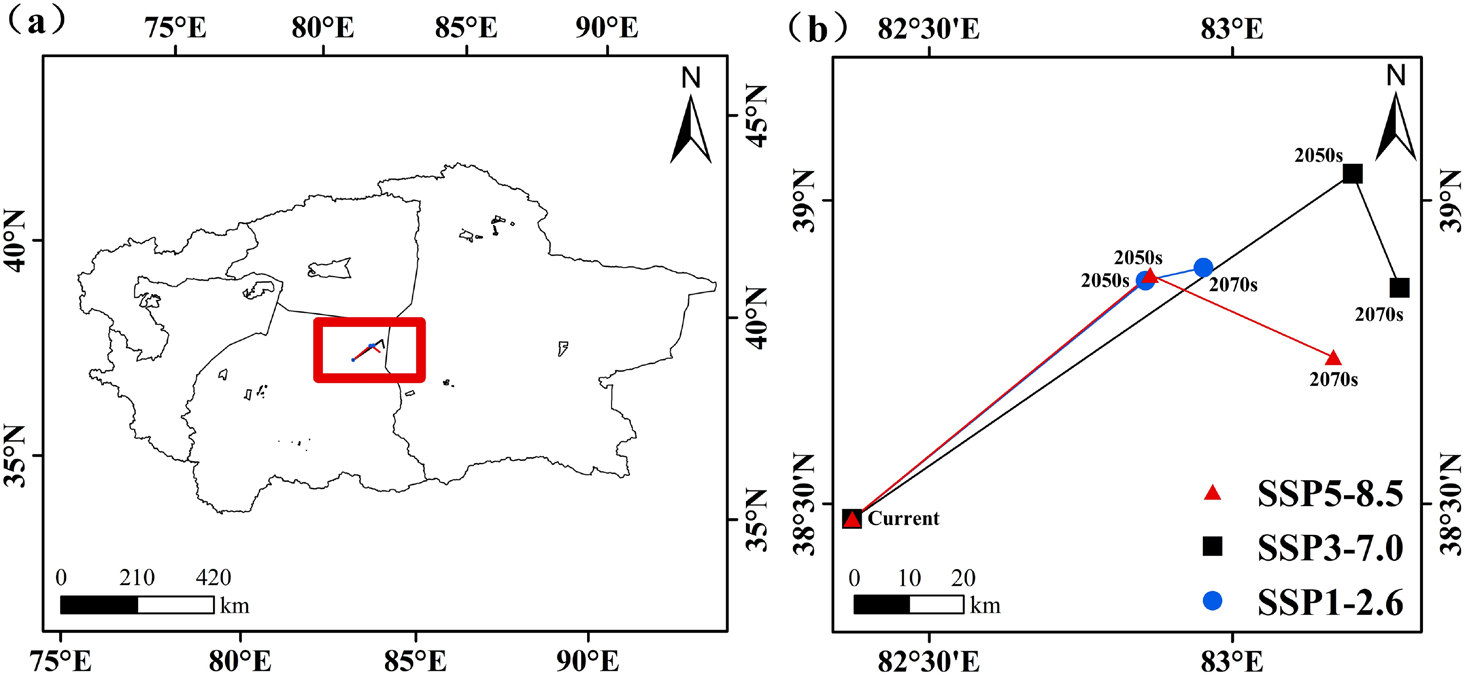
Shifts in the centroid of potential species richness under different climate scenarios. (a) Study area and centroid locations; (b) centroid trajectories under SSP1‐2.6, SSP3‐7.0, and SSP5‐8.5 from the current period to the 2050s and 2070s.

Results on the shift of the centroid of potential richness indicate that, under various future climate scenarios, the centroid will generally shift toward the northeast (Figure 8). From the present to the 2050s, the centroid shifts northeastward across all scenarios, with a relatively larger shift under SSP3‐7.0. By the 2070s, the centroid continues to shift slightly northeastward under SSP1‐2.6, whereas under SSP3‐7.0 and SSP5‐8.5, the centroid shows a temporary southeastward adjustment. Although the direction of the shift in the later stage varies among scenarios, the centroid under all future scenarios generally remains northeast of the current centroid.

### 3.3 Relative Contributions of Environmental Factors

**FIGURE 9:**
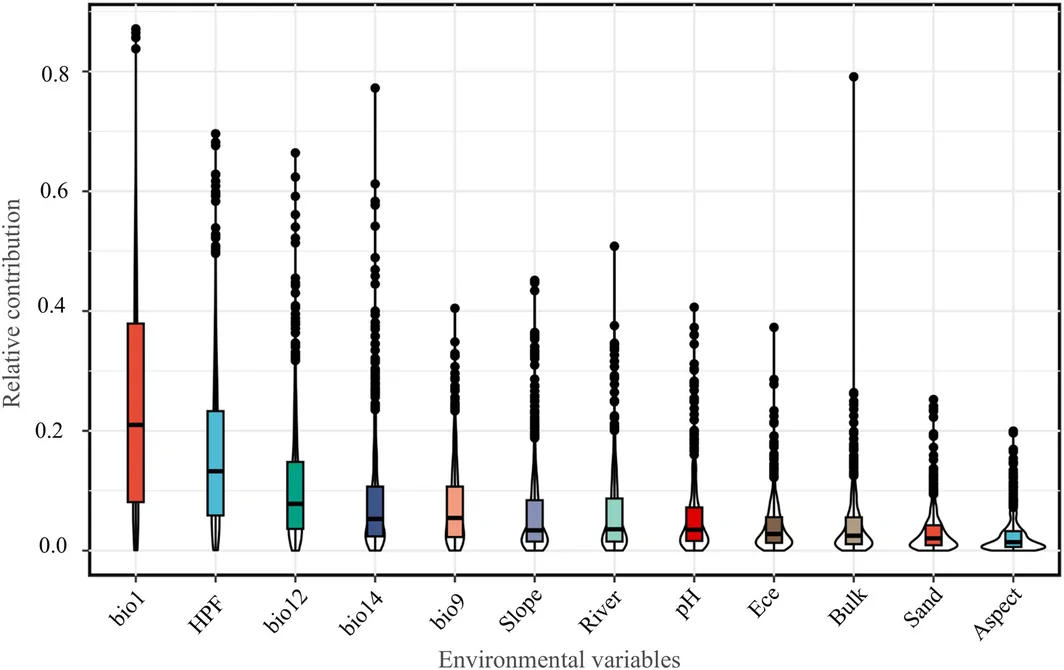
Relative contributions of environmental factors based on a summary of 608 single‐species models.

The relative contributions of environmental factors, based on a summary of the 608 single‐species models, showed substantial differences among variables (Figure 9). Climatic variables dominated, with bio1 having the highest contribution, substantially higher than that of other environmental factors, followed by HPF, bio12, bio14, and bio9. In contrast, topographic and soil variables such as slope, pH, and bulk density had lower contributions. Overall, potential suitable habitats of wild plants in the Tarim Basin are primarily influenced by climatic factors, while human activities also play an important role.

### 3.4 Shrinkage of Potential Suitable Habitats and Habitat Loss Risk

**FIGURE 10:**
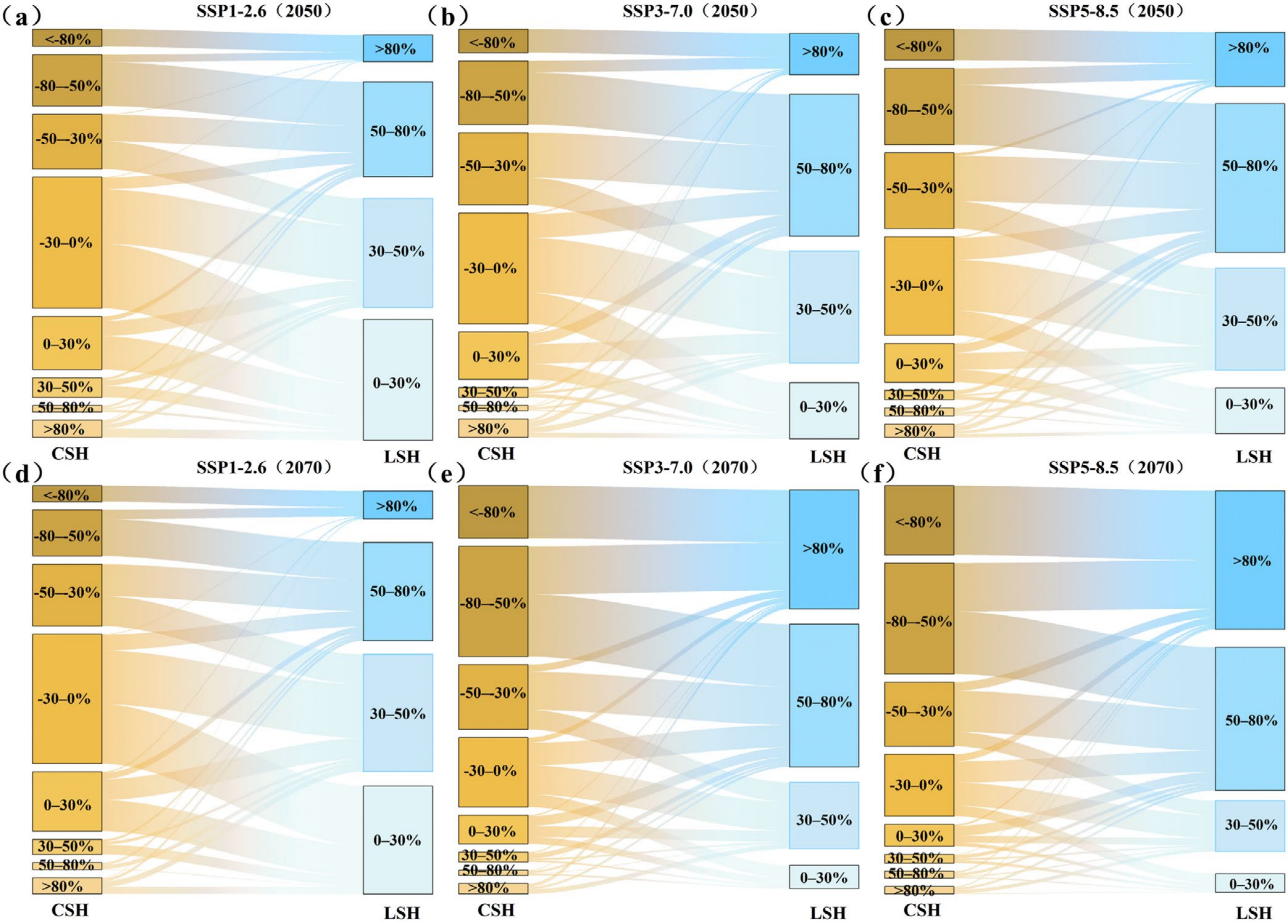
Conversion relationship between change in percentage change in potential suitable habitat (CSH) and percentage loss of habitat (LSH) under different climate scenarios. Panels (a–c): 2050s under SSP1‐2.6, SSP3‐7.0, and SSP5‐8.5; panels (d–f): 2070s under SSP1‐2.6, SSP3‐7.0, and SSP5‐8.5.

Under future climate scenarios, the potential suitable habitats of wild plants in the Tarim Basin are generally projected to contract (Figure 10). Based on the relationship between CSH and LSH, most species shift from negative CSH levels to medium‐to‐high LSH levels, indicating that the contraction of potential suitable habitats is typically accompanied by increased habitat loss risk. Differences across scenarios were pronounced. Under SSP1‐2.6, species primarily experienced mild to moderate contraction, with relatively low LSH levels and generally moderate overall changes. Under SSP3‐7.0, potential habitat contraction and habitat loss were substantially amplified, increasing further by the 2070s. Changes were most pronounced under SSP5‐8.5, with more species exhibiting strong contraction and higher habitat loss levels. Overall, as emission scenarios intensify and time progresses, the contraction of potential suitable habitats and the risk of habitat loss show an escalating trend.

**FIGURE 11:**
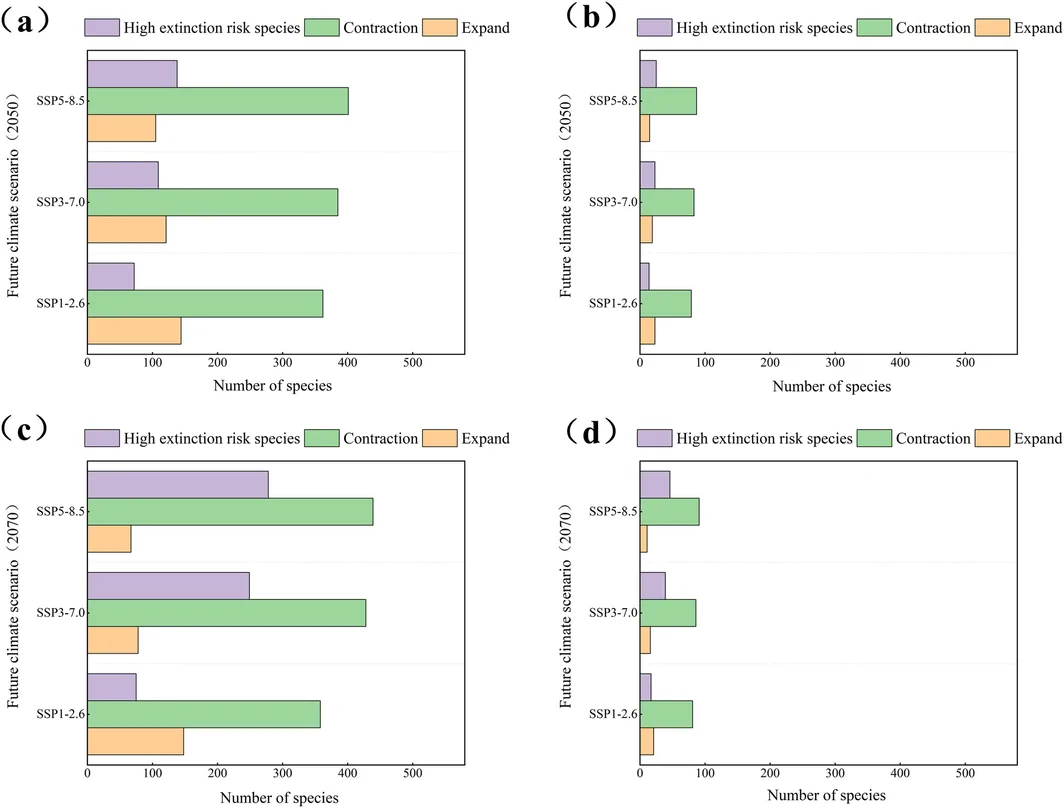
Number comparison of high extinction‐risk species, species with contracted suitable habitats, and species with expanded suitable habitats for herbaceous and woody plants under different climate scenarios. Panels (a) and (c) represent herbaceous plants in the 2050s and 2070s, respectively; panels (b) and (d) represent woody plants in the 2050s and 2070s, respectively.

When LSH ≥ 80% was used as the threshold for severe habitat loss, 28.8% of species experienced deterioration in their risk status, 70.9% remained stable, and only 0.3% showed improvement, indicating that future changes in species risk are generally dominated by adverse shifts (Table S2). To illustrate these changes at the species level, we selected 10 representative species of conservation or ecological relevance (Table 2). All 10 species exhibited deteriorating habitat‐loss risk from the 2050s to the 2070s. Risk scores decreased from 4 to 2 for *Phragmites australis* , Glycyrrhiza inflata, Populus euphratica, Populus pruinosa, *Tamarix ramosissima* , *Alhagi camelorum* , Lycium ruthenicum, and Myricaria pulcherrima, whereas the scores of Arnebia euchroma and Rhodiola gelida decreased from 4 to 3. These species include nationally or regionally protected taxa as well as dominant, structural, or associated species of characteristic desert‐riparian and oasis–desert transition communities in the Tarim Basin. Responses to future climate change differed markedly between life forms (Figure 11). In terms of species numbers, herbaceous plants exhibited more cases of potential suitable habitat contraction and more high‐risk species than woody plants, with these differences becoming more pronounced in the 2070s and under the SSP3‐7.0 and SSP5‐8.5 scenarios. For both life forms, contraction of potential suitable habitats was the dominant trend, while the number of species experiencing expansion was relatively small, indicating that the potential suitable habitats of most species will generally retreat under future climate change. Compared with woody plants, herbaceous plants exhibited stronger distributional responses.

**TABLE 2:** Representative plant species of conservation and ecological relevance showing deteriorating habitat‐loss risk from the 2050s to the 2070s in the Tarim Basin.

Species	Life form	Conservation and ecological relevance	Risk score (2050s)	Risk score (2070s)	Risk trend
*Phragmites australis*	Herbaceous	Dominant herbaceous species	4	2	Deterioration
*Glycyrrhiza inflata*	Herbaceous	Associated species; National Class II protected plant	4	2	Deterioration
*Arnebia euchroma*	Herbaceous	National Class II protected plant	4	3	Deterioration
*Rhodiola gelida*	Herbaceous	Xinjiang Class II protected plant	4	3	Deterioration
*Populus euphratica*	Woody	Key community‐building species; dominant tree species	4	2	Deterioration
*Populus pruinosa*	Woody	Dominant tree species in some riparian communities; Xinjiang Class II protected plant	4	2	Deterioration
*Tamarix ramosissima*	Woody	Dominant shrub species	4	2	Deterioration
*Alhagi camelorum*	Woody	Associated species in some riparian communities	4	2	Deterioration
*Lycium ruthenicum*	Woody	Associated shrub species; National Class II protected plant	4	2	Deterioration
*Myricaria pulcherrima*	Woody	Xinjiang Class II protected plant	4	2	Deterioration

*Note:* National and regional protection categories follow the List of National Key Protected Wild Plants (2021) and the List of Key Protected Wild Plants of the Xinjiang Uygur Autonomous Region (2023), respectively. Ecological roles were compiled from published field‐based plant community studies in the Tarim Basin. Risk scores were determined according to the occurrence of severe habitat loss (LSH ≥ 80%) under future climate scenarios; lower scores indicate higher habitat‐loss risk. “Deterioration” indicates a decrease in risk score from the 2050s to the 2070s.

### 3.5 Species Turnover Hotspots, Conservation Priority Areas, and Conservation Gaps

**FIGURE 12:**
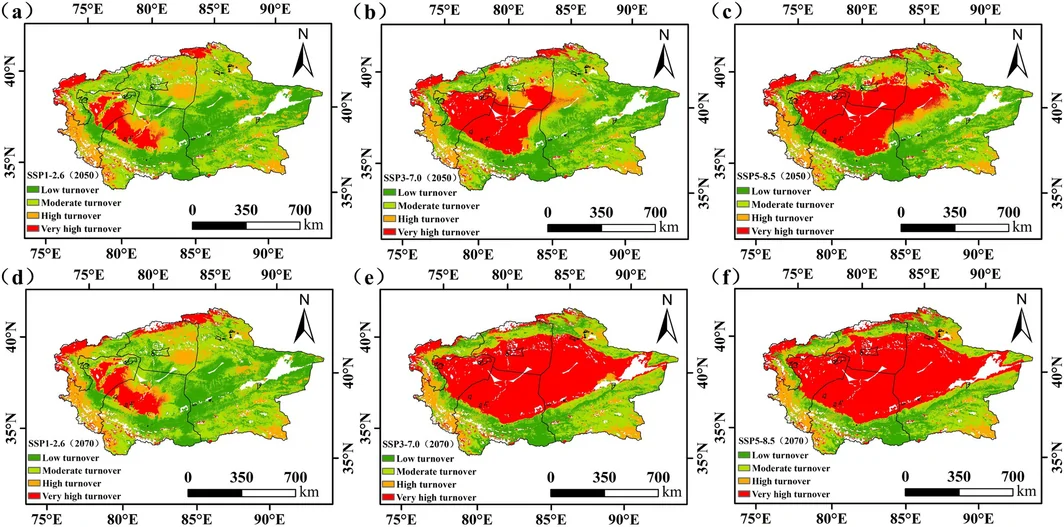
Spatial distribution of species turnover under different climate scenarios. Panels (a)–(c) represent SSP1‐2.6, SSP3‐7.0, and SSP5‐8.5 in the 2050s, respectively; panels (d)–(f) represent SSP1‐2.6, SSP3‐7.0, and SSP5‐8.5 in the 2070s, respectively.

Under different climate scenarios, the spatial distribution of species turnover rates exhibited substantial heterogeneity (Figure 12). Low‐turnover areas were primarily distributed in the eastern and southern peripheries of the study area and along some mountain edges. Moderate‐turnover areas were mostly located between low‐ and high‐turnover zones, forming a transitional belt. High‐turnover and very high‐turnover zones were mainly concentrated in the central‐western Tarim Basin, the northern foothill zone, and the oasis‐desert ecotones along the basin periphery, connecting with local foothill zones on the southern slopes of the Tianshan Mountains and the northern foothills of the Kunlun Mountains. Under the SSP3‐7.0 and SSP5‐8.5 scenarios, the extent of high‐turnover and very high‐turnover zones expanded substantially, forming a continuous distribution across the central and western parts of the basin. This trend intensified further by the 2070s, indicating that species composition changes will be more pronounced under high‐emission scenarios.

**FIGURE 13:**
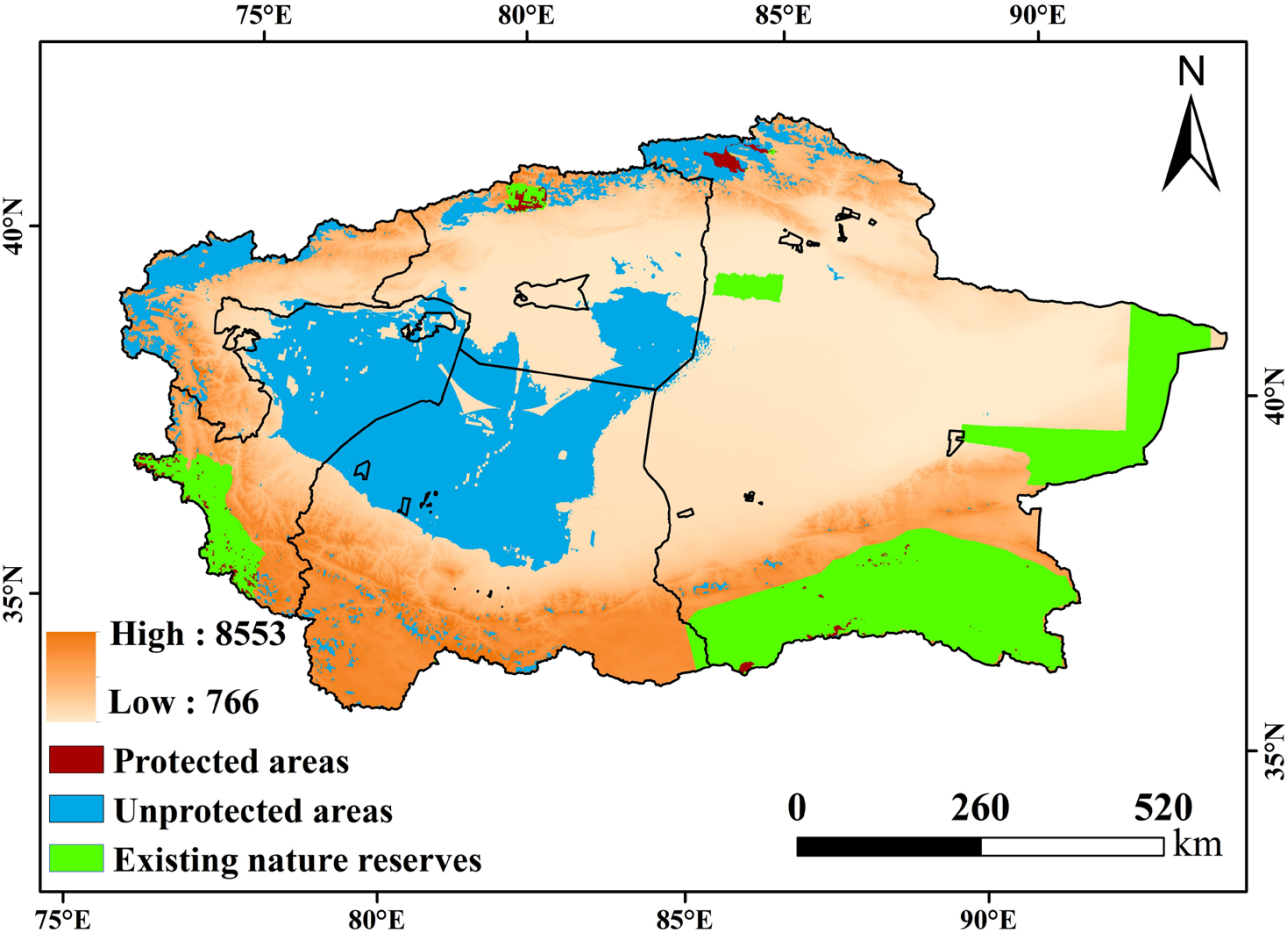
Overlay of conservation priority areas and existing nature reserves and identification of conservation gaps. The elevation background is shown using a light orange gradient. Red indicates areas within conservation priority areas already covered by existing nature reserves; blue indicates uncovered areas within conservation priority areas; green indicates existing nature reserves.

Of the conservation priority areas delineated based on species turnover rates, only 1.94% overlapped with existing nature reserves, while the remaining 98.06% constituted conservation gaps (Figure 13). Existing nature reserves are primarily distributed in the eastern, southern, and parts of the southwestern mountainous regions of the study area, whereas conservation gaps are mainly concentrated in the central‐western Tarim Basin, the northern foothill zone, and parts of the northern foothills of the Kunlun Mountains, where they overlap considerably with high‐turnover hotspots. These results indicate a clear spatial mismatch between the distribution of existing nature reserves and the key areas predicted to experience substantial future changes in species composition.

## 4 Discussion

### 4.1 Moisture and Thermal Constraints and the Effects of Human Activities on Potential Species Richness Patterns

The current pattern of potential species richness exhibits marked spatial heterogeneity, generally characterized by higher richness in mountainous areas and foothill transition zones and lower richness in the basin interior. Under various future climate scenarios, areas of high potential richness will continue to shrink and become increasingly fragmented, while areas of low potential richness and areas with no predicted suitable species will account for a large proportion. This suggests that climate change may cause areas suitable for the distribution of diverse plant species in the Tarim Basin to shrink and become more dispersed. The relative contribution analysis shows that bio1 contributes the most, while HPF, bio12, bio14, and bio9 also make substantial contributions. This indicates that the formation of the potential species richness pattern of wild plants in the Tarim Basin is closely related to the combined effects of moisture and thermal constraints and human activities. It should be noted that this study employed the target‐group background method to generate pseudo‐absence points to reduce spatial sampling bias. This method may make the background environment more similar to the species occurrence environment, thereby affecting the absolute importance values of individual variables. However, after multicollinearity screening, multi‐algorithm ensemble modeling, and strict accuracy control, the temperature variable exhibited a high contribution across different models, indicating that its importance has good stability and ecological interpretability.

Continued warming can increase regional evapotranspiration and further intensify water scarcity and aridification (Zhou et al. 2015). The highest contribution of bio1 suggests that thermal conditions may be an important climatic constraint influencing the potential richness patterns of wild plants in the Tarim Basin. In arid regions, temperature not only determines growing‐season length and metabolic intensity but also increases evapotranspiration demand, thereby exacerbating soil moisture deficits and reducing plant habitat suitability (Li, Yang, et al. 2014). The higher contribution of bio9 further indicates that extreme moisture and thermal conditions during the dry season also constitute a major bottleneck limiting the potential distribution of plants, consistent with the high sensitivity of arid‐region plants to extreme moisture and heat stress (Chen et al. 2021). In parallel with temperature variables, the high contributions of bio12 and bio14 indicate that plant distributions are jointly constrained by long‐term water availability and minimum moisture conditions during the driest period. Bio12 reflects long‐term regional water availability, whereas bio14 represents the lower moisture limit during the driest month. Together, these variables influence whether plants can complete their life cycles and maintain population regeneration. Arid‐zone plant communities are highly sensitive to annual precipitation, precipitation seasonality, and extreme drought events, which is consistent with the importance of water‐related variables in this study (Ma et al. 2025).

The high contribution of HPF suggests that human activities have become an important environmental factor in addition to climate. In the Tarim Basin, oasis agricultural development, irrigation expansion, and the concentration of transportation routes and settlements along river corridors have created typical linear and patchy disturbance patterns (Hu, Song, and Zhang 2025). Studies of global arid regions also show that climate change and human activities do not operate independently; anthropogenic disturbances such as agricultural development and landscape modification have become key drivers of biodiversity pattern shifts (Lewis et al. 2017). By contrast, topographic and soil variables contributed relatively little at the regional scale, but their ecological roles should not be overlooked (Wang and Peng 2022). Slope and aspect can alter microhabitats by regulating light and heat redistribution and water infiltration, whereas soil pH and texture influence plant survival by affecting microbial activity, root uptake, and nutrient availability (Yu et al. 2015). Although their contributions to large‐scale distribution modeling were relatively low, topography and soil can still buffer climatic stress at finer scales, especially in mountains, valleys, and oasis margins (Wos et al. 2021).

From a spatial perspective, the northern foothills of the Kunlun Mountains, the southern slopes of the Tianshan Mountains, and the foreland transition zone constitute areas with high potential species richness, primarily due to the moderating effect of mountainous terrain on local moisture and thermal conditions. Higher elevations and orographic precipitation provide more favorable hydrothermal conditions than those found in the basin interior. At the same time, variation in slope and aspect increases local microhabitat heterogeneity, allowing plants with different ecological niches to coexist (Chen, Kang, et al. 2025). Foothill oasis‐desert ecotones also receive continuous inputs from mountain runoff and groundwater, maintaining relatively high soil moisture and nutrient levels under regional aridity (Wang et al. 2019). In sharp contrast, the interior of the Tarim Basin is deeply inland, receives limited moisture transport, has extremely low precipitation, and experiences strong evapotranspiration, resulting in chronic soil moisture deficits. Its flat terrain provides little buffering against extreme hydrothermal stress, which strongly limits plant growth, dispersal, and population persistence. Frequent extreme heat events can suppress photosynthesis and increase transpiration‐driven water loss, further reducing the ability of species to maintain stable populations (Tu et al. 2023; Li, Tang, et al. 2014). Therefore, mountainous regions and foothill transition zones are not only areas with high potential species richness but also key regions for maintaining plant diversity and ecosystem stability under future climate change; their conservation value may increase further as the climate warms.

The centroid of the potential species richness pattern shifted overall toward the northeast, indicating that the distribution of multiple plant species may undergo a directional shift in the future. Although the centroid experienced a slight reversal in direction under the high‐emission scenarios in the mid‐to‐late periods, the overall direction of the shift remained fundamentally unchanged. This pattern may be related to spatial variations in moisture and thermal conditions in the Tarim Basin, the buffering effect of mountainous terrain against climatic stress, and directional changes in the potential suitable ranges of species. Rising temperatures and more frequent extreme climatic events can shorten plant growth cycles and reduce habitat suitability, a pattern widely reported across Eurasia (Duyar et al. 2025).

In summary, the potential wild plant species richness pattern in the Tarim Basin is not determined by a single climatic factor but results from the combined effects of water availability, thermal stress, extreme conditions during the dry season, human activities, and local topographic buffering. This multi‐scale environmental filtering pattern reflects the typical response characteristics of mountain‐oasis‐desert composite ecosystems to climate change and human disturbances. It should be noted that future projections rely on a single climate model and do not incorporate key ecological processes such as interspecific interactions, dispersal limitations, and adaptive evolution. Therefore, the results are more suitable for characterizing changes in potential species richness patterns than for providing absolute predictions of future actual species distributions.

### 4.2 Shrinkage of Potential Suitable Habitats and Species Composition Turnover Under Climate Change

Under future climate scenarios, the potential suitable habitats of most species in the Tarim Basin are projected to shrink overall. As emission scenarios become more severe and time progresses, habitat loss risk and the contraction of potential suitable habitats will intensify further. Moreover, future scenarios are generally expected to result in adverse changes, indicating that wild plants in the Tarim Basin face a high risk of habitat loss. Habitat loss and fragmentation can isolate populations in scattered patches, reduce dispersal and recolonization capacity, weaken adaptive potential under climate change, and increase the risk of local extinction (Hayes et al. 2024).

Risk responses differed clearly between life forms. Herbaceous plants were more sensitive to future climate change than woody plants. Previous studies have suggested that fast‐growing and short‐lived herbaceous plants often have lower tolerance to adverse conditions such as drought, soil alkalization, and nutrient limitation than woody plants (Wang et al. 2023). Their vulnerability is closely related to resource‐use strategies and life‐history traits, including stronger dependence on surface soil moisture, greater sensitivity to increased evapotranspiration, and stronger dependence of life‐history processes on interannual precipitation pulses. In contrast, woody plants generally have deeper root systems and greater resource storage capacity, allowing them to buffer soil drought stress to some extent (Jiao et al. 2026). However, the relative stability of woody plants does not necessarily indicate ecosystem improvement. Risk differences among life forms may alter dominance relationships within communities and thereby affect community structure, interspecific interactions, and ecosystem functioning. Field‐based vegetation studies identify *P. euphratica*, *T. ramosissima* , and *P. australis* as major components of desert‐riparian communities, while *P. pruinosa* , *G. inflata* , *L. ruthenicum*, and *Alhagi* spp. commonly occur as dominant or associated species (Zeng et al. 2020; Zhu et al. 2024; Huang et al. 2018). Therefore, increasing habitat‐loss risk for these species may alter not only individual species distributions but also community composition, dominance relationships, and structural stability. The concurrent deterioration of dominant and associated species suggests that characteristic desert‐riparian and oasis–desert transition communities may face increasing reorganization pressure under future climate change.

Areas with high and very high turnover are primarily concentrated in the central‐western Tarim Basin, the northern foothill zone, and the oasis‐desert ecotones along the basin periphery, and are connected to local foothill zones on the southern slopes of the Tianshan Mountains and the northern foothills of the Kunlun Mountains. These regions are predominantly situated within ecological transition zones between mountains, oases, and deserts. They are influenced by mountain runoff, groundwater recharge, and local microtopography, while also facing combined pressures from human activities within the basin and climate change. Consequently, they are not only currently predicted to be areas with relatively high potential species richness but are also the regions where future changes in species composition are expected to be most dynamic and sensitive. Species distributed at high elevations or near regional margins often face higher extinction risk because available migration space is limited and because they are constrained by elevation compression, mountain barriers, and geographic boundaries. For example, in the Andes, many species face a risk of “mountaintop extinction” because they lack additional space for upslope migration (Jiménez‐Mejías et al. 2026). If suitable climatic space continues to shift toward higher elevations or more remote areas, these species may experience a habitat trap caused by the exhaustion of available migration space. Although migration to higher elevations or latitudes is widely regarded as an important response to global warming, many species may still experience substantial habitat loss during migration (Zhang et al. 2024). Therefore, topography and geographic location are not merely contextual factors that shape the potential distribution patterns of species; they are also critical constraints that determine whether species will be able to maintain their populations through migration in the future.

Overall, the impact of climate change on wild plants in the Tarim Basin manifests through several interrelated processes, including contraction of potential suitable habitats, increased habitat loss risk, divergence in life‐form responses, and enhanced species composition turnover. Among these, herbaceous plants exhibit greater climate sensitivity than woody plants, whereas the mountain‐basin transition zone, the foothill oasis‐desert interface, and the contiguous central‐western basin areas are likely to become the primary regions for future plant community restructuring due to significant species turnover. This suggests that future climate change will not only alter the potential suitable ranges of various species but may also further reshape the structure of regional plant communities through differences in life‐form responses and species turnover.

### 4.3 Conservation Gaps and Dynamic Adaptive Priority Conservation Zones

The overlap between conservation priority areas identified based on species turnover and existing nature reserves is only 1.94%, and large areas of conservation gaps overlap considerably with high‐turnover hotspots, indicating a clear spatial mismatch between existing reserves and key areas for future biodiversity restructuring. Existing nature reserves are primarily established based on the current distribution of rare and endangered species, typical ecosystem types, or administrative feasibility; they essentially represent static, status quo‐oriented conservation. In contrast, future high‐turnover areas reflect the regions where species composition is expected to change most actively under climate change. The two differ substantially in terms of temporal scale and spatial orientation. Global studies have similarly shown that many protected areas are located in regions with relatively low human pressure and do not necessarily coincide with areas where biodiversity faces the greatest threats (Pulido‐Chadid et al. 2025).

Based on the above findings, it is necessary for the wild plant conservation strategy in the Tarim Basin to gradually shift from the current static conservation approach to a dynamic, adaptive conservation approach that accounts for future changes. To achieve dynamic adaptive conservation, priority should be given to filling gaps in spatial coverage in areas with high turnover. At the same time, species migration should be facilitated by enhancing ecological connectivity along environmental gradients (Hosseini et al. 2025). Specifically, the contiguous areas along the northern foothills of the Kunlun Mountains, the southern slopes of the Tianshan Mountains, and the central and western parts of the Tarim Basin should be prioritized as spatial units in future conservation planning. The spatial transition zones between these areas and existing protected areas represent potential priority areas for boundary optimization and corridor selection. Furthermore, areas within high‐turnover conservation gaps that have a high proportion of herbaceous vegetation may exhibit greater climate sensitivity and higher habitat loss risk; these should be prioritized as spatial units for monitoring and adaptive management within a dynamic conservation network. However, spatial prioritization alone is insufficient, and species‐level conservation targets also warrant explicit attention. All 10 representative plant species selected in this study for their conservation or ecological importance showed increasing habitat‐loss risk from the 2050s to the 2070s (Table 2). *Myricaria pulcherrima*, which is listed as a Xinjiang Class II protected wild plant, has small and fragmented populations and is threatened by habitat degradation and anthropogenic disturbance (Liu et al. 2020). *Arnebia euchroma* is a Class II nationally protected wild plant, and its total potential suitable habitat has been projected to show an overall decreasing trend under future climate scenarios (Shang et al. 2025). At the same time, ecologically important species such as *Populus euphratica*, *Tamarix ramosissima* , and *Phragmites australis* are dominant or structurally important components of typical riparian and oasis–desert transition communities in the Tarim Basin (Table 1). Therefore, future conservation planning should integrate spatial priority areas with species‐level conservation targets, giving attention to both legally protected species and ecologically key species, while maintaining the integrity of their associated plant communities and habitats.

This study has not yet incorporated factors such as conservation management effectiveness and land‐use change. Therefore, while the findings can provide a scientific basis for regional‐scale conservation planning, they still need to be further refined in actual management practices through the integration of multi‐source data and comprehensive assessments. For the Tarim Basin region, the mountain‐basin transition zone and the oasis‐desert interface serve not only as critical areas for maintaining biodiversity but also as key corridors for species migration and community restructuring under future climate change. Therefore, synergistic optimization should be pursued across three dimensions—spatial coverage, ecological connectivity, and long‐term monitoring—to enhance the climate resilience of plant diversity conservation in extremely arid regions.

## 5 Conclusions

Based on multi‐source distribution data and an ensemble species distribution model, this study systematically assessed potential species richness patterns, changes in potential suitable habitats, habitat loss risks, species turnover hotspots, and conservation gaps for 608 wild plant species in the Tarim Basin of China under current and future climate scenarios. The results indicate that, based on the aggregated environmental factor contributions from the 608 single‐species models, bio1, HPF, bio12, bio14, and bio9 made the highest contributions. This indicates that predictions of potential suitable habitats for wild plants in the Tarim Basin are primarily constrained by moisture and thermal conditions and are further influenced by human activities.

The current potential species richness pattern exhibits significant spatial heterogeneity, generally characterized by higher values in mountainous and foothill transition zones and lower values in the basin interior. Future climate change will lead to continued shrinkage and fragmentation of areas with high potential richness, while areas with low potential richness and those with no predicted suitable species will account for a larger proportion. Furthermore, the centroid of the potential species richness pattern will shift overall toward the northeast. Additionally, as emission scenarios intensify and time progresses, the potential suitable habitats of most species will contract, further exacerbating habitat loss risk; among them, herbaceous plants exhibit greater climate sensitivity than woody plants.

Species turnover hotspots are primarily concentrated in the central‐western Tarim Basin, the northern foothill zone, and the oasis‐desert ecotones along the basin periphery. The low overlap between conservation priority areas identified based on species turnover hotspots and existing nature reserves indicates a substantial spatial mismatch between the current layout of nature reserves and regions where future species composition is expected to undergo major changes. Future conservation of wild plants in the Tarim Basin should gradually shift from static, representative conservation to dynamic, adaptive conservation, focusing on the mountain‐basin transition zone, the foothill oasis‐desert interface, and high‐turnover conservation gaps, while strengthening ecological connectivity and long‐term dynamic monitoring. Importantly, the 10 representative species of conservation or ecological relevance examined in this study all showed deteriorating habitat‐loss risk from the 2050s to the 2070s, and several are dominant or associated components of characteristic desert‐riparian and oasis–desert transition communities. These findings indicate that future conservation planning should integrate spatial priority areas with species‐level protection and the maintenance of key plant communities.

## Author Contributions

**Qiong Chen:** conceptualization (lead), data curation (lead), formal analysis (lead), investigation (lead), methodology (lead), software (lead), visualization (lead), writing – original draft (lead), writing – review and editing (lead). **Ziwei Dou:** conceptualization (equal), data curation (equal), formal analysis (equal), investigation (equal), methodology (equal), software (equal), visualization (equal), writing – original draft (equal), writing – review and editing (equal). **Chunxia Wei:** data curation (supporting), investigation (supporting), software (supporting). **Lijun Zhu:** data curation (supporting), investigation (supporting), software (supporting). **Jie Wang:** data curation (supporting), investigation (supporting), software (supporting). **Bei Li:** data curation (supporting), investigation (supporting). **Juntuan Zhai:** conceptualization (equal), project administration (equal), resources (equal), supervision (equal), writing – review and editing (equal). **Zhijun Li:** conceptualization (lead), funding acquisition (lead), project administration (lead), resources (lead), supervision (lead), writing – review and editing (equal).

## Funding

This research was funded by the Xinjiang Uygur Autonomous Region “Tianshan Talents” Support Program—Science and Technology Innovation Team, grant number 2023TSYCTD0019.

## Conflicts of Interest

The authors declare no conflicts of interest.

## Supporting information


**Table S1:** Complete inventory of the 2110 wild plant species initially compiled for this study and their inclusion in the final modeling dataset.

**Table S2:** Habitat‐loss risk scores and temporal risk shifts of the 608 modeled wild plant species under future climate scenarios.

## Data Availability

The quality‐controlled species occurrence data, metadata, R script for the species distribution modeling workflow, and Tables S1 and S2 supporting this study are available in the Dryad Digital Repository at https://doi.org/10.5061/dryad.41ns1rhwq. Publicly available environmental datasets were obtained from the sources cited in the Methods section and were used in accordance with their respective terms of use.

## References

[ece374393-bib-0001] Aiello‐Lammens, M. E. , R. A. Boria , A. Radosavljevic , B. Vilela , and R. P. Anderson . 2015. “spThin: An R Package for Spatial Thinning of Species Occurrence Records for Use in Ecological Niche Models.” Ecography 38, no. 5: 541–545. 10.1111/ecog.01132.

[ece374393-bib-0002] Biancolini, D. , M. Pacifici , M. Falaschi , et al. 2024. “Global Distribution of Alien Mammals Under Climate Change.” Global Change Biology 30, no. 11: e17560. 10.1111/gcb.17560. 39545282

[ece374393-bib-0003] Cao, G. , X. Yuan , Q. Shu , et al. 2024. “Prediction of the Potentially Suitable Areas of *Eucommia ulmoides* Oliver in China Under Climate Change Based on Optimized Biomod2 and MaxEnt Models.” Frontiers in Plant Science 15: 1359271. 10.3389/fpls.2024.1359271. PMC11604462 39619845

[ece374393-bib-0004] Čengić, M. , J. Rost , D. Remenska , J. H. Janse , M. A. J. Huijbregts , and A. M. Schipper . 2020. “On the Importance of Predictor Choice, Modelling Technique, and Number of Pseudo‐Absences for Bioclimatic Envelope Model Performance.” Ecology and Evolution 10, no. 21: 12307–12317. 10.1002/ece3.6859. PMC7663074 33209289

[ece374393-bib-0005] Chen, K. , W. Shao , Y. Li , et al. 2025. “Biomod2 Modeling for Predicting Suitable Distribution of Bamboo Bat ( *Tylonycteris pachypus* ) Under Climate Change.” Animals 15, no. 8: 1164. 10.3390/ani15081164. PMC12023963 40281998

[ece374393-bib-0029] Chen, X. , N. B. Dimitrov , and L. A. Meyers . 2019. “Uncertainty Analysis of Species Distribution Models.” PLoS One 14, no. 5: e0214190. 10.1371/journal.pone.0214190. PMC6533036 31120909

[ece374393-bib-0006] Chen, Y. , Y. Kang , J. Li , et al. 2025. “Study on the Spatiotemporal Changes and Driving Factors of Habitat Quality in the Yarlung Zangbo River From 2000 to 2020.” Ecology and Evolution 15, no. 2: e70807. 10.1002/ece3.70807. PMC11783150 39896768

[ece374393-bib-0007] Chen, Y. , H. Zhang , L. Zhang , et al. 2021. “Is Plant Life‐History of Biseasonal Germination Consistent in Response to Extreme Precipitation?” Plants 10, no. 8: 1642. 10.3390/plants10081642. PMC8402233 34451688

[ece374393-bib-0008] Daru, B. H. 2024. “Predicting Undetected Native Vascular Plant Diversity at a Global Scale.” Proceedings of the National Academy of Sciences of the United States of America 121, no. 34: e2319989121. 10.1073/pnas.2319989121. PMC11348117 39133854

[ece374393-bib-0009] Duyar, A. , M. A. Demir , and M. Kabalak . 2025. “Prediction of Current and Future Distributions of *Chalcophora detrita* (Coleoptera: Buprestidae) Under Climate Change Scenarios.” Ecology and Evolution 15, no. 1: e70693. 10.1002/ece3.70693. PMC11739133 39830704

[ece374393-bib-0010] Hao, Y. , P. Dong , L. Wang , et al. 2024. “Predicting the Potential Distribution of *Hypericum perforatum* Under Climate Change Scenarios Using a Maximum Entropy Model.” Biology 13, no. 6: 452. 10.3390/biology13060452. PMC11201051 38927332

[ece374393-bib-0011] Hayes, M. P. , E. Ashe‐Jepson , G. E. Hitchcock , et al. 2024. “Heatwave Predicts a Shady Future for Insects: Impacts of an Extreme Weather Event on a Chalk Grassland in Bedfordshire, UK.” Journal of Insect Conservation 28, no. 5: 923–933. 10.1007/s10841-024-00556-5. PMC11489253 39430689

[ece374393-bib-0012] Hosseini, N. , A. Mehrabian , F. K. Nasab , H. Mostafavi , and M. Ghorbanpour . 2025. “Forecasting Climate Change Effects on the Potential Distribution of Zhumeria Majdae as an Endangered Monotypic Endemic Species: A Maxent Modeling Approach.” BMC Ecology and Evolution 25, no. 1: 85. 10.1186/s12862-025-02431-6. PMC12379370 40859141

[ece374393-bib-0013] Hu, J. , M. Song , and L. Zhang . 2025. “Spatial and Temporal Evolution of Land Use Carbon Emission and Carbon Balance Zoning: Evidence From Xinjiang China.” Scientific Reports 15, no. 1: 35705. 10.1038/s41598-025-19475-9. PMC12518828 41083561

[ece374393-bib-0014] Hu, S. , X. Ji , M. Wu , et al. 2025. “Taxonomical Characteristics of Rare and Endemic Plant Species and Identification of Their Protection Priority Area in the Tarim River Watershed, Xinjiang of Western China.” Journal of Beijing Forestry University 47, no. 6: 19–29. 10.12171/j.1000-1522.20240442.

[ece374393-bib-0015] Huang, J. , C. Liu , Z. Guo , et al. 2018. “Seed Plant Features, Distribution Patterns, Diversity Hotspots, and Conservation Gaps in Xinjiang, China.” Nature Conservation 27: 1–15. 10.3897/natureconservation.27.23728.

[ece374393-bib-0016] Huang, Y. , and X. M. Wang . 2018. “Analysis of Species Diversity of Typical Plant Communities in Oasis‐Desert Transition Zone of Tarim Basin Northern Margin.” Southwest China Journal of Agricultural Sciences 31, no. 3: 605–610. 10.16213/j.cnki.scjas.2018.3.031.

[ece374393-bib-0018] Jiao, C. , Z. He , J. Xu , X. Yi , J. Luo , and P. Huang . 2026. “Interannual Regime Shifts and Driver Thresholds of Terrestrial Ecosystem Vulnerability in Northwestern Sichuan of China Based on an XGBoost‐SHAP Model.” Biology 15, no. 4: 303. 10.3390/biology15040303. PMC12938296 41744612

[ece374393-bib-0019] Jiménez‐Mejías, P. , P. García‐Moro , A. A. Camilo , et al. 2026. “A Commented Checklist and Key for the Genus Carex (Cyperaceae) in Peru.” PhytoKeys 271: 93–144. 10.3897/phytokeys.271.178418. PMC12964055 41800223

[ece374393-bib-0020] Kang, Y. , F. Lin , J. Yin , et al. 2025. “Projected Distribution Patterns of *Alpinia officinarum* in China Under Future Climate Scenarios: Insights From Optimized Maxent and Biomod2 Models.” Frontiers in Plant Science 16: 1517060. 10.3389/fpls.2025.1517060. PMC11866951 40017818

[ece374393-bib-0021] Lan, Z. , L. Huiliang , Z. Hongxiang , et al. 2022. “Potential Distribution of Three Types of Ephemeral Plants Under Climate Changes.” Frontiers in Plant Science 13: 1035684. 10.3389/fpls.2022.1035684. PMC9728545 36507407

[ece374393-bib-0022] Lewis, J. S. , M. L. Farnsworth , C. L. Burdett , D. M. Theobald , M. Gray , and R. S. Miller . 2017. “Biotic and Abiotic Factors Predicting the Global Distribution and Population Density of an Invasive Large Mammal.” Scientific Reports 7, no. 1: 44152. 10.1038/srep44152. PMC5343451 28276519

[ece374393-bib-0023] Li, S. , Q. Tang , J. Lei , X. Xu , J. Jiang , and Y. Wang . 2014. “An Overview of Non‐Conventional Water Resource Utilization Technologies for Biological Sand Control in Xinjiang, Northwest China.” Environmental Earth Sciences 73, no. 2: 873–885. 10.1007/s12665-014-3443-y.

[ece374393-bib-0058] Li, X. , Y. Yang , X. Sun , et al. 2014. “Comparative Physiological and Proteomic Analyses of Poplar (*Populus yunnanensis*) Plantlets Exposed to High Temperature and Drought.” PLoS One 9, no. 9: e107605. 10.1371/journal.pone.0107605. PMC4167240 25225913

[ece374393-bib-0024] Li, Y. , Y. Ma , J. Liu , and J. Yang . 2023. “Analysis of the Spatial and Temporal Evolution of Urban Resilience in Four Southern Regions of Xinjiang.” International Journal of Environmental Research and Public Health 20, no. 6: 5106. 10.3390/ijerph20065106. PMC10049044 36982013

[ece374393-bib-0025] Li, Z. , A. Qiu , L. Zhang , et al. 2013. Native Plants Lists in Tarim Basin of Xinjiang. Science Press.

[ece374393-bib-0026] Liu, Y. P. , T. Y. Liu , and Z. Y. Zhu . 2020. “Investigation on the Endangered Causes of Endangered Plant *Myricaria pulcherrima* in Tarim Basin.” Journal of Anhui Agricultural Sciences 48, no. 16: 112–115. 10.3969/j.issn.0517-6611.2020.16.030.

[ece374393-bib-0027] Ma, H. , B. Wang , X. Yang , and G. Zhou . 2025. “Predicting the Potential Distribution of Lagotis Medicinal Plants on the Qinghai‐Xizang Plateau, With a Maximum Entropy Model.” Ecology and Evolution 15, no. 4: e71319. 10.1002/ece3.71319. PMC12015568 40270800

[ece374393-bib-0028] Ord, J. K. , and A. Getis . 1995. “Local Spatial Autocorrelation Statistics: Distributional Issues and an Application.” Geographical Analysis 27, no. 4: 286–306. 10.1111/j.1538-4632.1995.tb00912.x.

[ece374393-bib-0030] Perrino, E. V. , C. M. Musarella , and P. Magazzini . 2021. “Management of Grazing Italian River Buffalo to Preserve Habitats Defined by Directive 92/43/EEC in a Protected Wetland Area on the Mediterranean Coast: Palude Frattarolo, Apulia, Italy.” Euro‐Mediterranean Journal for Environmental Integration 6, no. 1: 32. 10.1007/s41207-020-00235-2.

[ece374393-bib-0031] Perrino, E. V. , V. Tomaselli , R. Costa , and P. Pavone . 2013. “Conservation Status of Habitats (Directive 92/43 EEC) of Coastal and Low Hill Belts in a Mediterranean Biodiversity Hot Spot (Gargano—Italy).” Plant Biosystems—An International Journal Dealing With All Aspects of Plant Biology 147, no. 4: 1006–1028. 10.1080/11263504.2013.860052.

[ece374393-bib-0032] Phillips, S. J. , M. Dudík , J. Elith , et al. 2009. “Sample Selection Bias and Presence‐Only Distribution Models: Implications for Background and Pseudo‐Absence Data.” Ecological Applications 19, no. 1: 181–197. 10.1890/07-2153.1. 19323182

[ece374393-bib-0033] Pound, K. L. , C. A. Larson , S. I. Passy , and T. Webb . 2020. “Current Distributions and Future Climate‐Driven Changes in Diatoms, Insects and Fish in U.S. Streams.” Global Ecology and Biogeography 30, no. 1: 63–78. 10.1111/geb.13193.

[ece374393-bib-0034] Pulido‐Chadid, K. , C. Rahbek , and J. Geldmann . 2025. “Evaluating Protected Areas' Coverage of Threats to Terrestrial Biodiversity.” Conservation Biology 39, no. 6: e70086. 10.1111/cobi.70086. PMC12658943 40490975

[ece374393-bib-0035] Roxo, G. , L. Silva , R. B. Elias , et al. 2026. “Red Listing the Flora of the Green Islands Reveals High Extinction Risk in the Azores.” Scientific Reports 16, no. 1: 22986. 10.1038/s41598-026-52252-w. PMC13392098 42162080

[ece374393-bib-0036] Shang, S. J. , D. H. Liu , Y. X. Zhou , J. J. Wu , T. Lu , and W. J. Li . 2025. “Prediction of the Suitable Distribution Areas of *Arnebia euchroma* (Boraginaceae) in China Under Climate Change Conditions.” Arid Zone Research 42, no. 9: 1628–1639. 10.13866/j.azr.2025.09.07.

[ece374393-bib-0037] Sun, S. , Y. Zhang , D. Huang , et al. 2020. “The Effect of Climate Change on the Richness Distribution Pattern of Oaks (*Quercus* L.) in China.” Science of the Total Environment 744: 140786. 10.1016/j.scitotenv.2020.140786. 32702540

[ece374393-bib-0038] Sun, Y. , Y. Deng , S. Yao , et al. 2025. “Distribution Range and Richness of Plant Species Are Predicted to Increase by 2100 due to a Warmer and Wetter Climate in Northern China.” Global Change Biology 31, no. 7: e70334. 10.1111/gcb.70334. PMC12232221 40613311

[ece374393-bib-0039] Thuiller, W. 2014. “Editorial Commentary on ‘BIOMOD – Optimizing Predictions of Species Distributions and Projecting Potential Future Shifts Under Global Change’.” Global Change Biology 20, no. 12: 3591–3592. 10.1111/gcb.12728. PMC4340559 25200636

[ece374393-bib-0040] Thuiller, W. , B. Lafourcade , R. Engler , and M. B. Araújo . 2009. “BIOMOD—A Platform for Ensemble Forecasting of Species Distributions.” Ecography 32, no. 3: 369–373. 10.1111/j.1600-0587.2008.05742.x.

[ece374393-bib-0041] Tu, Z. , Y. Zhou , J. Zhou , et al. 2023. “Identification and Risk Assessment of Priority Control Organic Pollutants in Groundwater in the Junggar Basin in Xinjiang, P.R. China.” International Journal of Environmental Research and Public Health 20, no. 3: 2051. 10.3390/ijerph20032051. PMC9915296 36767417

[ece374393-bib-0042] VanDerWal, J. , L. P. Shoo , C. Graham , and S. E. Williams . 2009. “Selecting Pseudo‐Absence Data for Presence‐Only Distribution Modeling: How Far Should You Stray From What You Know?” Ecological Modelling 220, no. 4: 589–594. 10.1016/j.ecolmodel.2008.11.010.

[ece374393-bib-0043] Venter, O. , E. W. Sanderson , A. Magrach , et al. 2016. “Global Terrestrial Human Footprint Maps for 1993 and 2009.” Scientific Data 3, no. 1: 160067. 10.1038/sdata.2016.67. PMC5127486 27552448

[ece374393-bib-0044] Wang, G. , and W. Peng . 2022. “Quantifying the Spatial Differentiation Mechanism of Land Use Degree.” Heliyon 8, no. 11: e11389. 10.1016/j.heliyon.2022.e11389. PMC9647443 36387544

[ece374393-bib-0045] Wang, R. , N. Wu , Z. Shi , et al. 2025. “Biomod2 for Evaluating the Changes in the Spatiotemporal Distribution of *Locusta migratoria tibetensis* Chen in the Qinghai‐Tibet Plateau Under Climate Change.” Global Ecology and Conservation 58: e03508. 10.1016/j.gecco.2025.e03508.

[ece374393-bib-0046] Wang, X. , M. Ji , Y. Zhang , et al. 2023. “Plant Trait Networks Reveal Adaptation Strategies in the Drylands of China.” BMC Plant Biology 23, no. 1: 266. 10.1186/s12870-023-04273-0. PMC10197480 37202776

[ece374393-bib-0047] Wang, Y. , M. Chen , L. Yan , G. Yang , J. Ma , and W. Deng . 2019. “Quantifying Threshold Water Tables for Ecological Restoration in Arid Northwestern China.” Groundwater 58, no. 1: 132–142. 10.1111/gwat.12934. 31407317

[ece374393-bib-0048] Wen, F. , S. Wu , X. Luo , L. Bai , and Z. Xia . 2024. “Microbial Community Structure in the Taklimakan Desert: The Importance of Nutrient Levels in Medium and Culture Methods.” Biology 13, no. 10: 797. 10.3390/biology13100797. PMC11505249 39452106

[ece374393-bib-0049] Wos, G. , M. Bohutínská , J. Nosková , T. Mandáková , and F. Kolář . 2021. “Parallelism in Gene Expression Between Foothill and Alpine Ecotypes in *Arabidopsis arenosa* .” Plant Journal 105, no. 5: 1211–1224. 10.1111/tpj.15105. 33258160

[ece374393-bib-0050] Xu, Y. , Z. Shen , L. Ying , et al. 2017. “Hotspot Analyses Indicate Significant Conservation Gaps for Evergreen Broadleaved Woody Plants in China.” Scientific Reports 7, no. 1: 1859. 10.1038/s41598-017-02098-0. PMC5431964 28500284

[ece374393-bib-0051] Yang, X. , W. Zhang , F. Qin , et al. 2022. “Biodiversity Priority Areas and Conservation Strategies for Seed Plants in China.” Frontiers in Plant Science 13: 962609. 10.3389/fpls.2022.962609. PMC9412182 36035703

[ece374393-bib-0052] Yang, Y. , Y. Ding , X. Peng , et al. 2026. “Integrating Habitat Suitability and Quality Assessments to Identify Conservation Priorities for *Cycas panzhihuaensis* .” Plants 15, no. 4: 670. 10.3390/plants15040670. PMC12943847 41754376

[ece374393-bib-0053] Yao, J. , W. Hu , Y. Chen , et al. 2019. “Hydro‐Climatic Changes and Their Impacts on Vegetation in Xinjiang, Central Asia.” Science of the Total Environment 660: 724–732. 10.1016/j.scitotenv.2019.01.084. 30743958

[ece374393-bib-0054] Ye, C. , K. Zhang , X. Gui , Y. Chen , and H. Meng . 2025. “Reassessment of the Conservation Status of the Endemic and Endangered Plant *Dalbergia odorifera* T.C. Chen in Hainan, China.” Ecology and Evolution 15, no. 11: e72465. 10.1002/ece3.72465. PMC12597129 41216495

[ece374393-bib-0017] Yu, L. , Y. Wang , Y. Wang , S. Sun , and L. Liu . 2015. “Quantifying Components of Soil Respiration and Their Response to Abiotic Factors in Two Typical Subtropical Forest Stands, Southwest China.” PLoS One 10, no. 2: e0117490. 10.1371/journal.pone.0117490. PMC4332482 25680112

[ece374393-bib-0055] Zeng, Y. , C. Zhao , F. Shi , M. Schneider , G. Lv , and Y. Li . 2020. “Impact of Groundwater Depth and Soil Salinity on Riparian Plant Diversity and Distribution in an Arid Area of China.” Scientific Reports 10, no. 1: 7272. 10.1038/s41598-020-64045-w. PMC7190620 32350302

[ece374393-bib-0056] Zhang, F.‐G. , S. Zhang , K. Wu , R. Zhao , G. Zhao , and Y. Wang . 2024. “Potential Habitat Areas and Priority Protected Areas of *Tilia amurensis* Rupr in China Under the Context of Climate Change.” Frontiers in Plant Science 15: 1365264. 10.3389/fpls.2024.1365264. PMC10978769 38559765

[ece374393-bib-0057] Zhang, H.‐X. , and M.‐L. Zhang . 2014. “Insight Into Distribution Patterns and Conservation Planning in Relation to Woody Species Diversity in Xinjiang, Arid Northwestern China.” Biological Conservation 177: 165–173. 10.1016/j.biocon.2014.07.005.

[ece374393-bib-0059] Zhang, L. , P. Wang , G. L. Xie , and W. K. Wang . 2025. “Assessing the Potential Distribution of Pseudoechthistatus (Coleoptera: Cerambycidae) in China Under Climate Change Using Species Distribution Models.” Ecology and Evolution 15, no. 4: e71303. 10.1002/ece3.71303. PMC11995185 40230865

[ece374393-bib-0060] Zhao, Y. , L. Zhang , and C. Wang . 2024. “Predicting Possible Distribution of Rice Leaf Roller (*Cnaphalocrocis medinalis*) Under Climate Change Scenarios Using MaxEnt Model in China.” Scientific Reports 14, no. 1: 21245. 10.1038/s41598-024-71228-2. PMC11391071 39261484

[ece374393-bib-0061] Zhou, Y. , X. Yu , R. Zhou , et al. 2025. “Climate Change and Human Activity Drive the Habitat of the Chinese Red Panda (*Ailurus styani*) to Expand to Higher Altitude.” Global Ecology and Conservation 64: e03971. 10.1016/j.gecco.2025.e03971.

[ece374393-bib-0062] Zhou, Y. , L. Zhang , R. Fensholt , K. Wang , I. Vitkovskaya , and F. Tian . 2015. “Climate Contributions to Vegetation Variations in Central Asian Drylands: Pre‐ and Post‐USSR Collapse.” Remote Sensing 7, no. 3: 2449–2470. 10.3390/rs70302449.

[ece374393-bib-0063] Zhu, C. , Z. Yang , X. Na , and X. Li . 2025. “Simulation of the Geographical Distribution of Global Potential Wetlands Under Climate Change Using an Ensemble Species Distribution Model.” Global Ecology and Conservation 63: e03862. 10.1016/j.gecco.2025.e03862.

[ece374393-bib-0064] Zhu, L. , J. Wang , H. Liu , J. Zhai , and Z. Li . 2024. “Community Assembly Mechanisms of *Populus euphratica* in Northwest China and Their Relationship With Environmental Factors.” Plants 13, no. 23: 3283. 10.3390/plants13233283. PMC11644819 39683077

[ece374393-bib-0065] Zizka, A. , D. Silvestro , T. Andermann , et al. 2019. “CoordinateCleaner: Standardized Cleaning of Occurrence Records From Biological Collection Databases.” Methods in Ecology and Evolution 10, no. 5: 744–751. 10.1111/2041-210x.13152.

