# Supplementary material for: Changes in Potential Suitable Habitats, Species Turnover, and Conservation Gaps of Wild Plants in the Tarim Basin, China
Source: Ecol Evol. 2026 Sep 23;16(9):e74393. doi: 10.1002/ece3.74393 (PMC13600769; doi:10.1002/ece3.74393)
Supplement: Supplementary file 1 [file ECE3-16-e74393-s001.docx]

**Supplementary Table S1.** Complete inventory of the 2,110 wild plant species initially compiled for this study and their inclusion in the final modeling dataset.

| Species | Family | Genus | Life form | Included in final modeling |
| --- | --- | --- | --- | --- |
| *Apocynum pictum* | Apocynaceae | *Apocynum* | Woody | Yes |
| *Lactuca tatarica* | Asteraceae | *Lactuca* | Herbaceous | Yes |
| *Cynanchum acutum subsp. sibiricum* | Apocynaceae | *Cynanchum* | Herbaceous | Yes |
| *Krascheninnikovia ceratoides* | Amaranthaceae | *Krascheninnikovia* | Woody | Yes |
| *Reaumuria songarica* | Tamaricaceae | *Reaumuria* | Woody | Yes |
| *Lycium ruthenicum* | Solanaceae | *Lycium* | Woody | Yes |
| *Tamarix ramosissima* | Tamaricaceae | *Tamarix* | Woody | Yes |
| *Sympegma regelii* | Amaranthaceae | *Sympegma* | Woody | Yes |
| *Populus euphratica* | Salicaceae | *Populus* | Woody | Yes |
| *Alhagi camelorum* | Fabaceae | *Alhagi* | Woody | Yes |
| *Peganum harmala* | Nitrariaceae | *Peganum* | Herbaceous | No |
| *Astragalus tibetanus* | Fabaceae | *Astragalus* | Herbaceous | Yes |
| *Convolvulus arvensis* | Convolvulaceae | *Convolvulus* | Herbaceous | Yes |
| *Elaeagnus angustifolia* | Elaeagnaceae | *Elaeagnus* | Woody | Yes |
| *Sphaerophysa salsula* | Fabaceae | *Sphaerophysa* | Woody | Yes |
| *Lepidium apetalum* | Brassicaceae | *Lepidium* | Herbaceous | No |
| *Inula salsoloides* | Asteraceae | *Inula* | Woody | No |
| *Phragmites australis* | Poaceae | *Phragmites* | Herbaceous | Yes |
| *Halogeton glomeratus* | Amaranthaceae | *Halogeton* | Herbaceous | No |
| *Plantago major* | Plantaginaceae | *Plantago* | Herbaceous | Yes |
| *Glycyrrhiza inflata* | Fabaceae | *Glycyrrhiza* | Herbaceous | Yes |
| *Ephedra przewalskii* | Ephedraceae | *Ephedra* | Woody | Yes |
| *Rosa laxa* | Rosaceae | *Rosa* | Woody | No |
| *Gentianella turkestanorum* | Gentianaceae | *Gentianella* | Herbaceous | Yes |
| *Tamarix hispida* | Tamaricaceae | *Tamarix* | Woody | Yes |
| *Braya humilis* | Brassicaceae | *Braya* | Herbaceous | Yes |
| *Polygonum aviculare* | Polygonaceae | *Polygonum* | Herbaceous | Yes |
| *Chenopodium album* | Amaranthaceae | *Chenopodium* | Herbaceous | No |
| *Calligonum roborowskii* | Polygonaceae | *Calligonum* | Woody | Yes |
| *Karelinia caspia* | Asteraceae | *Karelinia* | Herbaceous | Yes |
| *Arnebia guttata* | Boraginaceae | *Arnebia* | Herbaceous | Yes |
| *Oxytropis glabra* | Fabaceae | *Oxytropis* | Herbaceous | No |
| *Dracocephalum heterophyllum* | Lamiaceae | *Dracocephalum* | Herbaceous | Yes |
| *Myricaria bracteata* | Tamaricaceae | *Myricaria* | Woody | No |
| *Setaria viridis* | Poaceae | *Setaria* | Herbaceous | Yes |
| *Farinopsis salesoviana* | Rosaceae | *Farinopsis* | Woody | Yes |
| *Halogeton arachnoideus* | Amaranthaceae | *Halogeton* | Herbaceous | No |
| *Leymus secalinus* | Poaceae | *Leymus* | Herbaceous | Yes |
| *Cirsium arvense var. alpestre* | Asteraceae | *Cirsium* | Herbaceous | No |
| *Elaeagnus angustifolia var. angustifolia* | Elaeagnaceae | *Elaeagnus* | Woody | Yes |
| *Clematis orientalis* | Ranunculaceae | *Clematis* | Herbaceous | No |
| *Plantago depressa* | Plantaginaceae | *Plantago* | Herbaceous | Yes |
| *Sophora alopecuroides* | Fabaceae | *Sophora* | Woody | Yes |
| *Lepidium latifolium* | Brassicaceae | *Lepidium* | Herbaceous | Yes |
| *Clematis tangutica* | Ranunculaceae | *Clematis* | Woody | Yes |
| *Lysimachia maritima* | Primulaceae | *Lysimachia* | Herbaceous | Yes |
| *Juniperus pseudosabina* | Cupressaceae | *Juniperus* | Woody | Yes |
| *Oxybasis rubra* | Amaranthaceae | *Oxybasis* | Herbaceous | Yes |
| *Cirsium arvense* | Asteraceae | *Cirsium* | Herbaceous | No |
| *Neotrinia splendens* | Poaceae | *Neotrinia* | Herbaceous | No |
| *Nitraria sibirica* | Nitrariaceae | *Nitraria* | Woody | Yes |
| *Gentianopsis barbata* | Gentianaceae | *Gentianopsis* | Herbaceous | Yes |
| *Launaea polydichotoma* | Asteraceae | *Launaea* | Herbaceous | Yes |
| *Rhaponticum repens* | Asteraceae | *Rhaponticum* | Herbaceous | No |
| *Potentilla multifida* | Rosaceae | *Potentilla* | Herbaceous | Yes |
| *Bassia scoparia* | Amaranthaceae | *Bassia* | Herbaceous | Yes |
| *Triglochin palustris* | Juncaginaceae | *Triglochin* | Herbaceous | Yes |
| *Halostachys caspica* | Amaranthaceae | *Halostachys* | Woody | Yes |
| *Artemisia dracunculus* | Asteraceae | *Artemisia* | Herbaceous | Yes |
| *Salsola tragus* | Amaranthaceae | *Salsola* | Herbaceous | Yes |
| *Nitraria tangutorum* | Nitrariaceae | *Nitraria* | Woody | No |
| *Tamarix hohenackeri* | Tamaricaceae | *Tamarix* | Woody | Yes |
| *Grubovia dasyphylla* | Amaranthaceae | *Grubovia* | Herbaceous | No |
| *Leontopodium ochroleucum* | Asteraceae | *Leontopodium* | Herbaceous | Yes |
| *Carex kokanica* | Cyperaceae | *Carex* | Herbaceous | Yes |
| *Tamarix chinensis* | Tamaricaceae | *Tamarix* | Woody | Yes |
| *Solanum nigrum* | Solanaceae | *Solanum* | Herbaceous | Yes |
| *Zygophyllum xanthoxylum* | Zygophyllaceae | *Zygophyllum* | Woody | Yes |
| *Aster flaccidus* | Asteraceae | *Aster* | Herbaceous | Yes |
| *Elymus nutans* | Poaceae | *Elymus* | Herbaceous | Yes |
| *Argentina anserina* | Rosaceae | *Argentina* | Herbaceous | Yes |
| *Hippophae rhamnoides subsp. turkestanica* | Elaeagnaceae | *Hippophae* | Woody | No |
| *Sibbaldianthe bifurca* | Rosaceae | *Sibbaldianthe* | Herbaceous | Yes |
| *Zygophyllum fabago* | Zygophyllaceae | *Zygophyllum* | Herbaceous | Yes |
| *Richteria pyrethroides* | Asteraceae | *Richteria* | Herbaceous | Yes |
| *Equisetum ramosissimum* | Equisetaceae | *Equisetum* | Herbaceous | Yes |
| *Clematis songorica* | Ranunculaceae | *Clematis* | Herbaceous | Yes |
| *Allium oreoprasum* | Amaryllidaceae | *Allium* | Herbaceous | Yes |
| *Askellia flexuosa* | Asteraceae | *Askellia* | Herbaceous | Yes |
| *Berberis kaschgarica* | Berberidaceae | *Berberis* | Woody | Yes |
| *Lonicera microphylla* | Caprifoliaceae | *Lonicera* | Woody | Yes |
| *Tamarix arceuthoides* | Tamaricaceae | *Tamarix* | Woody | Yes |
| *Kalidium foliatum* | Amaranthaceae | *Kalidium* | Woody | Yes |
| *Atriplex centralasiatica* | Amaranthaceae | *Atriplex* | Herbaceous | Yes |
| *Melilotus albus* | Fabaceae | *Melilotus* | Herbaceous | No |
| *Populus pruinosa* | Salicaceae | *Populus* | Woody | Yes |
| *Caragana halodendron* | Fabaceae | *Caragana* | Woody | Yes |
| *Chloris virgata* | Poaceae | *Chloris* | Herbaceous | Yes |
| *Astragalus nivalis* | Fabaceae | *Astragalus* | Herbaceous | Yes |
| *Halocnemum strobilaceum* | Amaranthaceae | *Halocnemum* | Woody | Yes |
| *Oxybasis glauca* | Amaranthaceae | *Oxybasis* | Herbaceous | Yes |
| *Adenophora himalayana* | Campanulaceae | *Adenophora* | Herbaceous | Yes |
| *Lepidium obtusum* | Brassicaceae | *Lepidium* | Herbaceous | No |
| *Amaranthus retroflexus* | Amaranthaceae | *Amaranthus* | Herbaceous | Yes |
| *Aster altaicus* | Asteraceae | *Aster* | Herbaceous | Yes |
| *Tribulus terrestris* | Zygophyllaceae | *Tribulus* | Herbaceous | Yes |
| *Triglochin maritima* | Juncaginaceae | *Triglochin* | Herbaceous | Yes |
| *Glechoma hederacea* | Lamiaceae | *Glechoma* | Herbaceous | Yes |
| *Calamagrostis pseudophragmites* | Poaceae | *Calamagrostis* | Herbaceous | Yes |
| *Krascheninnikovia compacta* | Amaranthaceae | *Krascheninnikovia* | Herbaceous | Yes |
| *Christolea crassifolia* | Brassicaceae | *Christolea* | Herbaceous | Yes |
| *Agropyron cristatum* | Poaceae | *Agropyron* | Herbaceous | Yes |
| *Zygophyllum rosowii* | Zygophyllaceae | *Zygophyllum* | Herbaceous | Yes |
| *Capparis spinosa* | Capparaceae | *Capparis* | Woody | Yes |
| *Berberis nummularia* | Berberidaceae | *Berberis* | Woody | Yes |
| *Xanthium strumarium* | Asteraceae | *Xanthium* | Herbaceous | Yes |
| *Primula nutans* | Primulaceae | *Primula* | Herbaceous | Yes |
| *Iris lactea* | Iridaceae | *Iris* | Herbaceous | Yes |
| *Ephedra intermedia* | Ephedraceae | *Ephedra* | Woody | Yes |
| *Bistorta vivipara* | Polygonaceae | *Bistorta* | Herbaceous | Yes |
| *Androsace flavescens* | Primulaceae | *Androsace* | Herbaceous | Yes |
| *Limonium kaschgaricum* | Plumbaginaceae | *Limonium* | Herbaceous | Yes |
| *Caragana polourensis* | Fabaceae | *Caragana* | Woody | Yes |
| *Gymnocarpos przewalskii* | Caryophyllaceae | *Gymnocarpos* | Woody | Yes |
| *Carex capillifolia* | Cyperaceae | *Carex* | Herbaceous | Yes |
| *Asterothamnus fruticosus* | Asteraceae | *Asterothamnus* | Woody | Yes |
| *Hordeum brevisubulatum subsp. turkestanicum* | Poaceae | *Hordeum* | Herbaceous | Yes |
| *Medicago lupulina* | Fabaceae | *Medicago* | Herbaceous | Yes |
| *Anabasis aphylla* | Amaranthaceae | *Anabasis* | Woody | Yes |
| *Plantago minuta* | Plantaginaceae | *Plantago* | Herbaceous | Yes |
| *Glycyrrhiza glabra* | Fabaceae | *Glycyrrhiza* | Herbaceous | Yes |
| *Abutilon theophrasti* | Malvaceae | *Abutilon* | Herbaceous | Yes |
| *Parnassia laxmannii* | Celastraceae | *Parnassia* | Herbaceous | Yes |
| *Descurainia sophia* | Brassicaceae | *Descurainia* | Herbaceous | Yes |
| *Lomatogonium carinthiacum* | Gentianaceae | *Lomatogonium* | Herbaceous | Yes |
| *Geranium collinum* | Geraniaceae | *Geranium* | Herbaceous | Yes |
| *Calligonum mongolicum* | Polygonaceae | *Calligonum* | Woody | Yes |
| *Haloxylon ammodendron* | Amaranthaceae | *Haloxylon* | Woody | Yes |
| *Senecio krascheninnikovii* | Asteraceae | *Senecio* | Herbaceous | No |
| *Strigosella africana* | Brassicaceae | *Strigosella* | Herbaceous | Yes |
| *Polygonum cognatum* | Polygonaceae | *Polygonum* | Herbaceous | Yes |
| *Artemisia macrocephala* | Asteraceae | *Artemisia* | Herbaceous | Yes |
| *Artemisia sieversiana* | Asteraceae | *Artemisia* | Herbaceous | Yes |
| *Rudolf-kamelinia korolkowii* | Brassicaceae | *Rudolf-kamelinia* | Herbaceous | Yes |
| *Pedicularis cheilanthifolia* | Orobanchaceae | *Pedicularis* | Herbaceous | Yes |
| *Codonopsis clematidea* | Campanulaceae | *Codonopsis* | Herbaceous | Yes |
| *Elytrigia repens* | Poaceae | *Elytrigia* | Herbaceous | Yes |
| *Kalidium schrenkianum* | Amaranthaceae | *Kalidium* | Woody | Yes |
| *Caragana jubata* | Fabaceae | *Caragana* | Woody | Yes |
| *Hippophae rhamnoides* | Elaeagnaceae | *Hippophae* | Woody | Yes |
| *Polypogon monspeliensis* | Poaceae | *Polypogon* | Herbaceous | Yes |
| *Dracocephalum stamineum* | Lamiaceae | *Dracocephalum* | Herbaceous | Yes |
| *Taraxacum albiflos* | Asteraceae | *Taraxacum* | Herbaceous | Yes |
| *Euphrasia regelii* | Orobanchaceae | *Euphrasia* | Herbaceous | Yes |
| *Festuca olgae* | Poaceae | *Festuca* | Herbaceous | Yes |
| *Eragrostis minor* | Poaceae | *Eragrostis* | Herbaceous | Yes |
| *Salix alba* | Salicaceae | *Salix* | Woody | Yes |
| *Iris loczyi* | Iridaceae | *Iris* | Herbaceous | Yes |
| *Limonium aureum* | Plumbaginaceae | *Limonium* | Herbaceous | Yes |
| *Leontopodium leontopodioides* | Asteraceae | *Leontopodium* | Herbaceous | Yes |
| *Halerpestes sarmentosa* | Ranunculaceae | *Halerpestes* | Herbaceous | Yes |
| *Artemisia dracunculus var. pamirica* | Asteraceae | *Artemisia* | Herbaceous | Yes |
| *Ranunculus nephelogenes* | Ranunculaceae | *Ranunculus* | Herbaceous | Yes |
| *Glaucium squamigerum* | Papaveraceae | *Glaucium* | Herbaceous | Yes |
| *Lindelofia stylosa* | Boraginaceae | *Lindelofia* | Herbaceous | Yes |
| *Ephedra glauca* | Ephedraceae | *Ephedra* | Woody | Yes |
| *Stipa caucasica* | Poaceae | *Stipa* | Herbaceous | Yes |
| *Salix pycnostachya* | Salicaceae | *Salix* | Woody | Yes |
| *Salsola paulsenii* | Amaranthaceae | *Salsola* | Herbaceous | Yes |
| *Plantago arachnoidea* | Plantaginaceae | *Plantago* | Herbaceous | Yes |
| *Echinochloa crus-galli* | Poaceae | *Echinochloa* | Herbaceous | Yes |
| *Elaeagnus angustifolia var. orientalis* | Elaeagnaceae | *Elaeagnus* | Woody | Yes |
| *Rosa beggeriana* | Rosaceae | *Rosa* | Woody | Yes |
| *Salsola collina* | Amaranthaceae | *Salsola* | Herbaceous | Yes |
| *Acantholimon alatavicum* | Plumbaginaceae | *Acantholimon* | Woody | Yes |
| *Lepidium chalepense* | Brassicaceae | *Lepidium* | Herbaceous | Yes |
| *Scrophularia kiriloviana* | Scrophulariaceae | *Scrophularia* | Herbaceous | Yes |
| *Cirsium arvense var. integrifolium* | Asteraceae | *Cirsium* | Herbaceous | No |
| *Aster alpinus* | Asteraceae | *Aster* | Herbaceous | Yes |
| *Juncus articulatus* | Juncaceae | *Juncus* | Herbaceous | Yes |
| *Taraxacum mongolicum* | Asteraceae | *Taraxacum* | Herbaceous | Yes |
| *Atriplex sibirica* | Amaranthaceae | *Atriplex* | Herbaceous | Yes |
| *Melilotus suaveolens* | Fabaceae | *Melilotus* | Herbaceous | Yes |
| *Seriphidium rhodanthum* | Asteraceae | *Seriphidium* | Herbaceous | Yes |
| *Poa albertii* | Poaceae | *Poa* | Herbaceous | Yes |
| *Geranium pratense* | Geraniaceae | *Geranium* | Herbaceous | Yes |
| *Lepidium appelianum* | Brassicaceae | *Lepidium* | Herbaceous | Yes |
| *Potentilla pamiroalaica* | Rosaceae | *Potentilla* | Herbaceous | Yes |
| *Leontopodium pusillum* | Asteraceae | *Leontopodium* | Herbaceous | Yes |
| *Paropyrum anemonoides* | Ranunculaceae | *Paropyrum* | Herbaceous | Yes |
| *Portulaca oleracea* | Portulacaceae | *Portulaca* | Herbaceous | Yes |
| *Artemisia rutifolia* | Asteraceae | *Artemisia* | Herbaceous | Yes |
| *Dilophia salsa* | Brassicaceae | *Dilophia* | Herbaceous | Yes |
| *Carum carvi* | Apiaceae | *Carum* | Herbaceous | Yes |
| *Sonchus oleraceus* | Asteraceae | *Sonchus* | Herbaceous | No |
| *Comastoma falcatum* | Gentianaceae | *Comastoma* | Herbaceous | Yes |
| *Sonchus wightianus* | Asteraceae | *Sonchus* | Herbaceous | Yes |
| *Nitraria sphaerocarpa* | Nitrariaceae | *Nitraria* | Woody | Yes |
| *Asparagus breslerianus* | Asparagaceae | *Asparagus* | Herbaceous | Yes |
| *Centaurium pulchellum* | Gentianaceae | *Centaurium* | Herbaceous | No |
| *Galium verum* | Rubiaceae | *Galium* | Herbaceous | Yes |
| *Ajania trilobata* | Asteraceae | *Ajania* | Woody | Yes |
| *Melilotus officinalis* | Fabaceae | *Melilotus* | Herbaceous | No |
| *Gentiana prostrata var. karelinii* | Gentianaceae | *Gentiana* | Herbaceous | Yes |
| *Corydalis kashgarica* | Papaveraceae | *Corydalis* | Herbaceous | Yes |
| *Datura stramonium* | Solanaceae | *Datura* | Herbaceous | Yes |
| *Saussurea gnaphalodes* | Asteraceae | *Saussurea* | Herbaceous | Yes |
| *Stipa breviflora* | Poaceae | *Stipa* | Herbaceous | Yes |
| *Glycyrrhiza uralensis* | Fabaceae | *Glycyrrhiza* | Herbaceous | Yes |
| *Corethrodendron multijugum* | Fabaceae | *Corethrodendron* | Woody | No |
| *Myricaria squamosa* | Tamaricaceae | *Myricaria* | Woody | Yes |
| *Draba altaica* | Brassicaceae | *Draba* | Herbaceous | Yes |
| *Oxyria digyna* | Polygonaceae | *Oxyria* | Herbaceous | Yes |
| *Hordeum roshevitzii* | Poaceae | *Hordeum* | Herbaceous | Yes |
| *Androsace squarrosula* | Primulaceae | *Androsace* | Herbaceous | Yes |
| *Bassia prostrata* | Amaranthaceae | *Bassia* | Woody | Yes |
| *Orobanche cernua* | Orobanchaceae | *Orobanche* | Herbaceous | Yes |
| *Rheum reticulatum* | Polygonaceae | *Rheum* | Herbaceous | Yes |
| *Rumex patientia* | Polygonaceae | *Rumex* | Herbaceous | Yes |
| *Kalidium cuspidatum* | Amaranthaceae | *Kalidium* | Woody | No |
| *Tamarix elongata* | Tamaricaceae | *Tamarix* | Woody | Yes |
| *Leontopodium nanum* | Asteraceae | *Leontopodium* | Herbaceous | Yes |
| *Lepyrodiclis holosteoides* | Caryophyllaceae | *Lepyrodiclis* | Herbaceous | Yes |
| *Hordeum bogdanii* | Poaceae | *Hordeum* | Herbaceous | Yes |
| *Neobrachyactis roylei* | Asteraceae | *Neobrachyactis* | Herbaceous | Yes |
| *Carex deasyi* | Cyperaceae | *Carex* | Herbaceous | Yes |
| *Calamagrostis epigejos* | Poaceae | *Calamagrostis* | Herbaceous | No |
| *Juniperus semiglobosa* | Cupressaceae | *Juniperus* | Woody | Yes |
| *Ephedra regeliana* | Ephedraceae | *Ephedra* | Woody | Yes |
| *Stipa purpurea* | Poaceae | *Stipa* | Herbaceous | Yes |
| *Tamarix laxa* | Tamaricaceae | *Tamarix* | Woody | Yes |
| *Takhtajaniantha mongolica* | Asteraceae | *Takhtajaniantha* | Herbaceous | Yes |
| *Leiospora exscapa* | Brassicaceae | *Leiospora* | Herbaceous | Yes |
| *Eutrema altaicum* | Brassicaceae | *Eutrema* | Herbaceous | Yes |
| *Braya rosea* | Brassicaceae | *Braya* | Herbaceous | Yes |
| *Poa attenuata* | Poaceae | *Poa* | Herbaceous | Yes |
| *Oxytropis merkensis* | Fabaceae | *Oxytropis* | Herbaceous | Yes |
| *Stipa caucasica subsp. glareosa* | Poaceae | *Stipa* | Herbaceous | Yes |
| *Ajania tibetica* | Asteraceae | *Ajania* | Woody | Yes |
| *Salix wilhelmsiana* | Salicaceae | *Salix* | Woody | Yes |
| *Rhodiola gelida* | Crassulaceae | *Rhodiola* | Herbaceous | Yes |
| *Saussurea salsa* | Asteraceae | *Saussurea* | Herbaceous | Yes |
| *Ajania fruticulosa* | Asteraceae | *Ajania* | Woody | Yes |
| *Suaeda glauca* | Amaranthaceae | *Suaeda* | Herbaceous | No |
| *Pedicularis oederi* | Orobanchaceae | *Pedicularis* | Herbaceous | Yes |
| *Euphrasia pectinata* | Orobanchaceae | *Euphrasia* | Herbaceous | Yes |
| *Carex turkestanica* | Cyperaceae | *Carex* | Herbaceous | Yes |
| *Picea schrenkiana* | Pinaceae | *Picea* | Woody | Yes |
| *Eriocycla pelliotii* | Apiaceae | *Eriocycla* | Herbaceous | Yes |
| *Hylotelephium ewersii* | Crassulaceae | *Hylotelephium* | Herbaceous | Yes |
| *Pedicularis dolichorrhiza* | Orobanchaceae | *Pedicularis* | Herbaceous | Yes |
| *Takhtajaniantha pseudodivaricata* | Asteraceae | *Takhtajaniantha* | Herbaceous | Yes |
| *Stellaria soongorica* | Caryophyllaceae | *Stellaria* | Herbaceous | Yes |
| *Callianthemum alatavicum* | Ranunculaceae | *Callianthemum* | Herbaceous | Yes |
| *Plantago himalaica* | Plantaginaceae | *Plantago* | Herbaceous | Yes |
| *Salsola pellucida* | Amaranthaceae | *Salsola* | Herbaceous | Yes |
| *Bolboschoenus planiculmis* | Cyperaceae | *Bolboschoenus* | Herbaceous | Yes |
| *Plantago asiatica* | Plantaginaceae | *Plantago* | Herbaceous | No |
| *Gentiana pseudoaquatica* | Gentianaceae | *Gentiana* | Herbaceous | Yes |
| *Silene gonosperma* | Caryophyllaceae | *Silene* | Herbaceous | Yes |
| *Trollius dschungaricus* | Ranunculaceae | *Trollius* | Herbaceous | Yes |
| *Potentilla sericea* | Rosaceae | *Potentilla* | Herbaceous | Yes |
| *Valeriana fedtschenkoi* | Caprifoliaceae | *Valeriana* | Herbaceous | Yes |
| *Dracocephalum bipinnatum* | Lamiaceae | *Dracocephalum* | Herbaceous | Yes |
| *Asparagus angulofractus* | Asparagaceae | *Asparagus* | Herbaceous | Yes |
| *Aristida adscensionis* | Poaceae | *Aristida* | Herbaceous | Yes |
| *Tamarix karelinii* | Tamaricaceae | *Tamarix* | Woody | Yes |
| *Smelowskia tibetica* | Brassicaceae | *Smelowskia* | Herbaceous | Yes |
| *Carex melanantha* | Cyperaceae | *Carex* | Herbaceous | Yes |
| *Schoenoplectus tabernaemontani* | Cyperaceae | *Schoenoplectus* | Herbaceous | Yes |
| *Chorispora sibirica* | Brassicaceae | *Chorispora* | Herbaceous | Yes |
| *Phelipanche aegyptiaca* | Orobanchaceae | *Phelipanche* | Herbaceous | Yes |
| *Asperugo procumbens* | Boraginaceae | *Asperugo* | Herbaceous | Yes |
| *Chondrilla piptocoma* | Asteraceae | *Chondrilla* | Herbaceous | Yes |
| *Inula caspica* | Asteraceae | *Inula* | Herbaceous | Yes |
| *Odontites vulgaris* | Orobanchaceae | *Odontites* | Herbaceous | Yes |
| *Potentilla virgata* | Rosaceae | *Potentilla* | Herbaceous | Yes |
| *Sisymbrium brassiciforme* | Brassicaceae | *Sisymbrium* | Herbaceous | Yes |
| *Acantholimon diapensioides* | Plumbaginaceae | *Acantholimon* | Woody | Yes |
| *Festuca coelestis* | Poaceae | *Festuca* | Herbaceous | Yes |
| *Convolvulus tragacanthoides* | Convolvulaceae | *Convolvulus* | Woody | Yes |
| *Ephedra monosperma* | Ephedraceae | *Ephedra* | Woody | Yes |
| *Viola kunawarensis* | Violaceae | *Viola* | Herbaceous | Yes |
| *Rosa albertii* | Rosaceae | *Rosa* | Woody | Yes |
| *Stuckenia pectinata* | Potamogetonaceae | *Stuckenia* | Herbaceous | Yes |
| *Puccinellia distans* | Poaceae | *Puccinellia* | Herbaceous | Yes |
| *Ixeris polycephala* | Asteraceae | *Ixeris* | Herbaceous | Yes |
| *Gentiana leucomelaena* | Gentianaceae | *Gentiana* | Herbaceous | Yes |
| *Aconitum rotundifolium* | Ranunculaceae | *Aconitum* | Herbaceous | No |
| *Blysmus compressus* | Cyperaceae | *Blysmus* | Herbaceous | Yes |
| *Iljinia regelii* | Amaranthaceae | *Iljinia* | Woody | Yes |
| *Salsola junatovii* | Amaranthaceae | *Salsola* | Woody | Yes |
| *Zygophyllum obliquum* | Zygophyllaceae | *Zygophyllum* | Herbaceous | Yes |
| *Poa litwinowiana* | Poaceae | *Poa* | Herbaceous | Yes |
| *Acantholimon hedinii* | Plumbaginaceae | *Acantholimon* | Woody | Yes |
| *Persicaria amphibia* | Polygonaceae | *Persicaria* | Herbaceous | No |
| *Gueldenstaedtia verna* | Fabaceae | *Gueldenstaedtia* | Herbaceous | Yes |
| *Pedicularis rhinanthoides* | Orobanchaceae | *Pedicularis* | Herbaceous | Yes |
| *Clematis sibirica* | Ranunculaceae | *Clematis* | Woody | Yes |
| *Rosa fedtschenkoana* | Rosaceae | *Rosa* | Woody | Yes |
| *Potentilla supina* | Rosaceae | *Potentilla* | Herbaceous | No |
| *Zygophyllum macropodum* | Zygophyllaceae | *Zygophyllum* | Herbaceous | Yes |
| *Elaeagnus oxycarpa* | Elaeagnaceae | *Elaeagnus* | Woody | Yes |
| *Bolboschoenus maritimus* | Cyperaceae | *Bolboschoenus* | Herbaceous | Yes |
| *Carex pseudofoetida* | Cyperaceae | *Carex* | Herbaceous | Yes |
| *Aeluropus pungens* | Poaceae | *Aeluropus* | Herbaceous | Yes |
| *Convolvulus fruticosus* | Convolvulaceae | *Convolvulus* | Woody | Yes |
| *Artemisia annua* | Asteraceae | *Artemisia* | Herbaceous | Yes |
| *Halerpestes cymbalaria* | Ranunculaceae | *Halerpestes* | Herbaceous | Yes |
| *Sibbaldia tetrandra* | Rosaceae | *Sibbaldia* | Herbaceous | Yes |
| *Hibiscus trionum* | Malvaceae | *Hibiscus* | Herbaceous | Yes |
| *Lepidium ruderale* | Brassicaceae | *Lepidium* | Herbaceous | No |
| *Cotoneaster soongoricus* | Rosaceae | *Cotoneaster* | Woody | Yes |
| *Nitraria roborowskii* | Nitrariaceae | *Nitraria* | Woody | Yes |
| *Sonchus uliginosus* | Asteraceae | *Sonchus* | Herbaceous | No |
| *Strigosella hispida* | Brassicaceae | *Strigosella* | Herbaceous | Yes |
| *Apocynum venetum* | Apocynaceae | *Apocynum* | Woody | Yes |
| *Oxytropis densa* | Fabaceae | *Oxytropis* | Herbaceous | Yes |
| *Lagotis decumbens* | Plantaginaceae | *Lagotis* | Herbaceous | Yes |
| *Blitum virgatum* | Amaranthaceae | *Blitum* | Herbaceous | Yes |
| *Reaumuria kaschgarica* | Tamaricaceae | *Reaumuria* | Woody | Yes |
| *Sonchus brachyotus* | Asteraceae | *Sonchus* | Herbaceous | Yes |
| *Cynodon dactylon* | Poaceae | *Cynodon* | Herbaceous | Yes |
| *Silene graminifolia* | Caryophyllaceae | *Silene* | Herbaceous | Yes |
| *Scrophularia incisa* | Scrophulariaceae | *Scrophularia* | Herbaceous | Yes |
| *Plantago cornuti* | Plantaginaceae | *Plantago* | Herbaceous | Yes |
| *Orostachys thyrsiflora* | Crassulaceae | *Orostachys* | Herbaceous | Yes |
| *Rhodiola coccinea* | Crassulaceae | *Rhodiola* | Herbaceous | Yes |
| *Carex alatauensis* | Cyperaceae | *Carex* | Herbaceous | Yes |
| *Blysmus sinocompressus* | Cyperaceae | *Blysmus* | Herbaceous | Yes |
| *Rosa platyacantha* | Rosaceae | *Rosa* | Woody | Yes |
| *Acantholimon tianschanicum* | Plumbaginaceae | *Acantholimon* | Woody | Yes |
| *Ribes meyeri* | Grossulariaceae | *Ribes* | Woody | Yes |
| *Caragana turfanensis* | Fabaceae | *Caragana* | Woody | Yes |
| *Gagea serotina* | Liliaceae | *Gagea* | Herbaceous | Yes |
| *Braya tibetica* | Brassicaceae | *Braya* | Herbaceous | Yes |
| *Artemisia lavandulifolia* | Asteraceae | *Artemisia* | Herbaceous | Yes |
| *Corispermum lehmannianum* | Amaranthaceae | *Corispermum* | Herbaceous | Yes |
| *Geranium pseudosibiricum* | Geraniaceae | *Geranium* | Herbaceous | Yes |
| *Salix capusii* | Salicaceae | *Salix* | Woody | Yes |
| *Ligularia alpigena* | Asteraceae | *Ligularia* | Herbaceous | Yes |
| *Sorbus tianschanica* | Rosaceae | *Sorbus* | Woody | Yes |
| *Plantago salsa* | Plantaginaceae | *Plantago* | Herbaceous | Yes |
| *Potentilla multifida var. ornithopoda* | Rosaceae | *Potentilla* | Herbaceous | Yes |
| *Persicaria maculosa* | Polygonaceae | *Persicaria* | Herbaceous | Yes |
| *Berberis atrocarpa* | Berberidaceae | *Berberis* | Woody | No |
| *Scirpus wichurae* | Cyperaceae | *Scirpus* | Herbaceous | Yes |
| *Agriophyllum pungens* | Amaranthaceae | *Agriophyllum* | Herbaceous | Yes |
| *Leontopodium fedtschenkoanum* | Asteraceae | *Leontopodium* | Herbaceous | Yes |
| *Androsace ovczinnikovii* | Primulaceae | *Androsace* | Herbaceous | Yes |
| *Lotus tenuis* | Fabaceae | *Lotus* | Herbaceous | Yes |
| *Carex orbicularis* | Cyperaceae | *Carex* | Herbaceous | Yes |
| *Dactylorhiza hatagirea* | Orchidaceae | *Dactylorhiza* | Herbaceous | Yes |
| *Poa pratensis* | Poaceae | *Poa* | Herbaceous | Yes |
| *Agrostis gigantea* | Poaceae | *Agrostis* | Herbaceous | Yes |
| *Artemisia selengensis* | Asteraceae | *Artemisia* | Herbaceous | No |
| *Carex moorcroftii* | Cyperaceae | *Carex* | Herbaceous | Yes |
| *Equisetum arvense* | Equisetaceae | *Equisetum* | Herbaceous | Yes |
| *Typha laxmannii* | Typhaceae | *Typha* | Herbaceous | Yes |
| *Oreosalsola abrotanoides* | Amaranthaceae | *Oreosalsola* | Woody | Yes |
| *Sonchus transcaspicus* | Asteraceae | *Sonchus* | Herbaceous | No |
| *Rubia tinctorum* | Rubiaceae | *Rubia* | Herbaceous | Yes |
| *Hippuris vulgaris* | Plantaginaceae | *Hippuris* | Herbaceous | Yes |
| *Suaeda stellatiflora* | Amaranthaceae | *Suaeda* | Herbaceous | Yes |
| *Eruca vesicaria subsp. sativa* | Brassicaceae | *Eruca* | Herbaceous | Yes |
| *Zygophyllum loczyi* | Zygophyllaceae | *Zygophyllum* | Herbaceous | Yes |
| *Ligusticum mucronatum* | Apiaceae | *Ligusticum* | Herbaceous | Yes |
| *Peganum multisectum* | Nitrariaceae | *Peganum* | Herbaceous | Yes |
| *Primula algida* | Primulaceae | *Primula* | Herbaceous | Yes |
| *Caragana sinica* | Fabaceae | *Caragana* | Woody | Yes |
| *Rhodiola kashgarica* | Crassulaceae | *Rhodiola* | Herbaceous | Yes |
| *Chenopodium karoi* | Amaranthaceae | *Chenopodium* | Herbaceous | Yes |
| *Astragalus polycladus* | Fabaceae | *Astragalus* | Herbaceous | Yes |
| *Thermopsis alpina* | Fabaceae | *Thermopsis* | Herbaceous | Yes |
| *Clematis florida* | Ranunculaceae | *Clematis* | Herbaceous | Yes |
| *Polygonum plebeium* | Polygonaceae | *Polygonum* | Herbaceous | Yes |
| *Zygophyllum brachypterum* | Zygophyllaceae | *Zygophyllum* | Herbaceous | Yes |
| *Chenopodium iljinii* | Amaranthaceae | *Chenopodium* | Herbaceous | Yes |
| *Gentianopsis stricta* | Gentianaceae | *Gentianopsis* | Herbaceous | Yes |
| *Avena fatua* | Poaceae | *Avena* | Herbaceous | Yes |
| *Polygonum argyrocoleon* | Polygonaceae | *Polygonum* | Herbaceous | Yes |
| *Cortusa matthioli* | Primulaceae | *Cortusa* | Herbaceous | No |
| *Setaria pumila* | Poaceae | *Setaria* | Herbaceous | Yes |
| *Galium spurium* | Rubiaceae | *Galium* | Herbaceous | No |
| *Veronica biloba* | Plantaginaceae | *Veronica* | Herbaceous | No |
| *Leymus paboanus* | Poaceae | *Leymus* | Herbaceous | Yes |
| *Phelipanche caesia* | Orobanchaceae | *Phelipanche* | Herbaceous | Yes |
| *Salix caesia* | Salicaceae | *Salix* | Woody | No |
| *Ammopiptanthus nanus* | Fabaceae | *Ammopiptanthus* | Woody | No |
| *Populus afghanica* | Salicaceae | *Populus* | Woody | Yes |
| *Rosularia platyphylla* | Crassulaceae | *Rosularia* | Herbaceous | Yes |
| *Cirsium sairamense* | Asteraceae | *Cirsium* | Herbaceous | Yes |
| *Thalictrum foetidum* | Ranunculaceae | *Thalictrum* | Herbaceous | Yes |
| *Krascheninnikovia ewersmannia* | Amaranthaceae | *Krascheninnikovia* | Woody | Yes |
| *Pulsatilla campanella* | Ranunculaceae | *Pulsatilla* | Herbaceous | Yes |
| *Iris ruthenica* | Iridaceae | *Iris* | Herbaceous | No |
| *Thalictrum alpinum* | Ranunculaceae | *Thalictrum* | Herbaceous | Yes |
| *Clematis songorica var. aspleniifolia* | Ranunculaceae | *Clematis* | Herbaceous | Yes |
| *Chorispora tenella* | Brassicaceae | *Chorispora* | Herbaceous | Yes |
| *Saussurea ovata* | Asteraceae | *Saussurea* | Herbaceous | Yes |
| *Gypsophila cephalotes* | Caryophyllaceae | *Gypsophila* | Herbaceous | No |
| *Geranium saxatile* | Geraniaceae | *Geranium* | Herbaceous | Yes |
| *Lomatogonium brachyantherum* | Gentianaceae | *Lomatogonium* | Herbaceous | Yes |
| *Clematis glauca* | Ranunculaceae | *Clematis* | Herbaceous | No |
| *Plantago griffithii* | Plantaginaceae | *Plantago* | Herbaceous | Yes |
| *Solanum villosum* | Solanaceae | *Solanum* | Herbaceous | Yes |
| *Lonicera webbiana* | Caprifoliaceae | *Lonicera* | Woody | Yes |
| *Corydalis adiantifolia* | Papaveraceae | *Corydalis* | Herbaceous | Yes |
| *Potentilla chinensis* | Rosaceae | *Potentilla* | Herbaceous | Yes |
| *Salix alatavica* | Salicaceae | *Salix* | Woody | No |
| *Trichophorum pumilum* | Cyperaceae | *Trichophorum* | Herbaceous | Yes |
| *Leymus ovatus* | Poaceae | *Leymus* | Herbaceous | Yes |
| *Iris halophila var. sogdiana* | Iridaceae | *Iris* | Herbaceous | No |
| *Allium przewalskianum* | Amaryllidaceae | *Allium* | Herbaceous | Yes |
| *Typha angustifolia* | Typhaceae | *Typha* | Herbaceous | Yes |
| *Echinops nanus* | Asteraceae | *Echinops* | Herbaceous | No |
| *Acantholimon popovii* | Plumbaginaceae | *Acantholimon* | Woody | No |
| *Knorringia sibirica* | Polygonaceae | *Knorringia* | Herbaceous | Yes |
| *Myrtama elegans* | Tamaricaceae | *Myrtama* | Woody | Yes |
| *Saussurea subulata* | Asteraceae | *Saussurea* | Herbaceous | Yes |
| *Lagochilus grandiflorus* | Lamiaceae | *Lagochilus* | Herbaceous | Yes |
| *Primula macrophylla* | Primulaceae | *Primula* | Herbaceous | No |
| *Elymus burchan-buddae* | Poaceae | *Elymus* | Herbaceous | Yes |
| *Braya scharnhorstii* | Brassicaceae | *Braya* | Herbaceous | Yes |
| *Thalictrum flavum* | Ranunculaceae | *Thalictrum* | Herbaceous | Yes |
| *Allium schoenoprasoides* | Amaryllidaceae | *Allium* | Herbaceous | No |
| *Artemisia frigida* | Asteraceae | *Artemisia* | Herbaceous | Yes |
| *Silene conoidea* | Caryophyllaceae | *Silene* | Herbaceous | Yes |
| *Ephedra equisetina* | Ephedraceae | *Ephedra* | Woody | Yes |
| *Elymus schrenkianus* | Poaceae | *Elymus* | Herbaceous | Yes |
| *Stipa tianschanica* | Poaceae | *Stipa* | Herbaceous | No |
| *Carex enervis* | Cyperaceae | *Carex* | Herbaceous | Yes |
| *Carex atrofusca* | Cyperaceae | *Carex* | Herbaceous | Yes |
| *Artemisia leucophylla* | Asteraceae | *Artemisia* | Herbaceous | Yes |
| *Galium boreale* | Rubiaceae | *Galium* | Herbaceous | No |
| *Ligularia macrophylla* | Asteraceae | *Ligularia* | Herbaceous | Yes |
| *Ajania scharnhorstii* | Asteraceae | *Ajania* | Woody | Yes |
| *Gentianella azurea* | Gentianaceae | *Gentianella* | Herbaceous | Yes |
| *Bidens tripartita* | Asteraceae | *Bidens* | Herbaceous | Yes |
| *Oxytropis falcata* | Fabaceae | *Oxytropis* | Herbaceous | Yes |
| *Hippophae rhamnoides subsp. mongolica* | Elaeagnaceae | *Hippophae* | Woody | Yes |
| *Nitraria pamirica* | Nitrariaceae | *Nitraria* | Woody | Yes |
| *Rhodiola pamiroalaica* | Crassulaceae | *Rhodiola* | Herbaceous | Yes |
| *Arnebia euchroma* | Boraginaceae | *Arnebia* | Herbaceous | Yes |
| *Allium petraeum* | Amaryllidaceae | *Allium* | Herbaceous | No |
| *Senecio thianschanicus* | Asteraceae | *Senecio* | Herbaceous | Yes |
| *Iris halophila* | Iridaceae | *Iris* | Herbaceous | Yes |
| *Carex duriuscula subsp. stenophylloides* | Cyperaceae | *Carex* | Herbaceous | No |
| *Typha minima* | Typhaceae | *Typha* | Herbaceous | Yes |
| *Cicer microphyllum* | Fabaceae | *Cicer* | Herbaceous | Yes |
| *Suaeda salsa* | Amaranthaceae | *Suaeda* | Herbaceous | Yes |
| *Astragalus kuschakewiczii* | Fabaceae | *Astragalus* | Herbaceous | No |
| *Primula pamirica* | Primulaceae | *Primula* | Herbaceous | Yes |
| *Saussurea thomsonii* | Asteraceae | *Saussurea* | Herbaceous | Yes |
| *Scutellaria oligodonta* | Lamiaceae | *Scutellaria* | Woody | No |
| *Allium caricoides* | Amaryllidaceae | *Allium* | Herbaceous | Yes |
| *Tamarix leptostachya* | Tamaricaceae | *Tamarix* | Woody | Yes |
| *Leymus angustus* | Poaceae | *Leymus* | Herbaceous | Yes |
| *Allium hymenorhizum* | Amaryllidaceae | *Allium* | Herbaceous | No |
| *Cirsium esculentum* | Asteraceae | *Cirsium* | Herbaceous | Yes |
| *Allium carolinianum* | Amaryllidaceae | *Allium* | Herbaceous | Yes |
| *Oxytropis humifusa* | Fabaceae | *Oxytropis* | Herbaceous | Yes |
| *Schoenoplectus triqueter* | Cyperaceae | *Schoenoplectus* | Herbaceous | Yes |
| *Phlomoides oreophila* | Lamiaceae | *Phlomoides* | Herbaceous | Yes |
| *Klasea procumbens* | Asteraceae | *Klasea* | Herbaceous | Yes |
| *Dracocephalum imberbe* | Lamiaceae | *Dracocephalum* | Herbaceous | Yes |
| *Thlaspi arvense* | Brassicaceae | *Thlaspi* | Herbaceous | Yes |
| *Dysphania botrys* | Amaranthaceae | *Dysphania* | Herbaceous | Yes |
| *Gentiana walujewii* | Gentianaceae | *Gentiana* | Herbaceous | No |
| *Thalictrum minus* | Ranunculaceae | *Thalictrum* | Herbaceous | Yes |
| *Festuca ovina* | Poaceae | *Festuca* | Herbaceous | Yes |
| *Rumex longifolius* | Polygonaceae | *Rumex* | Herbaceous | No |
| *Ferula assa-foetida* | Apiaceae | *Ferula* | Herbaceous | No |
| *Artemisia vulgaris* | Asteraceae | *Artemisia* | Herbaceous | Yes |
| *Saussurea glacialis* | Asteraceae | *Saussurea* | Herbaceous | Yes |
| *Psychrogeton poncinsii* | Asteraceae | *Psychrogeton* | Herbaceous | No |
| *Lonicera semenovii* | Caprifoliaceae | *Lonicera* | Woody | No |
| *Caragana dasyphylla* | Fabaceae | *Caragana* | Woody | Yes |
| *Gentiana dahurica* | Gentianaceae | *Gentiana* | Herbaceous | No |
| *Saxifraga pulvinaria* | Saxifragaceae | *Saxifraga* | Herbaceous | Yes |
| *Dasiphora dryadanthoides* | Rosaceae | *Dasiphora* | Woody | Yes |
| *Astragalus alpinus* | Fabaceae | *Astragalus* | Herbaceous | No |
| *Cirsium japonicum* | Asteraceae | *Cirsium* | Herbaceous | Yes |
| *Bolboschoenus yagara* | Cyperaceae | *Bolboschoenus* | Herbaceous | Yes |
| *Elsholtzia densa* | Lamiaceae | *Elsholtzia* | Herbaceous | Yes |
| *Fraxinus sogdiana* | Oleaceae | *Fraxinus* | Woody | Yes |
| *Stipa sareptana var. krylovii* | Poaceae | *Stipa* | Herbaceous | Yes |
| *Knorringia sibirica subsp. thomsonii* | Polygonaceae | *Knorringia* | Herbaceous | Yes |
| *Epilobium minutiflorum* | Onagraceae | *Epilobium* | Herbaceous | Yes |
| *Asterothamnus centraliasiaticus* | Asteraceae | *Asterothamnus* | Woody | Yes |
| *Lonicera hispida* | Caprifoliaceae | *Lonicera* | Woody | No |
| *Dasiphora fruticosa* | Rosaceae | *Dasiphora* | Woody | Yes |
| *Nitraria schoberi* | Nitrariaceae | *Nitraria* | Woody | Yes |
| *Delphinium lacostei* | Ranunculaceae | *Delphinium* | Herbaceous | Yes |
| *Juncus bufonius* | Juncaceae | *Juncus* | Herbaceous | Yes |
| *Patrinia intermedia* | Caprifoliaceae | *Patrinia* | Herbaceous | No |
| *Amaranthus blitum* | Amaranthaceae | *Amaranthus* | Herbaceous | Yes |
| *Androsace septentrionalis* | Primulaceae | *Androsace* | Herbaceous | Yes |
| *Ephedra sinica* | Ephedraceae | *Ephedra* | Woody | Yes |
| *Potentilla conferta* | Rosaceae | *Potentilla* | Herbaceous | Yes |
| *Calamagrostis hedinii* | Poaceae | *Calamagrostis* | Herbaceous | No |
| *Caragana pleiophylla* | Fabaceae | *Caragana* | Woody | Yes |
| *Erigeron acris* | Asteraceae | *Erigeron* | Herbaceous | Yes |
| *Poa calliopsis* | Poaceae | *Poa* | Herbaceous | Yes |
| *Taraxacum monochlamydeum* | Asteraceae | *Taraxacum* | Herbaceous | Yes |
| *Capsella bursa-pastoris* | Brassicaceae | *Capsella* | Herbaceous | Yes |
| *Kengyilia alatavica* | Poaceae | *Kengyilia* | Herbaceous | Yes |
| *Populus pamirica* | Salicaceae | *Populus* | Woody | No |
| *Puccinellia himalaica* | Poaceae | *Puccinellia* | Herbaceous | Yes |
| *Artemisia stechmanniana* | Asteraceae | *Artemisia* | Herbaceous | Yes |
| *Salsola sinkiangensis* | Amaranthaceae | *Salsola* | Herbaceous | Yes |
| *Rhodiola heterodonta* | Crassulaceae | *Rhodiola* | Herbaceous | No |
| *Rhodiola recticaulis* | Crassulaceae | *Rhodiola* | Herbaceous | Yes |
| *Silene songarica* | Caryophyllaceae | *Silene* | Herbaceous | Yes |
| *Campanula glomerata* | Campanulaceae | *Campanula* | Herbaceous | Yes |
| *Chorispora bungeana* | Brassicaceae | *Chorispora* | Herbaceous | No |
| *Taraxacum luridum* | Asteraceae | *Taraxacum* | Herbaceous | Yes |
| *Artemisia mongolica* | Asteraceae | *Artemisia* | Herbaceous | Yes |
| *Cuscuta europaea* | Convolvulaceae | *Cuscuta* | Herbaceous | Yes |
| *Eleocharis quinqueflora* | Cyperaceae | *Eleocharis* | Herbaceous | Yes |
| *Poa pratensis subsp. angustifolia* | Poaceae | *Poa* | Herbaceous | Yes |
| *Chorispora macropoda* | Brassicaceae | *Chorispora* | Herbaceous | No |
| *Chenopodium ficifolium* | Amaranthaceae | *Chenopodium* | Herbaceous | No |
| *Smelowskia sisymbrioides* | Brassicaceae | *Smelowskia* | Herbaceous | No |
| *Rumex crispus* | Polygonaceae | *Rumex* | Herbaceous | Yes |
| *Artemisia scoparia* | Asteraceae | *Artemisia* | Herbaceous | Yes |
| *Digitaria violascens* | Poaceae | *Digitaria* | Herbaceous | Yes |
| *Caragana leucophloea* | Fabaceae | *Caragana* | Woody | Yes |
| *Rosa webbiana* | Rosaceae | *Rosa* | Woody | Yes |
| *Atriplex tatarica* | Amaranthaceae | *Atriplex* | Herbaceous | Yes |
| *Stipa orientalis* | Poaceae | *Stipa* | Herbaceous | Yes |
| *Lappula brachycentra* | Boraginaceae | *Lappula* | Herbaceous | Yes |
| *Hedysarum ferganense var. poncinsii* | Fabaceae | *Hedysarum* | Herbaceous | No |
| *Parnassia palustris* | Celastraceae | *Parnassia* | Herbaceous | Yes |
| *Hordeum brevisubulatum subsp. nevskianum* | Poaceae | *Hordeum* | Herbaceous | No |
| *Oxytropis gorbunovii* | Fabaceae | *Oxytropis* | Herbaceous | Yes |
| *Suaeda heterophylla* | Amaranthaceae | *Suaeda* | Herbaceous | Yes |
| *Viola biflora* | Violaceae | *Viola* | Herbaceous | Yes |
| *Echinochloa crus-galli var. mitis* | Poaceae | *Echinochloa* | Herbaceous | Yes |
| *Salix rosmarinifolia* | Salicaceae | *Salix* | Herbaceous | No |
| *Thermopsis turkestanica* | Fabaceae | *Thermopsis* | Herbaceous | Yes |
| *Cicerbita azurea* | Asteraceae | *Cicerbita* | Herbaceous | No |
| *Rosularia alpestris* | Crassulaceae | *Rosularia* | Herbaceous | No |
| *Lonicera humilis* | Caprifoliaceae | *Lonicera* | Woody | Yes |
| *Calamagrostis macrolepis* | Poaceae | *Calamagrostis* | Herbaceous | Yes |
| *Potentilla multicaulis* | Rosaceae | *Potentilla* | Herbaceous | No |
| *Carex microglochin* | Cyperaceae | *Carex* | Herbaceous | Yes |
| *Androsace nortonii* | Primulaceae | *Androsace* | Herbaceous | No |
| *Lagochilus kaschgaricus* | Lamiaceae | *Lagochilus* | Herbaceous | No |
| *Oxytropis poncinsii* | Fabaceae | *Oxytropis* | Herbaceous | Yes |
| *Tamarix tarimensis* | Tamaricaceae | *Tamarix* | Woody | No |
| *Colchicum kesselringii* | Colchicaceae | *Colchicum* | Herbaceous | No |
| *Solms-laubachia himalayensis* | Brassicaceae | *Solms-laubachia* | Herbaceous | No |
| *Medicago falcata* | Fabaceae | *Medicago* | Herbaceous | Yes |
| *Cerastium arvense* | Caryophyllaceae | *Cerastium* | Herbaceous | No |
| *Stevenia canescens* | Brassicaceae | *Stevenia* | Woody | Yes |
| *Euphrasia hirtella* | Orobanchaceae | *Euphrasia* | Herbaceous | No |
| *Smelowskia annua* | Brassicaceae | *Smelowskia* | Herbaceous | No |
| *Allium kaschianum* | Amaryllidaceae | *Allium* | Herbaceous | No |
| *Phlomoides pratensis* | Lamiaceae | *Phlomoides* | Herbaceous | Yes |
| *Carex sargentiana* | Cyperaceae | *Carex* | Herbaceous | No |
| *Pycnoplinthus uniflora* | Brassicaceae | *Pycnoplinthus* | Herbaceous | Yes |
| *Androsace maxima* | Primulaceae | *Androsace* | Herbaceous | Yes |
| *Lappula squarrosa* | Boraginaceae | *Lappula* | Herbaceous | Yes |
| *Chondrilla brevirostris* | Asteraceae | *Chondrilla* | Herbaceous | No |
| *Hordeum brevisubulatum* | Poaceae | *Hordeum* | Herbaceous | Yes |
| *Poa alpina* | Poaceae | *Poa* | Herbaceous | No |
| *Cyperus fuscus* | Cyperaceae | *Cyperus* | Herbaceous | Yes |
| *Lappula myosotis* | Boraginaceae | *Lappula* | Herbaceous | Yes |
| *Erigeron pseudoseravschanicus* | Asteraceae | *Erigeron* | Herbaceous | Yes |
| *Tulipa dasystemon* | Liliaceae | *Tulipa* | Herbaceous | No |
| *Halerpestes tricuspis* | Ranunculaceae | *Halerpestes* | Herbaceous | No |
| *Saxifraga sinomontana* | Saxifragaceae | *Saxifraga* | Herbaceous | No |
| *Taraxacum scariosum* | Asteraceae | *Taraxacum* | Herbaceous | Yes |
| *Draba nemorosa* | Brassicaceae | *Draba* | Herbaceous | Yes |
| *Crepidiastrum diversifolium* | Asteraceae | *Crepidiastrum* | Herbaceous | No |
| *Suaeda rigida* | Amaranthaceae | *Suaeda* | Herbaceous | Yes |
| *Elymus dahuricus var. cylindricus* | Poaceae | *Elymus* | Herbaceous | Yes |
| *Corydalis stricta* | Papaveraceae | *Corydalis* | Herbaceous | Yes |
| *Neopallasia pectinata* | Asteraceae | *Neopallasia* | Herbaceous | Yes |
| *Festuca rubra* | Poaceae | *Festuca* | Herbaceous | Yes |
| *Artemisia gmelinii* | Asteraceae | *Artemisia* | Woody | Yes |
| *Bothriochloa ischaemum* | Poaceae | *Bothriochloa* | Herbaceous | Yes |
| *Allardia tridactylites* | Asteraceae | *Allardia* | Herbaceous | Yes |
| *Smelowskia bifurcata* | Brassicaceae | *Smelowskia* | Herbaceous | No |
| *Acantholimon kokandense* | Plumbaginaceae | *Acantholimon* | Woody | No |
| *Populus talassica* | Salicaceae | *Populus* | Woody | No |
| *Arctium lappa* | Asteraceae | *Arctium* | Herbaceous | Yes |
| *Suaeda arcuata* | Amaranthaceae | *Suaeda* | Herbaceous | No |
| *Myricaria pulcherrima* | Tamaricaceae | *Myricaria* | Woody | Yes |
| *Artemisia rupestris* | Asteraceae | *Artemisia* | Herbaceous | Yes |
| *Eleocharis palustris* | Cyperaceae | *Eleocharis* | Herbaceous | No |
| *Pedicularis semenowii* | Orobanchaceae | *Pedicularis* | Herbaceous | No |
| *Koenigia songarica* | Polygonaceae | *Koenigia* | Herbaceous | No |
| *Astragalus alatavicus* | Fabaceae | *Astragalus* | Herbaceous | No |
| *Pennisetum flaccidum* | Poaceae | *Pennisetum* | Herbaceous | Yes |
| *Polypogon fugax* | Poaceae | *Polypogon* | Herbaceous | Yes |
| *Saussurea thoroldii* | Asteraceae | *Saussurea* | Herbaceous | Yes |
| *Leymus secalinus var. pubescens* | Poaceae | *Leymus* | Herbaceous | Yes |
| *Phlomoides alpina* | Lamiaceae | *Phlomoides* | Herbaceous | No |
| *Pedicularis physocalyx* | Orobanchaceae | *Pedicularis* | Herbaceous | Yes |
| *Festuca kryloviana* | Poaceae | *Festuca* | Herbaceous | Yes |
| *Pedicularis abrotanifolia* | Orobanchaceae | *Pedicularis* | Herbaceous | Yes |
| *Puccinellia hauptiana* | Poaceae | *Puccinellia* | Herbaceous | Yes |
| *Grubovia melanoptera* | Amaranthaceae | *Grubovia* | Herbaceous | Yes |
| *Geranium wilfordii* | Geraniaceae | *Geranium* | Herbaceous | Yes |
| *Taraxacum minutilobum* | Asteraceae | *Taraxacum* | Herbaceous | No |
| *Astragalus pamirensis* | Fabaceae | *Astragalus* | Herbaceous | Yes |
| *Saussurea larionowii* | Asteraceae | *Saussurea* | Herbaceous | No |
| *Draba oreades* | Brassicaceae | *Draba* | Herbaceous | Yes |
| *Artemisia demissa* | Asteraceae | *Artemisia* | Herbaceous | Yes |
| *Erigeron canadensis* | Asteraceae | *Erigeron* | Herbaceous | Yes |
| *Ajania fastigiata* | Asteraceae | *Ajania* | Herbaceous | Yes |
| *Inula rhizocephala* | Asteraceae | *Inula* | Herbaceous | No |
| *Potentilla multifida var. minor* | Rosaceae | *Potentilla* | Herbaceous | No |
| *Lotus corniculatus* | Fabaceae | *Lotus* | Herbaceous | Yes |
| *Schismus arabicus* | Poaceae | *Schismus* | Herbaceous | Yes |
| *Saussurea lacostei* | Asteraceae | *Saussurea* | Herbaceous | Yes |
| *Campanula glomerata subsp. speciosa* | Campanulaceae | *Campanula* | Herbaceous | No |
| *Eritrichium latifolium* | Boraginaceae | *Eritrichium* | Herbaceous | No |
| *Elymus sibiricus* | Poaceae | *Elymus* | Herbaceous | Yes |
| *Thylacospermum caespitosum* | Caryophyllaceae | *Thylacospermum* | Herbaceous | No |
| *Suaeda prostrata* | Amaranthaceae | *Suaeda* | Herbaceous | Yes |
| *Dracocephalum integrifolium* | Lamiaceae | *Dracocephalum* | Herbaceous | No |
| *Lepidium draba* | Brassicaceae | *Lepidium* | Herbaceous | Yes |
| *Echinochloa oryzoides* | Poaceae | *Echinochloa* | Herbaceous | Yes |
| *Persicaria lapathifolia* | Polygonaceae | *Persicaria* | Herbaceous | Yes |
| *Oxytropis tianschanica* | Fabaceae | *Oxytropis* | Herbaceous | No |
| *Juncus triglumis* | Juncaceae | *Juncus* | Herbaceous | Yes |
| *Dilophia ebracteata* | Brassicaceae | *Dilophia* | Herbaceous | Yes |
| *Bromus inermis* | Poaceae | *Bromus* | Herbaceous | No |
| *Agrostis stolonifera* | Poaceae | *Agrostis* | Herbaceous | Yes |
| *Gentianopsis barbata var. stenocalyx* | Gentianaceae | *Gentianopsis* | Herbaceous | No |
| *Valeriana officinalis* | Caprifoliaceae | *Valeriana* | Herbaceous | No |
| *Inula japonica* | Asteraceae | *Inula* | Herbaceous | Yes |
| *Malva verticillata* | Malvaceae | *Malva* | Herbaceous | Yes |
| *Sporobolus aculeatus* | Poaceae | *Sporobolus* | Herbaceous | Yes |
| *Nepeta longibracteata* | Lamiaceae | *Nepeta* | Herbaceous | No |
| *Echinochloa caudata* | Poaceae | *Echinochloa* | Herbaceous | Yes |
| *Draba lanceolata* | Brassicaceae | *Draba* | Herbaceous | No |
| *Aster semiprostratus* | Asteraceae | *Aster* | Herbaceous | Yes |
| *Senecio dubitabilis* | Asteraceae | *Senecio* | Herbaceous | No |
| *Erigeron aurantiacus* | Asteraceae | *Erigeron* | Herbaceous | Yes |
| *Suaeda acuminata* | Amaranthaceae | *Suaeda* | Herbaceous | Yes |
| *Braya parvia* | Brassicaceae | *Braya* | Herbaceous | No |
| *Dracocephalum nodulosum* | Lamiaceae | *Dracocephalum* | Herbaceous | No |
| *Leymus multicaulis* | Poaceae | *Leymus* | Herbaceous | Yes |
| *Limonium lacostei* | Plumbaginaceae | *Limonium* | Woody | Yes |
| *Saussurea andryaloides* | Asteraceae | *Saussurea* | Herbaceous | Yes |
| *Libanotis schrenkiana* | Apiaceae | *Libanotis* | Herbaceous | No |
| *Chamerion latifolium* | Onagraceae | *Chamerion* | Herbaceous | Yes |
| *Gentiana squarrosa* | Gentianaceae | *Gentiana* | Herbaceous | Yes |
| *Phlomoides speciosa* | Lamiaceae | *Phlomoides* | Herbaceous | No |
| *Corispermum mongolicum* | Amaranthaceae | *Corispermum* | Herbaceous | Yes |
| *Swertia marginata* | Gentianaceae | *Swertia* | Herbaceous | Yes |
| *Ziziphora pamiroalaica* | Lamiaceae | *Ziziphora* | Woody | No |
| *Inula britannica* | Asteraceae | *Inula* | Herbaceous | Yes |
| *Thermopsis lanceolata* | Fabaceae | *Thermopsis* | Herbaceous | No |
| *Bromus japonicus* | Poaceae | *Bromus* | Herbaceous | Yes |
| *Crucihimalaya mollissima* | Brassicaceae | *Crucihimalaya* | Herbaceous | No |
| *Bupleurum triradiatum* | Apiaceae | *Bupleurum* | Herbaceous | No |
| *Echinops gmelinii* | Asteraceae | *Echinops* | Herbaceous | Yes |
| *Poa lipskyi* | Poaceae | *Poa* | Herbaceous | Yes |
| *Alyssum desertorum* | Brassicaceae | *Alyssum* | Herbaceous | No |
| *Astragalus chomutowii* | Fabaceae | *Astragalus* | Herbaceous | Yes |
| *Centaurium pulchellum var. altaicum* | Gentianaceae | *Centaurium* | Herbaceous | No |
| *Limonium sinense* | Plumbaginaceae | *Limonium* | Herbaceous | No |
| *Juniperus sabina* | Cupressaceae | *Juniperus* | Woody | Yes |
| *Cousinia caespitosa* | Asteraceae | *Cousinia* | Herbaceous | No |
| *Olgaea roborowskyi* | Asteraceae | *Olgaea* | Herbaceous | No |
| *Vicia sativa* | Fabaceae | *Vicia* | Herbaceous | Yes |
| *Seriphidium compactum* | Asteraceae | *Seriphidium* | Herbaceous | No |
| *Kalidium caspicum* | Amaranthaceae | *Kalidium* | Woody | Yes |
| *Dichodon cerastoides* | Caryophyllaceae | *Dichodon* | Herbaceous | No |
| *Astragalus densiflorus* | Fabaceae | *Astragalus* | Herbaceous | Yes |
| *Cuscuta australis* | Convolvulaceae | *Cuscuta* | Herbaceous | Yes |
| *Eleocharis yokoscensis* | Cyperaceae | *Eleocharis* | Herbaceous | No |
| *Dracocephalum origanoides* | Lamiaceae | *Dracocephalum* | Herbaceous | No |
| *Saxifraga hirculus* | Saxifragaceae | *Saxifraga* | Herbaceous | Yes |
| *Myosotis caespitosa* | Boraginaceae | *Myosotis* | Herbaceous | No |
| *Persicaria hydropiper* | Polygonaceae | *Persicaria* | Herbaceous | No |
| *Rumex acetosa* | Polygonaceae | *Rumex* | Herbaceous | No |
| *Trisetum spicatum* | Poaceae | *Trisetum* | Herbaceous | No |
| *Cerastium tianschanicum* | Caryophyllaceae | *Cerastium* | Herbaceous | No |
| *Ranunculus popovii* | Ranunculaceae | *Ranunculus* | Herbaceous | Yes |
| *Seriphidium nitrosum* | Asteraceae | *Seriphidium* | Woody | Yes |
| *Arctopoa tibetica* | Poaceae | *Arctopoa* | Herbaceous | Yes |
| *Lappula semiglabra* | Boraginaceae | *Lappula* | Herbaceous | Yes |
| *Polygala hybrida* | Polygalaceae | *Polygala* | Herbaceous | No |
| *Aster lingii* | Asteraceae | *Aster* | Herbaceous | No |
| *Lactuca serriola* | Asteraceae | *Lactuca* | Herbaceous | No |
| *Artemisia austriaca* | Asteraceae | *Artemisia* | Herbaceous | Yes |
| *Salix argyracea* | Salicaceae | *Salix* | Woody | No |
| *Calligonum yengisaricum* | Polygonaceae | *Calligonum* | Woody | Yes |
| *Juncus leucomelas* | Juncaceae | *Juncus* | Herbaceous | No |
| *Typha domingensis* | Typhaceae | *Typha* | Herbaceous | Yes |
| *Stenosolenium saxatile* | Boraginaceae | *Stenosolenium* | Herbaceous | No |
| *Veronica anagallis-aquatica* | Plantaginaceae | *Veronica* | Herbaceous | No |
| *Corispermum dutreuilii* | Amaranthaceae | *Corispermum* | Herbaceous | No |
| *Euphorbia humifusa* | Euphorbiaceae | *Euphorbia* | Herbaceous | Yes |
| *Festuca brachyphylla* | Poaceae | *Festuca* | Herbaceous | No |
| *Taraxacum compactum* | Asteraceae | *Taraxacum* | Herbaceous | Yes |
| *Taraxacum dissectum* | Asteraceae | *Taraxacum* | Herbaceous | Yes |
| *Lappula ferganensis* | Boraginaceae | *Lappula* | Herbaceous | No |
| *Caragana pruinosa* | Fabaceae | *Caragana* | Woody | No |
| *Poa albertii subsp. kunlunensis* | Poaceae | *Poa* | Herbaceous | Yes |
| *Bassia hyssopifolia* | Amaranthaceae | *Bassia* | Herbaceous | Yes |
| *Persicaria orientalis* | Polygonaceae | *Persicaria* | Herbaceous | Yes |
| *Corydalis mira* | Papaveraceae | *Corydalis* | Woody | No |
| *Stipa roborowskyi* | Poaceae | *Stipa* | Herbaceous | Yes |
| *Oxytropis coerulea* | Fabaceae | *Oxytropis* | Herbaceous | Yes |
| *Ranunculus pedatifidus* | Ranunculaceae | *Ranunculus* | Herbaceous | No |
| *Lappula patula* | Boraginaceae | *Lappula* | Herbaceous | No |
| *Rosa spinosissima* | Rosaceae | *Rosa* | Woody | No |
| *Atraphaxis frutescens* | Polygonaceae | *Atraphaxis* | Woody | No |
| *Lepidium ferganense* | Brassicaceae | *Lepidium* | Herbaceous | Yes |
| *Eutrema fontanum* | Brassicaceae | *Eutrema* | Herbaceous | No |
| *Chondrilla ambigua* | Asteraceae | *Chondrilla* | Herbaceous | Yes |
| *Goldbachia laevigata* | Brassicaceae | *Goldbachia* | Herbaceous | No |
| *Rheum wittrockii* | Polygonaceae | *Rheum* | Herbaceous | No |
| *Diarthron tianschanicum* | Thymelaeaceae | *Diarthron* | Herbaceous | No |
| *Potentilla nervosa* | Rosaceae | *Potentilla* | Herbaceous | No |
| *Vicia costata* | Fabaceae | *Vicia* | Herbaceous | No |
| *Taraxacum officinale* | Asteraceae | *Taraxacum* | Herbaceous | Yes |
| *Poa annua* | Poaceae | *Poa* | Herbaceous | Yes |
| *Halerpestes ruthenica* | Ranunculaceae | *Halerpestes* | Herbaceous | No |
| *Gentiana kaufmanniana* | Gentianaceae | *Gentiana* | Herbaceous | No |
| *Potentilla saundersiana* | Rosaceae | *Potentilla* | Herbaceous | No |
| *Eritrichium pseudolatifolium* | Boraginaceae | *Eritrichium* | Herbaceous | No |
| *Oxytropis myriophylla* | Fabaceae | *Oxytropis* | Herbaceous | No |
| *Taraxacum dealbatum* | Asteraceae | *Taraxacum* | Herbaceous | Yes |
| *Puccinellia hackeliana* | Poaceae | *Puccinellia* | Herbaceous | Yes |
| *Stephanachne pappophorea* | Poaceae | *Stephanachne* | Herbaceous | Yes |
| *Askellia pygmaea* | Asteraceae | *Askellia* | Herbaceous | No |
| *Trigonella cachemiriana* | Fabaceae | *Trigonella* | Herbaceous | No |
| *Senecio nemorensis* | Asteraceae | *Senecio* | Herbaceous | No |
| *Saxifraga cernua* | Saxifragaceae | *Saxifraga* | Herbaceous | No |
| *Elymus tangutorum* | Poaceae | *Elymus* | Herbaceous | Yes |
| *Festuca rubra subsp. arctica* | Poaceae | *Festuca* | Herbaceous | No |
| *Tanacetum pulchrum* | Asteraceae | *Tanacetum* | Herbaceous | No |
| *Spergularia marina* | Caryophyllaceae | *Spergularia* | Herbaceous | No |
| *Cyperus serotinus* | Cyperaceae | *Cyperus* | Herbaceous | No |
| *Alopecurus arundinaceus* | Poaceae | *Alopecurus* | Herbaceous | No |
| *Stipa regeliana* | Poaceae | *Stipa* | Herbaceous | No |
| *Astragalus laxmannii* | Fabaceae | *Astragalus* | Herbaceous | Yes |
| *Oxytropis chionobia* | Fabaceae | *Oxytropis* | Herbaceous | Yes |
| *Chenopodium strictum* | Amaranthaceae | *Chenopodium* | Herbaceous | Yes |
| *Polygonum patulum* | Polygonaceae | *Polygonum* | Herbaceous | No |
| *Digitaria ischaemum* | Poaceae | *Digitaria* | Herbaceous | Yes |
| *Chondrilla maracandica* | Asteraceae | *Chondrilla* | Herbaceous | No |
| *Vicatia atrosanguinea* | Apiaceae | *Vicatia* | Herbaceous | No |
| *Saussurea leucophylla* | Asteraceae | *Saussurea* | Herbaceous | No |
| *Taraxacum pseudominutilobum* | Asteraceae | *Taraxacum* | Herbaceous | Yes |
| *Anabasis brevifolia* | Amaranthaceae | *Anabasis* | Woody | No |
| *Astragalus obtusifoliolus* | Fabaceae | *Astragalus* | Herbaceous | No |
| *Lomelosia alpestris* | Caprifoliaceae | *Lomelosia* | Herbaceous | No |
| *Aster hispidus* | Asteraceae | *Aster* | Herbaceous | Yes |
| *Linum amurense* | Linaceae | *Linum* | Herbaceous | No |
| *Elymus atratus* | Poaceae | *Elymus* | Herbaceous | Yes |
| *Clematis intricata* | Ranunculaceae | *Clematis* | Herbaceous | Yes |
| *Tripolium pannonicum* | Asteraceae | *Tripolium* | Herbaceous | No |
| *Suaeda corniculata* | Amaranthaceae | *Suaeda* | Herbaceous | Yes |
| *Pedicularis violascens* | Orobanchaceae | *Pedicularis* | Herbaceous | Yes |
| *Sporobolus schoenoides* | Poaceae | *Sporobolus* | Herbaceous | Yes |
| *Galium verum var. tomentosum* | Rubiaceae | *Galium* | Herbaceous | No |
| *Lythrum salicaria* | Lythraceae | *Lythrum* | Herbaceous | Yes |
| *Gentiana macrophylla* | Gentianaceae | *Gentiana* | Herbaceous | No |
| *Tamarix taklamakanensis* | Tamaricaceae | *Tamarix* | Woody | No |
| *Myriophyllum spicatum* | Haloragaceae | *Myriophyllum* | Herbaceous | No |
| *Astragalus petraeus* | Fabaceae | *Astragalus* | Woody | No |
| *Lotus frondosus* | Fabaceae | *Lotus* | Herbaceous | Yes |
| *Schoenoplectus litoralis* | Cyperaceae | *Schoenoplectus* | Herbaceous | No |
| *Anabasis truncata* | Amaranthaceae | *Anabasis* | Woody | No |
| *Trifolium fragiferum* | Fabaceae | *Trifolium* | Herbaceous | No |
| *Anabasis pelliotii* | Amaranthaceae | *Anabasis* | Woody | No |
| *Typha davidiana* | Typhaceae | *Typha* | Herbaceous | Yes |
| *Carex nudicarpa* | Cyperaceae | *Carex* | Herbaceous | No |
| *Eleocharis uniglumis* | Cyperaceae | *Eleocharis* | Herbaceous | No |
| *Astragalus borodinii* | Fabaceae | *Astragalus* | Herbaceous | No |
| *Deschampsia cespitosa* | Poaceae | *Deschampsia* | Herbaceous | No |
| *Onosma setosa* | Boraginaceae | *Onosma* | Herbaceous | No |
| *Eragrostis pilosa* | Poaceae | *Eragrostis* | Herbaceous | Yes |
| *Mentha asiatica* | Lamiaceae | *Mentha* | Herbaceous | No |
| *Erodium oxyrhinchum* | Geraniaceae | *Erodium* | Herbaceous | No |
| *Ceratocephala testiculata* | Ranunculaceae | *Ceratocephala* | Herbaceous | No |
| *Enneapogon desvauxii* | Poaceae | *Enneapogon* | Herbaceous | No |
| *Zygophyllum kaschgaricum* | Zygophyllaceae | *Zygophyllum* | Woody | No |
| *Lappula consanguinea* | Boraginaceae | *Lappula* | Herbaceous | No |
| *Equisetum scirpoides* | Equisetaceae | *Equisetum* | Herbaceous | No |
| *Gentiana straminea* | Gentianaceae | *Gentiana* | Herbaceous | No |
| *Asterothamnus poliifolius* | Asteraceae | *Asterothamnus* | Woody | Yes |
| *Orobanche amoena* | Orobanchaceae | *Orobanche* | Herbaceous | No |
| *Juncus heptopotamicus* | Juncaceae | *Juncus* | Herbaceous | No |
| *Sisymbriopsis mollipila* | Brassicaceae | *Sisymbriopsis* | Herbaceous | No |
| *Angelica ternata* | Apiaceae | *Angelica* | Herbaceous | No |
| *Cotoneaster oliganthus* | Rosaceae | *Cotoneaster* | Woody | No |
| *Cynomorium songaricum* | Cynomoriaceae | *Cynomorium* | Herbaceous | Yes |
| *Deyeuxia tianschanica* | Poaceae | *Deyeuxia* | Herbaceous | No |
| *Dracocephalum moldavica* | Lamiaceae | *Dracocephalum* | Herbaceous | Yes |
| *Dasiphora parvifolia* | Rosaceae | *Dasiphora* | Woody | No |
| *Salicornia europaea* | Amaranthaceae | *Salicornia* | Herbaceous | Yes |
| *Cistanche salsa* | Orobanchaceae | *Cistanche* | Herbaceous | No |
| *Leymus chinensis* | Poaceae | *Leymus* | Herbaceous | Yes |
| *Veronica ciliata* | Plantaginaceae | *Veronica* | Herbaceous | No |
| *Plantago lanceolata* | Plantaginaceae | *Plantago* | Herbaceous | No |
| *Atraphaxis virgata* | Polygonaceae | *Atraphaxis* | Woody | No |
| *Rorippa palustris* | Brassicaceae | *Rorippa* | Herbaceous | Yes |
| *Dianthus acicularis* | Caryophyllaceae | *Dianthus* | Herbaceous | No |
| *Rumex popovii* | Polygonaceae | *Rumex* | Herbaceous | No |
| *Chorispora songarica* | Brassicaceae | *Chorispora* | Herbaceous | No |
| *Dianthus soongoricus* | Caryophyllaceae | *Dianthus* | Herbaceous | No |
| *Eremurus altaicus* | Asphodelaceae | *Eremurus* | Herbaceous | No |
| *Schulzia albiflora* | Apiaceae | *Schulzia* | Herbaceous | No |
| *Astragalus beketowii* | Fabaceae | *Astragalus* | Herbaceous | No |
| *Saussurea pulviniformis* | Asteraceae | *Saussurea* | Herbaceous | No |
| *Stellaria brachypetala* | Caryophyllaceae | *Stellaria* | Herbaceous | No |
| *Orthilia obtusata* | Ericaceae | *Orthilia* | Woody | No |
| *Stipa tianschanica var. gobica* | Poaceae | *Stipa* | Herbaceous | No |
| *Erigeron schmalhausenii* | Asteraceae | *Erigeron* | Herbaceous | No |
| *Crepis rigescens* | Asteraceae | *Crepis* | Herbaceous | Yes |
| *Aphragmus oxycarpus* | Brassicaceae | *Aphragmus* | Herbaceous | No |
| *Cotoneaster melanocarpus* | Rosaceae | *Cotoneaster* | Woody | No |
| *Eremogone festucoides* | Caryophyllaceae | *Eremogone* | Herbaceous | No |
| *Eritrichium canum* | Boraginaceae | *Eritrichium* | Herbaceous | Yes |
| *Dysphania schraderiana* | Amaranthaceae | *Dysphania* | Herbaceous | No |
| *Anchusa ovata* | Boraginaceae | *Anchusa* | Herbaceous | Yes |
| *Cerastium falcatum* | Caryophyllaceae | *Cerastium* | Herbaceous | No |
| *Geum aleppicum* | Rosaceae | *Geum* | Herbaceous | No |
| *Urtica cannabina* | Urticaceae | *Urtica* | Herbaceous | No |
| *Koeleria litvinowii* | Poaceae | *Koeleria* | Herbaceous | Yes |
| *Erysimum flavum* | Brassicaceae | *Erysimum* | Herbaceous | No |
| *Eutrema heterophyllum* | Brassicaceae | *Eutrema* | Herbaceous | No |
| *Thymelaea passerina* | Thymelaeaceae | *Thymelaea* | Herbaceous | No |
| *Paraquilegia anemonoides* | Ranunculaceae | *Paraquilegia* | Herbaceous | No |
| *Ammopiptanthus mongolicus* | Fabaceae | *Ammopiptanthus* | Woody | No |
| *Veronica undulata* | Plantaginaceae | *Veronica* | Herbaceous | No |
| *Oreosalsola laricifolia* | Amaranthaceae | *Oreosalsola* | Woody | Yes |
| *Olimarabidopsis pumila* | Brassicaceae | *Olimarabidopsis* | Herbaceous | No |
| *Suaeda corniculata var. olufsenii* | Amaranthaceae | *Suaeda* | Herbaceous | Yes |
| *Silene himalayensis* | Caryophyllaceae | *Silene* | Herbaceous | No |
| *Elsholtzia ciliata* | Lamiaceae | *Elsholtzia* | Herbaceous | No |
| *Potamogeton pusillus* | Potamogetonaceae | *Potamogeton* | Herbaceous | Yes |
| *Taraxacum goloskokovii* | Asteraceae | *Taraxacum* | Herbaceous | No |
| *Gentiana decumbens* | Gentianaceae | *Gentiana* | Herbaceous | No |
| *Adenophora liliifolia* | Campanulaceae | *Adenophora* | Herbaceous | No |
| *Catabrosa aquatica* | Poaceae | *Catabrosa* | Herbaceous | No |
| *Rosa multiflora* | Rosaceae | *Rosa* | Woody | No |
| *Berberis heteropoda* | Berberidaceae | *Berberis* | Woody | Yes |
| *Youngia heterophylla* | Asteraceae | *Youngia* | Herbaceous | Yes |
| *Veronica beccabunga subsp. muscosa* | Plantaginaceae | *Veronica* | Herbaceous | No |
| *Ulmus pumila* | Ulmaceae | *Ulmus* | Woody | Yes |
| *Chenopodiastrum hybridum* | Amaranthaceae | *Chenopodiastrum* | Herbaceous | Yes |
| *Corispermum heptapotamicum* | Amaranthaceae | *Corispermum* | Herbaceous | Yes |
| *Artemisia songarica* | Asteraceae | *Artemisia* | Woody | Yes |
| *Silene vulgaris* | Caryophyllaceae | *Silene* | Herbaceous | No |
| *Alfredia acantholepis* | Asteraceae | *Alfredia* | Herbaceous | No |
| *Ephedra fedtschenkoae* | Ephedraceae | *Ephedra* | Woody | Yes |
| *Semenovia transiliensis* | Apiaceae | *Semenovia* | Herbaceous | No |
| *Atriplex centralasiatica var. megalotheca* | Amaranthaceae | *Atriplex* | Herbaceous | No |
| *Astragalus bhotanensis* | Fabaceae | *Astragalus* | Herbaceous | No |
| *Androsace tapete* | Primulaceae | *Androsace* | Herbaceous | No |
| *Archangelica brevicaulis* | Apiaceae | *Archangelica* | Herbaceous | No |
| *Saussurea pulvinata* | Asteraceae | *Saussurea* | Herbaceous | No |
| *Phleum alpinum* | Poaceae | *Phleum* | Herbaceous | No |
| *Oxytropis immersa* | Fabaceae | *Oxytropis* | Herbaceous | No |
| *Saussurea inversa* | Asteraceae | *Saussurea* | Herbaceous | No |
| *Thymus proximus* | Lamiaceae | *Thymus* | Woody | No |
| *Rumex pseudonatronatus* | Polygonaceae | *Rumex* | Herbaceous | No |
| *Scutellaria przewalskii* | Lamiaceae | *Scutellaria* | Woody | No |
| *Rhodiola quadrifida* | Crassulaceae | *Rhodiola* | Herbaceous | No |
| *Kengyilia thoroldiana* | Poaceae | *Kengyilia* | Herbaceous | No |
| *Allium glomeratum* | Amaryllidaceae | *Allium* | Herbaceous | No |
| *Calamagrostis turkestanica* | Poaceae | *Calamagrostis* | Herbaceous | No |
| *Gentiana clarkei* | Gentianaceae | *Gentiana* | Herbaceous | No |
| *Iljinskaea planisiliqua* | Brassicaceae | *Iljinskaea* | Herbaceous | No |
| *Convolvulus gortschakovii* | Convolvulaceae | *Convolvulus* | Woody | No |
| *Alisma plantago-aquatica* | Alismataceae | *Alisma* | Herbaceous | No |
| *Potentilla angustiloba* | Rosaceae | *Potentilla* | Herbaceous | No |
| *Potamogeton crispus* | Potamogetonaceae | *Potamogeton* | Herbaceous | Yes |
| *Jurinea algida* | Asteraceae | *Jurinea* | Herbaceous | No |
| *Dactylorhiza viridis* | Orchidaceae | *Dactylorhiza* | Herbaceous | No |
| *Astragalus variabilis* | Fabaceae | *Astragalus* | Herbaceous | No |
| *Juncus ranarius* | Juncaceae | *Juncus* | Herbaceous | No |
| *Achnatherum saposhnikovii* | Poaceae | *Achnatherum* | Herbaceous | No |
| *Elytrigia repens subsp. elongatiformis* | Poaceae | *Elytrigia* | Herbaceous | No |
| *Carduus nutans* | Asteraceae | *Carduus* | Herbaceous | Yes |
| *Poa pratensis subsp. pruinosa* | Poaceae | *Poa* | Herbaceous | No |
| *Oxytropis kansuensis* | Fabaceae | *Oxytropis* | Herbaceous | No |
| *Urtica hyperborea* | Urticaceae | *Urtica* | Herbaceous | No |
| *Lipschitzia divaricata* | Asteraceae | *Lipschitzia* | Herbaceous | No |
| *Epipactis helleborine* | Orchidaceae | *Epipactis* | Herbaceous | Yes |
| *Fallopia convolvulus* | Polygonaceae | *Fallopia* | Herbaceous | No |
| *Anemone gortschakowii* | Ranunculaceae | *Anemone* | Herbaceous | No |
| *Saussurea cinerea* | Asteraceae | *Saussurea* | Herbaceous | Yes |
| *Lagochilus platyacanthus* | Lamiaceae | *Lagochilus* | Herbaceous | No |
| *Chamerion angustifolium* | Onagraceae | *Chamerion* | Herbaceous | No |
| *Digitaria sanguinalis* | Poaceae | *Digitaria* | Herbaceous | Yes |
| *Erigeron lachnocephalus* | Asteraceae | *Erigeron* | Herbaceous | No |
| *Lagochilus lanatonodus* | Lamiaceae | *Lagochilus* | Herbaceous | No |
| *Xylosalsola arbuscula* | Amaranthaceae | *Xylosalsola* | Woody | No |
| *Corydalis hendersonii* | Papaveraceae | *Corydalis* | Herbaceous | No |
| *Puccinellia pamirica* | Poaceae | *Puccinellia* | Herbaceous | No |
| *Axyris prostrata* | Amaranthaceae | *Axyris* | Herbaceous | No |
| *Sedobassia sedoides* | Amaranthaceae | *Sedobassia* | Herbaceous | No |
| *Artemisia desertorum* | Asteraceae | *Artemisia* | Herbaceous | Yes |
| *Linum perenne* | Linaceae | *Linum* | Herbaceous | No |
| *Deschampsia koelerioides* | Poaceae | *Deschampsia* | Herbaceous | Yes |
| *Alchemilla tianschanica* | Rosaceae | *Alchemilla* | Herbaceous | No |
| *Parnassia oreophila* | Celastraceae | *Parnassia* | Herbaceous | Yes |
| *Gagea tenera* | Liliaceae | *Gagea* | Herbaceous | No |
| *Rhodiola kirilowii* | Crassulaceae | *Rhodiola* | Herbaceous | No |
| *Saussurea schlagintweitii* | Asteraceae | *Saussurea* | Herbaceous | No |
| *Ligularia heterophylla* | Asteraceae | *Ligularia* | Herbaceous | No |
| *Pyrola rotundifolia* | Ericaceae | *Pyrola* | Woody | No |
| *Taraxacum bessarabicum* | Asteraceae | *Taraxacum* | Herbaceous | No |
| *Comastoma pedunculatum* | Gentianaceae | *Comastoma* | Herbaceous | Yes |
| *Suaeda pterantha* | Amaranthaceae | *Suaeda* | Herbaceous | No |
| *Pedicularis alaschanica* | Orobanchaceae | *Pedicularis* | Herbaceous | No |
| *Festuca alatavica* | Poaceae | *Festuca* | Herbaceous | No |
| *Gentianella pygmaea* | Gentianaceae | *Gentianella* | Herbaceous | No |
| *Chondrilla leiosperma* | Asteraceae | *Chondrilla* | Herbaceous | No |
| *Carex stipitiutriculata* | Cyperaceae | *Carex* | Herbaceous | No |
| *Saussurea amara* | Asteraceae | *Saussurea* | Herbaceous | No |
| *Tragopogon stepposus* | Asteraceae | *Tragopogon* | Herbaceous | No |
| *Geranium divaricatum* | Geraniaceae | *Geranium* | Herbaceous | No |
| *Lonicera simulatrix* | Caprifoliaceae | *Lonicera* | Woody | No |
| *Salsola zaidamica* | Amaranthaceae | *Salsola* | Herbaceous | No |
| *Goldbachia pendula* | Brassicaceae | *Goldbachia* | Herbaceous | No |
| *Sabulina verna* | Caryophyllaceae | *Sabulina* | Herbaceous | No |
| *Astragalus oplites* | Fabaceae | *Astragalus* | Woody | No |
| *Carex atrata subsp. aterrima* | Cyperaceae | *Carex* | Herbaceous | No |
| *Tetrataenium olgae* | Apiaceae | *Tetrataenium* | Herbaceous | No |
| *Androsace umbellata* | Primulaceae | *Androsace* | Herbaceous | No |
| *Linum pallescens* | Linaceae | *Linum* | Herbaceous | No |
| *Oxytropis biloba* | Fabaceae | *Oxytropis* | Herbaceous | No |
| *Spergularia diandra* | Caryophyllaceae | *Spergularia* | Herbaceous | No |
| *Stellaria media* | Caryophyllaceae | *Stellaria* | Herbaceous | No |
| *Anemone narcissiflora subsp. protracta* | Ranunculaceae | *Anemone* | Herbaceous | No |
| *Ranunculus natans* | Ranunculaceae | *Ranunculus* | Herbaceous | No |
| *Gentiana algida* | Gentianaceae | *Gentiana* | Herbaceous | No |
| *Poa pratensis subsp. alpigena* | Poaceae | *Poa* | Herbaceous | No |
| *Allium semenovii* | Amaryllidaceae | *Allium* | Herbaceous | No |
| *Echinochloa colona* | Poaceae | *Echinochloa* | Herbaceous | Yes |
| *Juncus atratus* | Juncaceae | *Juncus* | Herbaceous | No |
| *Berberis amurensis* | Berberidaceae | *Berberis* | Woody | Yes |
| *Astragalus membranaceus* | Fabaceae | *Astragalus* | Herbaceous | Yes |
| *Bromus oxyodon* | Poaceae | *Bromus* | Herbaceous | No |
| *Thalictrum simplex* | Ranunculaceae | *Thalictrum* | Herbaceous | No |
| *Saussurea depsangensis* | Asteraceae | *Saussurea* | Herbaceous | No |
| *Gentiana scabra* | Gentianaceae | *Gentiana* | Herbaceous | No |
| *Pedicularis verticillata* | Orobanchaceae | *Pedicularis* | Herbaceous | No |
| *Silene repens* | Caryophyllaceae | *Silene* | Herbaceous | No |
| *Erodium stephanianum* | Geraniaceae | *Erodium* | Herbaceous | No |
| *Lagopsis eriostachys* | Lamiaceae | *Lagopsis* | Herbaceous | No |
| *Ranunculus trautvetterianus* | Ranunculaceae | *Ranunculus* | Herbaceous | No |
| *Elymus durus* | Poaceae | *Elymus* | Herbaceous | No |
| *Ranunculus brotherusii* | Ranunculaceae | *Ranunculus* | Herbaceous | No |
| *Lycopus europaeus* | Lamiaceae | *Lycopus* | Herbaceous | No |
| *Oxytropis qitaiensis* | Fabaceae | *Oxytropis* | Herbaceous | No |
| *Equisetum palustre* | Equisetaceae | *Equisetum* | Herbaceous | No |
| *Cistanche deserticola* | Orobanchaceae | *Cistanche* | Herbaceous | Yes |
| *Achillea millefolium* | Asteraceae | *Achillea* | Herbaceous | No |
| *Pseudolysimachion spicatum* | Plantaginaceae | *Pseudolysimachion* | Herbaceous | No |
| *Bupleurum thianschanicum* | Apiaceae | *Bupleurum* | Herbaceous | No |
| *Vickifunkia tianschanica* | Asteraceae | *Vickifunkia* | Herbaceous | No |
| *Silene tianschanica* | Caryophyllaceae | *Silene* | Woody | No |
| *Goniolimon speciosum* | Plumbaginaceae | *Goniolimon* | Herbaceous | No |
| *Poa sibirica* | Poaceae | *Poa* | Herbaceous | No |
| *Rubia tibetica* | Rubiaceae | *Rubia* | Herbaceous | No |
| *Swertia graciliflora* | Gentianaceae | *Swertia* | Herbaceous | No |
| *Kalidium gracile* | Amaranthaceae | *Kalidium* | Woody | No |
| *Rumex stenophyllus* | Polygonaceae | *Rumex* | Herbaceous | No |
| *Eragrostis suaveolens* | Poaceae | *Eragrostis* | Herbaceous | No |
| *Taraxacum koksaghyz* | Asteraceae | *Taraxacum* | Herbaceous | No |
| *Potamogeton nodosus* | Potamogetonaceae | *Potamogeton* | Herbaceous | No |
| *Polygonum schischkinii* | Polygonaceae | *Polygonum* | Woody | Yes |
| *Alajja anomala* | Lamiaceae | *Alajja* | Herbaceous | No |
| *Cuscuta campestris* | Convolvulaceae | *Cuscuta* | Herbaceous | No |
| *Astragalus monophyllus* | Fabaceae | *Astragalus* | Herbaceous | No |
| *Silene pseudotenuis* | Caryophyllaceae | *Silene* | Herbaceous | No |
| *Pyankovia roborowskii* | Amaranthaceae | *Pyankovia* | Herbaceous | No |
| *Oxytropis proboscidea* | Fabaceae | *Oxytropis* | Herbaceous | No |
| *Erigeron petiolaris* | Asteraceae | *Erigeron* | Herbaceous | No |
| *Seriphidium schrenkianum* | Asteraceae | *Seriphidium* | Herbaceous | No |
| *Mesostemma dichotomum* | Caryophyllaceae | *Mesostemma* | Herbaceous | No |
| *Dracocephalum grandiflorum* | Lamiaceae | *Dracocephalum* | Herbaceous | No |
| *Orthilia secunda* | Ericaceae | *Orthilia* | Herbaceous | No |
| *Cuscuta monogyna* | Convolvulaceae | *Cuscuta* | Herbaceous | No |
| *Polygonum junceum* | Polygonaceae | *Polygonum* | Herbaceous | No |
| *Aegopodium alpestre* | Apiaceae | *Aegopodium* | Herbaceous | No |
| *Elymus confusus var. breviaristatus* | Poaceae | *Stipagrostis* | Herbaceous | No |
| *Cherleria biflora* | Caryophyllaceae | *Cherleria* | Herbaceous | No |
| *Sanguisorba alpina* | Rosaceae | *Sanguisorba* | Herbaceous | No |
| *Salix matsudana* | Salicaceae | *Salix* | Woody | No |
| *Trigonella foenum-graecum* | Fabaceae | *Trigonella* | Herbaceous | No |
| *Cyperus pannonicus* | Cyperaceae | *Cyperus* | Herbaceous | No |
| *Eleocharis valleculosa var. setosa* | Cyperaceae | *Eleocharis* | Herbaceous | No |
| *Astragalus platyphyllus* | Fabaceae | *Astragalus* | Herbaceous | No |
| *Lappula redowskii* | Boraginaceae | *Lappula* | Herbaceous | No |
| *Crepis lignea* | Asteraceae | *Crepis* | Herbaceous | No |
| *Artemisia vestita* | Asteraceae | *Artemisia* | Woody | No |
| *Arctium tomentosum* | Asteraceae | *Arctium* | Herbaceous | No |
| *Eleusine indica* | Poaceae | *Eleusine* | Herbaceous | No |
| *Saxifraga oppositifolia* | Saxifragaceae | *Saxifraga* | Herbaceous | No |
| *Sium latifolium* | Apiaceae | *Sium* | Herbaceous | No |
| *Myricaria prostrata* | Tamaricaceae | *Myricaria* | Woody | No |
| *Aster boweri* | Asteraceae | *Aster* | Herbaceous | No |
| *Elymus abolinii var. divaricans* | Poaceae | *Elymus* | Herbaceous | No |
| *Bistorta officinalis* | Polygonaceae | *Bistorta* | Herbaceous | No |
| *Atraphaxis compacta* | Polygonaceae | *Atraphaxis* | Woody | No |
| *Atraphaxis pungens* | Polygonaceae | *Atraphaxis* | Woody | No |
| *Oxytropis racemosa* | Fabaceae | *Oxytropis* | Herbaceous | No |
| *Agrostis capillaris* | Poaceae | *Agrostis* | Herbaceous | No |
| *Gentiana tianschanica* | Gentianaceae | *Gentiana* | Herbaceous | No |
| *Gypsophila capituliflora* | Caryophyllaceae | *Gypsophila* | Herbaceous | No |
| *Astragalus arnoldii* | Fabaceae | *Astragalus* | Herbaceous | No |
| *Oxytropis puberula* | Fabaceae | *Oxytropis* | Herbaceous | No |
| *Klasea lyratifolia* | Asteraceae | *Klasea* | Herbaceous | No |
| *Glycyrrhiza eglandulosa* | Fabaceae | *Glycyrrhiza* | Herbaceous | Yes |
| *Carex capillaris* | Cyperaceae | *Carex* | Herbaceous | No |
| *Crepidiastrum tenuifolium* | Asteraceae | *Crepidiastrum* | Herbaceous | Yes |
| *Solms-laubachia linearis* | Brassicaceae | *Solms-laubachia* | Herbaceous | No |
| *Dontostemon glandulosus* | Brassicaceae | *Dontostemon* | Herbaceous | No |
| *Oxytropis microphylla* | Fabaceae | *Oxytropis* | Herbaceous | No |
| *Ligustrum quihoui* | Oleaceae | *Ligustrum* | Woody | Yes |
| *Puccinellia tenuiflora* | Poaceae | *Puccinellia* | Herbaceous | No |
| *Poa supina* | Poaceae | *Poa* | Herbaceous | No |
| *Salix iliensis* | Salicaceae | *Salix* | Woody | No |
| *Helictochloa hookeri* | Poaceae | *Helictochloa* | Herbaceous | No |
| *Dactylorhiza umbrosa* | Orchidaceae | *Dactylorhiza* | Herbaceous | No |
| *Eleocharis argyrolepis* | Cyperaceae | *Eleocharis* | Herbaceous | No |
| *Cryptospora falcata* | Brassicaceae | *Cryptospora* | Herbaceous | No |
| *Cousinia sclerolepis* | Asteraceae | *Cousinia* | Herbaceous | No |
| *Juncus thomsonii* | Juncaceae | *Juncus* | Herbaceous | No |
| *Stipa capillata* | Poaceae | *Stipa* | Herbaceous | No |
| *Anzhengxia yechengnica* | Brassicaceae | *Anzhengxia* | Herbaceous | No |
| *Galatella chromopappa* | Asteraceae | *Galatella* | Herbaceous | No |
| *Draba involucrata* | Brassicaceae | *Draba* | Herbaceous | No |
| *Stipa subsessiliflora* | Poaceae | *Stipa* | Herbaceous | Yes |
| *Aquilegia atrovinosa* | Ranunculaceae | *Aquilegia* | Herbaceous | No |
| *Anabasis cretacea* | Amaranthaceae | *Anabasis* | Herbaceous | No |
| *Lepidium virginicum* | Brassicaceae | *Lepidium* | Herbaceous | No |
| *Rhinanthus glaber* | Orobanchaceae | *Rhinanthus* | Herbaceous | No |
| *Thalictrum foetidum var. glabrescens* | Ranunculaceae | *Thalictrum* | Herbaceous | No |
| *Poa albertii subsp. poophagorum* | Poaceae | *Poa* | Herbaceous | No |
| *Calligonum squarrosum* | Polygonaceae | *Calligonum* | Woody | No |
| *Cerastium fontanum subsp. vulgare* | Caryophyllaceae | *Cerastium* | Herbaceous | No |
| *Gentiana subuniflora* | Gentianaceae | *Gentiana* | Herbaceous | No |
| *Tanacetum richterioides* | Asteraceae | *Tanacetum* | Herbaceous | No |
| *Lamium album* | Lamiaceae | *Lamium* | Herbaceous | No |
| *Oxytropis hirsutiuscula* | Fabaceae | *Oxytropis* | Herbaceous | No |
| *Suaeda altissima* | Amaranthaceae | *Suaeda* | Herbaceous | No |
| *Spiraea alpina* | Rosaceae | *Spiraea* | Woody | No |
| *Artemisia phaeolepis* | Asteraceae | *Artemisia* | Herbaceous | No |
| *Astragalus melanostachys* | Fabaceae | *Astragalus* | Herbaceous | No |
| *Caragana rosea* | Fabaceae | *Caragana* | Woody | No |
| *Polemonium caeruleum* | Polemoniaceae | *Polemonium* | Herbaceous | No |
| *Smelowskia alba* | Brassicaceae | *Smelowskia* | Herbaceous | No |
| *Saussurea pseudosalsa* | Asteraceae | *Saussurea* | Herbaceous | No |
| *Lotus angustissimus* | Fabaceae | *Lotus* | Herbaceous | No |
| *Lepidium cartilagineum* | Brassicaceae | *Lepidium* | Herbaceous | No |
| *Allium polyrhizum* | Amaryllidaceae | *Allium* | Herbaceous | No |
| *Soda inermis* | Amaranthaceae | *Soda* | Herbaceous | No |
| *Silene karaczukuri* | Caryophyllaceae | *Silene* | Herbaceous | No |
| *Artemisia dracunculus var. turkestanica* | Asteraceae | *Artemisia* | Herbaceous | No |
| *Suaeda crassifolia* | Amaranthaceae | *Suaeda* | Herbaceous | No |
| *Puccinellia nudiflora* | Poaceae | *Puccinellia* | Herbaceous | No |
| *Ranunculus japonicus* | Ranunculaceae | *Ranunculus* | Herbaceous | No |
| *Astragalus lasiosemius* | Fabaceae | *Astragalus* | Woody | No |
| *Barbarea vulgaris* | Brassicaceae | *Barbarea* | Herbaceous | No |
| *Cnidium monnieri* | Apiaceae | *Cnidium* | Herbaceous | No |
| *Carex myosuroides* | Cyperaceae | *Carex* | Herbaceous | No |
| *Astragalus sogotensis* | Fabaceae | *Astragalus* | Herbaceous | No |
| *Pedicularis myriophylla* | Orobanchaceae | *Pedicularis* | Herbaceous | No |
| *Astragalus masanderanus* | Fabaceae | *Astragalus* | Herbaceous | No |
| *Carex tibetikobresia* | Cyperaceae | *Carex* | Herbaceous | No |
| *Draba tibetica* | Brassicaceae | *Draba* | Herbaceous | No |
| *Alopecurus himalaicus* | Poaceae | *Alopecurus* | Herbaceous | No |
| *Ptilagrostis mongholica* | Poaceae | *Ptilagrostis* | Herbaceous | No |
| *Hypecoum leptocarpum* | Papaveraceae | *Hypecoum* | Herbaceous | No |
| *Pedicularis ludwigii* | Orobanchaceae | *Pedicularis* | Herbaceous | No |
| *Taraxacum lipskyi* | Asteraceae | *Taraxacum* | Herbaceous | No |
| *Eritrichium subjacquemontii* | Boraginaceae | *Eritrichium* | Herbaceous | No |
| *Corydalis gortschakovii* | Papaveraceae | *Corydalis* | Herbaceous | No |
| *Seriphidium kaschgaricum* | Asteraceae | *Seriphidium* | Woody | No |
| *Aconitum sinchiangense* | Ranunculaceae | *Aconitum* | Herbaceous | No |
| *Oxytropis rupifraga* | Fabaceae | *Oxytropis* | Herbaceous | No |
| *Parrya pinnatifida* | Brassicaceae | *Parrya* | Herbaceous | No |
| *Geranium rotundifolium* | Geraniaceae | *Geranium* | Herbaceous | No |
| *Silene alexandrae* | Caryophyllaceae | *Silene* | Herbaceous | No |
| *Erigeron acris subsp. politus* | Asteraceae | *Erigeron* | Herbaceous | No |
| *Carex onoei* | Cyperaceae | *Carex* | Herbaceous | No |
| *Hyoscyamus pusillus* | Solanaceae | *Hyoscyamus* | Herbaceous | No |
| *Erysimum flavum subsp. altaicum* | Brassicaceae | *Erysimum* | Herbaceous | No |
| *Echinops humilis* | Asteraceae | *Echinops* | Herbaceous | No |
| *Rhamnus minuta* | Rhamnaceae | *Rhamnus* | Woody | No |
| *Schoenoplectiella mucronata* | Cyperaceae | *Schoenoplectiella* | Herbaceous | No |
| *Carex obtusata* | Cyperaceae | *Carex* | Herbaceous | No |
| *Bromus pectinatus* | Poaceae | *Bromus* | Herbaceous | No |
| *Astragalus arkalycensis* | Fabaceae | *Astragalus* | Herbaceous | No |
| *Taraxacum qirae* | Asteraceae | *Taraxacum* | Herbaceous | No |
| *Rumex dentatus* | Polygonaceae | *Rumex* | Herbaceous | No |
| *Gentiana nutans* | Gentianaceae | *Gentiana* | Herbaceous | No |
| *Calystegia hederacea* | Convolvulaceae | *Calystegia* | Herbaceous | No |
| *Astragalus macropterus* | Fabaceae | *Astragalus* | Herbaceous | No |
| *Parthenocissus tricuspidata* | Vitaceae | *Parthenocissus* | Woody | No |
| *Artemisia minor* | Asteraceae | *Artemisia* | Herbaceous | No |
| *Saussurea japonica* | Asteraceae | *Saussurea* | Herbaceous | No |
| *Gentiana riparia* | Gentianaceae | *Gentiana* | Herbaceous | No |
| *Pycreus sanguinolentus* | Cyperaceae | *Pycreus* | Herbaceous | No |
| *Oxytropis ochrocephala* | Fabaceae | *Oxytropis* | Herbaceous | No |
| *Mentha vagans* | Lamiaceae | *Mentha* | Herbaceous | No |
| *Saussurea pseudoalpina* | Asteraceae | *Saussurea* | Herbaceous | No |
| *Pedicularis pseudocurvituba* | Orobanchaceae | *Pedicularis* | Herbaceous | No |
| *Plantago perssonii* | Plantaginaceae | *Plantago* | Herbaceous | No |
| *Nepeta taxkorganica* | Lamiaceae | *Nepeta* | Herbaceous | No |
| *Phlomoides tuberosa* | Lamiaceae | *Phlomoides* | Herbaceous | No |
| *Gagea fedtschenkoana* | Liliaceae | *Gagea* | Herbaceous | No |
| *Pedicularis sylvatica* | Orobanchaceae | *Pedicularis* | Herbaceous | No |
| *Gagea albertii* | Liliaceae | *Gagea* | Herbaceous | No |
| *Allium mongolicum* | Amaryllidaceae | *Allium* | Herbaceous | No |
| *Equisetum hyemale* | Equisetaceae | *Equisetum* | Herbaceous | No |
| *Silene aprica* | Caryophyllaceae | *Silene* | Herbaceous | No |
| *Eritrichium pamiricum* | Boraginaceae | *Eritrichium* | Herbaceous | No |
| *Saxifraga sibirica* | Saxifragaceae | *Saxifraga* | Herbaceous | No |
| *Dianthus superbus* | Caryophyllaceae | *Dianthus* | Herbaceous | No |
| *Bassia scoparia f. trichophylla* | Amaranthaceae | *Bassia* | Herbaceous | No |
| *Leymus arenarius* | Poaceae | *Leymus* | Herbaceous | No |
| *Aster eremophilus* | Asteraceae | *Aster* | Herbaceous | No |
| *Artemisia tournefortiana* | Asteraceae | *Artemisia* | Herbaceous | No |
| *Parnassia bifolia* | Celastraceae | *Parnassia* | Herbaceous | No |
| *Rumex aquaticus* | Polygonaceae | *Rumex* | Herbaceous | No |
| *Leptaleum filifolium* | Brassicaceae | *Leptaleum* | Herbaceous | No |
| *Stuckenia filiformis* | Potamogetonaceae | *Stuckenia* | Herbaceous | No |
| *Leymus tianschanicus* | Poaceae | *Leymus* | Herbaceous | No |
| *Mentha arvensis* | Lamiaceae | *Mentha* | Herbaceous | No |
| *Draba turczaninowii* | Brassicaceae | *Draba* | Herbaceous | No |
| *Poa urssulensis* | Poaceae | *Poa* | Herbaceous | No |
| *Youngia simulatrix* | Asteraceae | *Youngia* | Herbaceous | No |
| *Draba sibirica* | Brassicaceae | *Draba* | Herbaceous | No |
| *Sabulina helmii* | Caryophyllaceae | *Sabulina* | Herbaceous | No |
| *Goodyera repens* | Orchidaceae | *Goodyera* | Herbaceous | No |
| *Rosa willmottiae* | Rosaceae | *Rosa* | Woody | No |
| *Polygonatum roseum* | Asparagaceae | *Polygonatum* | Herbaceous | No |
| *Asparagus neglectus* | Asparagaceae | *Asparagus* | Herbaceous | No |
| *Sonchus asper* | Asteraceae | *Sonchus* | Herbaceous | No |
| *Vicia sepium* | Fabaceae | *Vicia* | Herbaceous | No |
| *Artemisia capillaris* | Asteraceae | *Artemisia* | Herbaceous | No |
| *Allardia tomentosa* | Asteraceae | *Allardia* | Herbaceous | No |
| *Elymus tschimganicus* | Poaceae | *Elymus* | Herbaceous | No |
| *Solenanthus circinnatus* | Boraginaceae | *Solenanthus* | Herbaceous | No |
| *Capparis himalayensis* | Capparaceae | *Capparis* | Woody | No |
| *Geranium rectum* | Geraniaceae | *Geranium* | Herbaceous | No |
| *Clematis recta* | Ranunculaceae | *Clematis* | Herbaceous | No |
| *Erigeron altaicus* | Asteraceae | *Erigeron* | Herbaceous | No |
| *Eritrichium longifolium* | Boraginaceae | *Eritrichium* | Herbaceous | No |
| *Rheum nanum* | Polygonaceae | *Rheum* | Herbaceous | No |
| *Calligonum ebinuricum* | Polygonaceae | *Calligonum* | Woody | No |
| *Thesium chinense* | Santalaceae | *Thesium* | Herbaceous | No |
| *Ranunculus banguoensis* | Ranunculaceae | *Ranunculus* | Herbaceous | No |
| *Juncus gracillimus* | Juncaceae | *Juncus* | Herbaceous | No |
| *Atriplex patens* | Amaranthaceae | *Atriplex* | Herbaceous | No |
| *Salvia yangii* | Lamiaceae | *Salvia* | Herbaceous | No |
| *Lappula pratensis* | Boraginaceae | *Lappula* | Herbaceous | No |
| *Festuca pratensis* | Poaceae | *Festuca* | Herbaceous | No |
| *Tephroseris praticola* | Asteraceae | *Tephroseris* | Herbaceous | No |
| *Seseli valentinae* | Apiaceae | *Seseli* | Herbaceous | No |
| *Artemisia hedinii* | Asteraceae | *Artemisia* | Herbaceous | No |
| *Sisymbrium heteromallum* | Brassicaceae | *Sisymbrium* | Herbaceous | No |
| *Atraphaxis spinosa* | Polygonaceae | *Atraphaxis* | Woody | No |
| *Limonium chrysocomum* | Plumbaginaceae | *Limonium* | Woody | No |
| *Saxifraga stenophylla* | Saxifragaceae | *Saxifraga* | Herbaceous | No |
| *Bupleurum aureum var. breviinvolucratum* | Apiaceae | *Bupleurum* | Herbaceous | No |
| *Erigeron multiradiatus* | Asteraceae | *Erigeron* | Herbaceous | No |
| *Hedysarum ferganense* | Fabaceae | *Hedysarum* | Herbaceous | No |
| *Trigonotis peduncularis* | Boraginaceae | *Trigonotis* | Herbaceous | No |
| *Tamarix gansuensis* | Tamaricaceae | *Tamarix* | Woody | No |
| *Thymus diminutus* | Lamiaceae | *Thymus* | Woody | No |
| *Elymus tschimganicus var. glabrispiculus* | Poaceae | *Elymus* | Herbaceous | No |
| *Bromus tectorum* | Poaceae | *Bromus* | Herbaceous | No |
| *Hippolytia kaschgarica* | Asteraceae | *Hippolytia* | Woody | No |
| *Carex melanocephala* | Cyperaceae | *Carex* | Herbaceous | No |
| *Rhodiola rosea* | Crassulaceae | *Rhodiola* | Herbaceous | No |
| *Cornus alba* | Cornaceae | *Cornus* | Woody | No |
| *Comastoma pulmonarium* | Gentianaceae | *Comastoma* | Herbaceous | No |
| *Alfredia nivea* | Asteraceae | *Alfredia* | Herbaceous | No |
| *Myriophyllum verticillatum* | Haloragaceae | *Myriophyllum* | Herbaceous | No |
| *Gentianopsis barbata var. alboflavida* | Gentianaceae | *Gentianopsis* | Herbaceous | No |
| *Chenopodium acuminatum* | Amaranthaceae | *Chenopodium* | Herbaceous | No |
| *Olgaea laniceps* | Asteraceae | *Olgaea* | Herbaceous | No |
| *Kengyilia kaschgarica* | Poaceae | *Kengyilia* | Herbaceous | No |
| *Allium platyspathum* | Amaryllidaceae | *Allium* | Herbaceous | No |
| *Zygophyllum rosowii var. latifolium* | Zygophyllaceae | *Zygophyllum* | Herbaceous | No |
| *Delphinium kunlunshanicum* | Ranunculaceae | *Delphinium* | Herbaceous | No |
| *Astragalus kunlunensis* | Fabaceae | *Astragalus* | Herbaceous | No |
| *Echinops davuricus* | Asteraceae | *Echinops* | Herbaceous | No |
| *Lonicera caerulea* | Caprifoliaceae | *Lonicera* | Woody | No |
| *Caragana aurantiaca* | Fabaceae | *Caragana* | Woody | No |
| *Aconitum nemorum* | Ranunculaceae | *Aconitum* | Herbaceous | No |
| *Gypsophila vaccaria* | Caryophyllaceae | *Gypsophila* | Herbaceous | No |
| *Verbascum thapsus* | Scrophulariaceae | *Verbascum* | Herbaceous | No |
| *Persicaria lapathifolia var. salicifolia* | Polygonaceae | *Persicaria* | Herbaceous | No |
| *Caroxylon nitrarium* | Amaranthaceae | *Caroxylon* | Herbaceous | No |
| *Caragana kirghisorum* | Fabaceae | *Caragana* | Woody | No |
| *Sisymbriopsis pamirica* | Brassicaceae | *Sisymbriopsis* | Herbaceous | No |
| *Oxytropis globiflora* | Fabaceae | *Oxytropis* | Herbaceous | No |
| *Potentilla hololeuca* | Rosaceae | *Potentilla* | Herbaceous | No |
| *Comastoma tenellum* | Gentianaceae | *Comastoma* | Herbaceous | No |
| *Cerastium pusillum* | Caryophyllaceae | *Cerastium* | Herbaceous | No |
| *Aster alpinus var. serpentimontanus* | Asteraceae | *Aster* | Herbaceous | No |
| *Myricaria germanica* | Tamaricaceae | *Myricaria* | Woody | No |
| *Gentiana aquatica* | Gentianaceae | *Gentiana* | Herbaceous | No |
| *Puccinellia schischkinii* | Poaceae | *Puccinellia* | Herbaceous | No |
| *Delphinium tianshanicum* | Ranunculaceae | *Delphinium* | Herbaceous | No |
| *Hymenidium lindleyanum* | Apiaceae | *Hymenidium* | Herbaceous | No |
| *Astragalus lepsensis* | Fabaceae | *Astragalus* | Herbaceous | No |
| *Salix turanica* | Salicaceae | *Salix* | Woody | No |
| *Orostachys fimbriata* | Crassulaceae | *Orostachys* | Herbaceous | No |
| *Thymus seravschanicus* | Lamiaceae | *Thymus* | Woody | No |
| *Scutellaria jodudiana* | Lamiaceae | *Scutellaria* | Herbaceous | No |
| *Saussurea sordida* | Asteraceae | *Saussurea* | Herbaceous | No |
| *Artemisia indica* | Asteraceae | *Artemisia* | Herbaceous | No |
| *Prunella vulgaris* | Lamiaceae | *Prunella* | Herbaceous | No |
| *Nigella glandulifera* | Ranunculaceae | *Nigella* | Herbaceous | No |
| *Potentilla longifolia* | Rosaceae | *Potentilla* | Herbaceous | No |
| *Suaeda microphylla* | Amaranthaceae | *Suaeda* | Woody | No |
| *Ziziphora bungeana* | Lamiaceae | *Ziziphora* | Woody | No |
| *Takhtajaniantha austriaca* | Asteraceae | *Takhtajaniantha* | Herbaceous | No |
| *Oxygraphis kamchatica* | Ranunculaceae | *Oxygraphis* | Herbaceous | No |
| *Plantago maritima* | Plantaginaceae | *Plantago* | Herbaceous | No |
| *Bassia stellaris* | Amaranthaceae | *Bassia* | Herbaceous | No |
| *Seriphidium transiliense* | Asteraceae | *Seriphidium* | Herbaceous | No |
| *Potentilla argentea* | Rosaceae | *Potentilla* | Herbaceous | No |
| *Galeopsis bifida* | Lamiaceae | *Galeopsis* | Herbaceous | No |
| *Alchemilla japonica* | Rosaceae | *Alchemilla* | Herbaceous | No |
| *Vicia sativa subsp. nigra* | Fabaceae | *Vicia* | Herbaceous | No |
| *Thalictrum minus var. kemense* | Ranunculaceae | *Thalictrum* | Herbaceous | No |
| *Veronica anagalloides* | Plantaginaceae | *Veronica* | Herbaceous | No |
| *Caroxylon passerinum* | Amaranthaceae | *Caroxylon* | Woody | No |
| *Galatella fastigiiformis* | Asteraceae | *Galatella* | Herbaceous | No |
| *Pedicularis songarica* | Orobanchaceae | *Pedicularis* | Herbaceous | No |
| *Tragopogon songoricus* | Asteraceae | *Tragopogon* | Herbaceous | No |
| *Dactylorhiza fuchsii* | Orchidaceae | *Dactylorhiza* | Herbaceous | No |
| *Cerastium lithospermifolium* | Caryophyllaceae | *Cerastium* | Herbaceous | No |
| *Linaria bungei* | Plantaginaceae | *Linaria* | Herbaceous | No |
| *Poa araratica* | Poaceae | *Poa* | Herbaceous | No |
| *Thymus mongolicus* | Lamiaceae | *Thymus* | Woody | No |
| *Hornungia procumbens* | Brassicaceae | *Hornungia* | Herbaceous | No |
| *Plantago media* | Plantaginaceae | *Plantago* | Herbaceous | No |
| *Erysimum macilentum* | Brassicaceae | *Erysimum* | Herbaceous | No |
| *Carex alajica* | Cyperaceae | *Carex* | Herbaceous | No |
| *Carex duriuscula* | Cyperaceae | *Carex* | Herbaceous | No |
| *Eragrostis cilianensis* | Poaceae | *Eragrostis* | Herbaceous | No |
| *Trollius lilacinus* | Ranunculaceae | *Trollius* | Herbaceous | No |
| *Poa versicolor subsp. stepposa* | Poaceae | *Poa* | Herbaceous | No |
| *Cicuta virosa* | Apiaceae | *Cicuta* | Herbaceous | No |
| *Astragalus brevivexillatus* | Fabaceae | *Astragalus* | Herbaceous | No |
| *Pedicularis kansuensis* | Orobanchaceae | *Pedicularis* | Herbaceous | No |
| *Avena fatua var. glabrata* | Poaceae | *Avena* | Herbaceous | No |
| *Pyrethrum arrasanicum* | Asteraceae | *Pyrethrum* | Herbaceous | No |
| *Elymus glaberrimus* | Poaceae | *Elymus* | Herbaceous | No |
| *Medicago ruthenica* | Fabaceae | *Medicago* | Herbaceous | No |
| *Rhodiola litwinowii* | Crassulaceae | *Rhodiola* | Herbaceous | No |
| *Calligonum jeminaicum* | Polygonaceae | *Calligonum* | Woody | No |
| *Pseudoarabidopsis toxophylla* | Brassicaceae | *Pseudoarabidopsis* | Herbaceous | No |
| *Lappula stricta* | Boraginaceae | *Lappula* | Herbaceous | No |
| *Arthraxon hispidus* | Poaceae | *Arthraxon* | Herbaceous | No |
| *Cynanchum kaschgaricum* | Apocynaceae | *Cynanchum* | Herbaceous | No |
| *Rosa laxa var. kaschgarica* | Rosaceae | *Rosa* | Woody | No |
| *Oxytropis krylovii* | Fabaceae | *Oxytropis* | Herbaceous | No |
| *Calligonum korlaense* | Polygonaceae | *Calligonum* | Woody | No |
| *Utricularia vulgaris* | Lentibulariaceae | *Utricularia* | Herbaceous | No |
| *Lappula duplicicarpa* | Boraginaceae | *Lappula* | Herbaceous | No |
| *Parnassia viridiflora* | Celastraceae | *Parnassia* | Herbaceous | No |
| *Verbena officinalis* | Verbenaceae | *Verbena* | Herbaceous | No |
| *Thymus roseus* | Lamiaceae | *Thymus* | Woody | No |
| *Lomatogonium bellum* | Gentianaceae | *Lomatogonium* | Herbaceous | No |
| *Paraquilegia caespitosa* | Ranunculaceae | *Paraquilegia* | Herbaceous | No |
| *Bupleurum densiflorum* | Apiaceae | *Bupleurum* | Herbaceous | No |
| *Carex pycnostachya* | Cyperaceae | *Carex* | Herbaceous | No |
| *Reaumuria minfengensis* | Tamaricaceae | *Reaumuria* | Woody | No |
| *Oxytropis avisoides* | Fabaceae | *Oxytropis* | Herbaceous | No |
| *Lolium persicum* | Poaceae | *Lolium* | Herbaceous | No |
| *Bromus squarrosus* | Poaceae | *Bromus* | Herbaceous | No |
| *Scutellaria prostrata* | Lamiaceae | *Scutellaria* | Herbaceous | No |
| *Viola mirabilis* | Violaceae | *Viola* | Herbaceous | No |
| *Senecio scandens* | Asteraceae | *Senecio* | Herbaceous | No |
| *Astragalus kukunoricus* | Fabaceae | *Astragalus* | Herbaceous | No |
| *Cotoneaster integerrimus* | Rosaceae | *Cotoneaster* | Woody | No |
| *Stellaria alsine* | Caryophyllaceae | *Stellaria* | Herbaceous | No |
| *Lonicera japonica* | Caprifoliaceae | *Lonicera* | Woody | No |
| *Oxytropis meinshausenii* | Fabaceae | *Oxytropis* | Herbaceous | No |
| *Sibbaldia procumbens* | Rosaceae | *Sibbaldia* | Herbaceous | No |
| *Lappula spinocarpos* | Boraginaceae | *Lappula* | Herbaceous | No |
| *Carex panicea* | Cyperaceae | *Carex* | Herbaceous | No |
| *Geranium sibiricum* | Geraniaceae | *Geranium* | Herbaceous | No |
| *Polygonum molliiforme* | Polygonaceae | *Polygonum* | Herbaceous | No |
| *Delphinium tetragynum* | Ranunculaceae | *Delphinium* | Herbaceous | No |
| *Poa bulbosa subsp. vivipara* | Poaceae | *Poa* | Herbaceous | No |
| *Sorbus tapashana* | Rosaceae | *Sorbus* | Woody | No |
| *Cirsium alberti* | Asteraceae | *Cirsium* | Herbaceous | No |
| *Salix tianschanica* | Salicaceae | *Salix* | Woody | No |
| *Aeluropus micrantherus* | Poaceae | *Aeluropus* | Herbaceous | No |
| *Erigeron krylovii* | Asteraceae | *Erigeron* | Herbaceous | No |
| *Trisetum spicatum subsp. virescens* | Poaceae | *Trisetum* | Herbaceous | No |
| *Iris tenuifolia* | Iridaceae | *Iris* | Herbaceous | No |
| *Artemisia pewzowii* | Asteraceae | *Artemisia* | Herbaceous | No |
| *Seriphidium gracilescens* | Asteraceae | *Seriphidium* | Herbaceous | No |
| *Saussurea simpsoniana* | Asteraceae | *Saussurea* | Herbaceous | No |
| *Picea obovata* | Pinaceae | *Picea* | Woody | No |
| *Oreomecon nudicaulis* | Papaveraceae | *Oreomecon* | Herbaceous | No |
| *Astragalus heterodontus* | Fabaceae | *Astragalus* | Herbaceous | No |
| *Cyperus difformis* | Cyperaceae | *Cyperus* | Herbaceous | No |
| *Convolvulus ammannii* | Convolvulaceae | *Convolvulus* | Herbaceous | No |
| *Taraxacum pseudoatratum* | Asteraceae | *Taraxacum* | Herbaceous | No |
| *Phelipanche coelestis* | Orobanchaceae | *Phelipanche* | Herbaceous | No |
| *Linum corymbulosum* | Linaceae | *Linum* | Herbaceous | No |
| *Stipa bungeana* | Poaceae | *Stipa* | Herbaceous | No |
| *Taraxacum longipyramidatum* | Asteraceae | *Taraxacum* | Herbaceous | No |
| *Amaranthus viridis* | Amaranthaceae | *Amaranthus* | Herbaceous | No |
| *Artemisia argyi* | Asteraceae | *Artemisia* | Herbaceous | No |
| *Patrinia scabiosifolia* | Caprifoliaceae | *Patrinia* | Herbaceous | No |
| *Astragalus scaberrimus* | Fabaceae | *Astragalus* | Herbaceous | No |
| *Astragalus melilotoides* | Fabaceae | *Astragalus* | Herbaceous | No |
| *Jurinea chaetocarpa* | Asteraceae | *Jurinea* | Herbaceous | No |
| *Teloxys aristata* | Amaranthaceae | *Teloxys* | Herbaceous | No |
| *Euphorbia macrorrhiza* | Euphorbiaceae | *Euphorbia* | Herbaceous | No |
| *Thalictrum minus var. hypoleucum* | Ranunculaceae | *Thalictrum* | Herbaceous | No |
| *Symphyotrichum ciliatum* | Asteraceae | *Symphyotrichum* | Herbaceous | No |
| *Crepis multicaulis* | Asteraceae | *Crepis* | Herbaceous | No |
| *Trisetum altaicum* | Poaceae | *Trisetum* | Herbaceous | No |
| *Taraxacum sinicum* | Asteraceae | *Taraxacum* | Herbaceous | No |
| *Youngia japonica* | Asteraceae | *Youngia* | Herbaceous | No |
| *Lagopsis flava* | Lamiaceae | *Lagopsis* | Herbaceous | No |
| *Cancrinia chrysocephala* | Asteraceae | *Cancrinia* | Herbaceous | No |
| *Chondrilla rouillieri* | Asteraceae | *Chondrilla* | Herbaceous | No |
| *Lappula anocarpa* | Boraginaceae | *Lappula* | Herbaceous | No |
| *Thermopsis barbata f. chrysanthus* | Fabaceae | *Thermopsis* | Herbaceous | No |
| *Lonicera spinosa* | Caprifoliaceae | *Lonicera* | Woody | No |
| *Nonea caspica* | Boraginaceae | *Nonea* | Herbaceous | No |
| *Rosa oxyacantha* | Rosaceae | *Rosa* | Woody | No |
| *Veronica oxycarpa* | Plantaginaceae | *Veronica* | Herbaceous | No |
| *Oxytropis tragacanthoides* | Fabaceae | *Oxytropis* | Woody | No |
| *Hypecoum erectum* | Papaveraceae | *Hypecoum* | Herbaceous | No |
| *Cichorium intybus* | Asteraceae | *Cichorium* | Herbaceous | No |
| *Cerastium arvense subsp. strictum* | Caryophyllaceae | *Cerastium* | Herbaceous | No |
| *Typha latifolia* | Typhaceae | *Typha* | Herbaceous | No |
| *Cirsium souliei* | Asteraceae | *Cirsium* | Herbaceous | No |
| *Epilobium hirsutum* | Onagraceae | *Epilobium* | Herbaceous | No |
| *Thermopsis inflata* | Fabaceae | *Thermopsis* | Herbaceous | No |
| *Aster indicus* | Asteraceae | *Aster* | Herbaceous | No |
| *Oxytropis aciphylla* | Fabaceae | *Oxytropis* | Woody | No |
| *Pulsatilla ambigua* | Ranunculaceae | *Pulsatilla* | Herbaceous | No |
| *Stachys byzantina* | Lamiaceae | *Stachys* | Herbaceous | No |
| *Draba winterbottomii* | Brassicaceae | *Draba* | Herbaceous | No |
| *Artemisia eriopoda* | Asteraceae | *Artemisia* | Herbaceous | No |
| *Medicago polymorpha* | Fabaceae | *Medicago* | Herbaceous | No |
| *Anaphalis nepalensis* | Asteraceae | *Anaphalis* | Herbaceous | No |
| *Anchusa italica* | Boraginaceae | *Anchusa* | Herbaceous | No |
| *Rorippa sylvestris* | Brassicaceae | *Rorippa* | Herbaceous | No |
| *Deyeuxia lapponica* | Poaceae | *Deyeuxia* | Herbaceous | No |
| *Ranunculus repens* | Ranunculaceae | *Ranunculus* | Herbaceous | No |
| *Koeleria macrantha* | Poaceae | *Koeleria* | Herbaceous | No |
| *Potentilla strigosa* | Rosaceae | *Potentilla* | Herbaceous | No |
| *Aster ruoqiangensis* | Asteraceae | *Aster* | Herbaceous | No |
| *Rubia deserticola* | Rubiaceae | *Rubia* | Herbaceous | No |
| *Tamarix sachensis* | Tamaricaceae | *Tamarix* | Woody | No |
| *Erigeron oreades* | Asteraceae | *Erigeron* | Herbaceous | No |
| *Silene jeniseensis* | Caryophyllaceae | *Silene* | Herbaceous | No |
| *Corispermum declinatum* | Amaranthaceae | *Corispermum* | Herbaceous | No |
| *Pedicularis achilleifolia* | Orobanchaceae | *Pedicularis* | Herbaceous | No |
| *Centipeda minima* | Asteraceae | *Centipeda* | Herbaceous | No |
| *Ranunculus sceleratus* | Ranunculaceae | *Ranunculus* | Herbaceous | No |
| *Rubus saxatilis* | Rosaceae | *Rubus* | Woody | No |
| *Astragalus suidenensis* | Fabaceae | *Astragalus* | Herbaceous | No |
| *Aegopodium tadshikorum* | Apiaceae | *Aegopodium* | Herbaceous | No |
| *Thalictrum aquilegiifolium var. sibiricum* | Ranunculaceae | *Thalictrum* | Herbaceous | No |
| *Epilobium tianschanicum* | Onagraceae | *Epilobium* | Herbaceous | No |
| *Taraxacum tianschanicum* | Asteraceae | *Taraxacum* | Herbaceous | No |
| *Saussurea lomatolepis* | Asteraceae | *Saussurea* | Herbaceous | No |
| *Delphinium wuqiaense* | Ranunculaceae | *Delphinium* | Herbaceous | No |
| *Askellia karelinii* | Asteraceae | *Askellia* | Herbaceous | No |
| *Arenaria serpyllifolia* | Caryophyllaceae | *Arenaria* | Herbaceous | No |
| *Halogeton glomeratus var. tibeticus* | Amaranthaceae | *Halogeton* | Herbaceous | No |
| *Vickifunkia thomsonii* | Asteraceae | *Vickifunkia* | Herbaceous | No |
| *Melilotus dentatus* | Fabaceae | *Melilotus* | Herbaceous | No |
| *Acantholimon borodinii* | Plumbaginaceae | *Acantholimon* | Woody | No |
| *Draba stenocarpa* | Brassicaceae | *Draba* | Herbaceous | No |
| *Lepidium cordatum* | Brassicaceae | *Lepidium* | Herbaceous | No |
| *Lonicera tatarica* | Caprifoliaceae | *Lonicera* | Woody | No |
| *Salvia deserta* | Lamiaceae | *Salvia* | Herbaceous | No |
| *Potentilla acaulis* | Rosaceae | *Potentilla* | Herbaceous | No |
| *Achillea asiatica* | Asteraceae | *Achillea* | Herbaceous | No |
| *Saussurea elegans* | Asteraceae | *Saussurea* | Herbaceous | No |
| *Iris tectorum* | Iridaceae | *Iris* | Herbaceous | No |
| *Ranunculus nephelogenes var. longicaulis* | Ranunculaceae | *Ranunculus* | Herbaceous | No |
| *Oxytropis stracheyana* | Fabaceae | *Oxytropis* | Herbaceous | No |
| *Sonchus palustris* | Asteraceae | *Sonchus* | Herbaceous | No |
| *Tripogon chinensis* | Poaceae | *Tripogon* | Herbaceous | No |
| *Euphorbia turkestanica* | Euphorbiaceae | *Euphorbia* | Herbaceous | No |
| *Chondrilla ornata* | Asteraceae | *Chondrilla* | Herbaceous | No |
| *Artemisia absinthium* | Asteraceae | *Artemisia* | Herbaceous | No |
| *Pseudopodospermum inconspicuum* | Asteraceae | *Pseudopodospermum* | Herbaceous | No |
| *Doronicum altaicum* | Asteraceae | *Doronicum* | Herbaceous | No |
| *Poa diaphora* | Poaceae | *Poa* | Herbaceous | No |
| *Kengyilia batalinii* | Poaceae | *Kengyilia* | Herbaceous | No |
| *Caragana leucospina* | Fabaceae | *Caragana* | Woody | No |
| *Pulsatilla chinensis* | Ranunculaceae | *Pulsatilla* | Herbaceous | No |
| *Carex duriuscula subsp. rigescens* | Cyperaceae | *Carex* | Herbaceous | No |
| *Cuscuta approximata* | Convolvulaceae | *Cuscuta* | Herbaceous | No |
| *Allium schoenoprasum* | Amaryllidaceae | *Allium* | Herbaceous | No |
| *Haplophyllum dauricum* | Rutaceae | *Haplophyllum* | Herbaceous | No |
| *Scrophularia umbrosa* | Scrophulariaceae | *Scrophularia* | Herbaceous | No |
| *Phlomoides mongolica* | Lamiaceae | *Phlomoides* | Herbaceous | No |
| *Juncus effusus* | Juncaceae | *Juncus* | Herbaceous | No |
| *Hymenidium hedinii* | Apiaceae | *Hymenidium* | Herbaceous | No |
| *Antennaria dioica* | Asteraceae | *Antennaria* | Herbaceous | No |
| *Bupleurum pusillum* | Apiaceae | *Bupleurum* | Herbaceous | No |
| *Heracleum moellendorffii* | Apiaceae | *Heracleum* | Herbaceous | No |
| *Deyeuxia anthoxanthoides* | Poaceae | *Deyeuxia* | Herbaceous | No |
| *Lagotis brevituba* | Plantaginaceae | *Lagotis* | Herbaceous | No |
| *Typha lugdunensis* | Typhaceae | *Typha* | Herbaceous | No |
| *Caragana spinosa* | Fabaceae | *Caragana* | Woody | No |
| *Taraxacum multiscaposum* | Asteraceae | *Taraxacum* | Herbaceous | No |
| *Suaeda kossinskyi* | Amaranthaceae | *Suaeda* | Herbaceous | No |
| *Selaginella nipponica* | Selaginellaceae | *Selaginella* | Herbaceous | No |
| *Gastrocotyle hispida* | Boraginaceae | *Gastrocotyle* | Herbaceous | No |
| *Tamarix austromongolica* | Tamaricaceae | *Tamarix* | Woody | No |
| *Asterothamnus centrali-asiaticus var. procerior* | Asteraceae | *Asterothamnus* | Herbaceous | No |
| *Cystopteris montana* | Cystopteridaceae | *Cystopteris* | Herbaceous | No |
| *Vicia cracca* | Fabaceae | *Vicia* | Herbaceous | No |
| *Lomatogonium gamosepalum* | Gentianaceae | *Lomatogonium* | Herbaceous | No |
| *Taraxacum stanjukoviczii* | Asteraceae | *Taraxacum* | Herbaceous | No |
| *Allium korolkowii* | Amaryllidaceae | *Allium* | Herbaceous | No |
| *Astragalus chinensis* | Fabaceae | *Astragalus* | Herbaceous | No |
| *Morus australis* | Moraceae | *Morus* | Woody | No |
| *Hedysarum kirghisorum* | Fabaceae | *Hedysarum* | Herbaceous | No |
| *Atriplex prostrata* | Amaranthaceae | *Atriplex* | Herbaceous | No |
| *Carex ensifolia* | Cyperaceae | *Carex* | Herbaceous | No |
| *Trollius chinensis* | Ranunculaceae | *Trollius* | Herbaceous | No |
| *Weigela florida* | Caprifoliaceae | *Weigela* | Woody | No |
| *Nepeta cataria* | Lamiaceae | *Nepeta* | Herbaceous | No |
| *Strigosella scorpioides* | Brassicaceae | *Strigosella* | Herbaceous | No |
| *Chondrilla laticoronata* | Asteraceae | *Chondrilla* | Herbaceous | No |
| *Artemisia nanschanica* | Asteraceae | *Artemisia* | Herbaceous | No |
| *Oxytropis lapponica* | Fabaceae | *Oxytropis* | Herbaceous | No |
| *Pennisetum alopecuroides* | Poaceae | *Pennisetum* | Herbaceous | No |
| *Poa nemoralis* | Poaceae | *Poa* | Herbaceous | No |
| *Senecio sylvaticus* | Asteraceae | *Senecio* | Herbaceous | No |
| *Amaranthus hybridus* | Amaranthaceae | *Amaranthus* | Herbaceous | No |
| *Galium tricornutum* | Rubiaceae | *Galium* | Herbaceous | No |
| *Elytrigia repens subsp. longearistata* | Poaceae | *Elytrigia* | Herbaceous | No |
| *Hypochaeris ciliata* | Asteraceae | *Hypochaeris* | Herbaceous | No |
| *Callianthemum pimpinelloides* | Ranunculaceae | *Callianthemum* | Herbaceous | No |
| *Trisetum spicatum subsp. mongolicum* | Poaceae | *Trisetum* | Herbaceous | No |
| *Calligonum densum* | Polygonaceae | *Calligonum* | Woody | No |
| *Nepeta densiflora* | Lamiaceae | *Nepeta* | Herbaceous | No |
| *Potentilla gelida* | Rosaceae | *Potentilla* | Herbaceous | No |
| *Deschampsia cespitosa subsp. pamirica* | Poaceae | *Deschampsia* | Herbaceous | No |
| *Linaria kulabensis* | Plantaginaceae | *Linaria* | Herbaceous | No |
| *Turritis glabra* | Brassicaceae | *Turritis* | Herbaceous | No |
| *Myricaria paniculata* | Tamaricaceae | *Myricaria* | Woody | No |
| *Saccharum ravennae* | Poaceae | *Saccharum* | Herbaceous | No |
| *Barbarea orthoceras* | Brassicaceae | *Barbarea* | Herbaceous | No |
| *Stellaria uda* | Caryophyllaceae | *Stellaria* | Herbaceous | No |
| *Adenophora capillaris* | Campanulaceae | *Adenophora* | Herbaceous | No |
| *Tetracme quadricornis* | Brassicaceae | *Tetracme* | Herbaceous | No |
| *Salix tarbagataica* | Salicaceae | *Salix* | Woody | No |
| *Corydalis tianshanica* | Papaveraceae | *Corydalis* | Herbaceous | No |
| *Hedysarum semenovii* | Fabaceae | *Hedysarum* | Herbaceous | No |
| *Ambrosia artemisiifolia* | Asteraceae | *Ambrosia* | Herbaceous | No |
| *Astragalus dsharkenticus* | Fabaceae | *Astragalus* | Herbaceous | No |
| *Alchemilla sibirica* | Rosaceae | *Alchemilla* | Herbaceous | No |
| *Crucihimalaya tibetica* | Brassicaceae | *Crucihimalaya* | Herbaceous | No |
| *Lappula tenuis* | Boraginaceae | *Lappula* | Herbaceous | No |
| *Allium tenuissimum* | Amaryllidaceae | *Allium* | Herbaceous | No |
| *Galium saurense* | Rubiaceae | *Galium* | Herbaceous | No |
| *Astragalus angustissimus* | Fabaceae | *Astragalus* | Herbaceous | No |
| *Typha orientalis* | Typhaceae | *Typha* | Herbaceous | No |
| *Bupleurum hamiltonii* | Apiaceae | *Bupleurum* | Herbaceous | No |
| *Cancrinia discoidea* | Asteraceae | *Cancrinia* | Herbaceous | No |
| *Betula microphylla* | Betulaceae | *Betula* | Woody | No |
| *Poa parvissima* | Poaceae | *Poa* | Herbaceous | No |
| *Stachyopsis lamiiflora* | Lamiaceae | *Stachyopsis* | Herbaceous | No |
| *Solms-laubachia kashgarica* | Brassicaceae | *Solms-laubachia* | Herbaceous | No |
| *Tanacetum alatavicum* | Asteraceae | *Tanacetum* | Herbaceous | No |
| *Corydalis glaucescens* | Papaveraceae | *Corydalis* | Herbaceous | No |
| *Lamium barbatum* | Lamiaceae | *Lamium* | Herbaceous | No |
| *Stellaria crassifolia* | Caryophyllaceae | *Stellaria* | Herbaceous | No |
| *Cirsium vulgare* | Asteraceae | *Cirsium* | Herbaceous | No |
| *Oxytropis hirta* | Fabaceae | *Oxytropis* | Herbaceous | No |
| *Erigeron seravschanicus* | Asteraceae | *Erigeron* | Herbaceous | No |
| *Alfredia fetissowii* | Asteraceae | *Alfredia* | Herbaceous | No |
| *Stellaria palustris* | Caryophyllaceae | *Stellaria* | Herbaceous | No |
| *Orobanche cernua var. hansii* | Orobanchaceae | *Orobanche* | Herbaceous | No |
| *Ixeris chinensis* | Asteraceae | *Ixeris* | Herbaceous | No |
| *Doronicum turkestanicum* | Asteraceae | *Doronicum* | Herbaceous | No |
| *Sium medium* | Apiaceae | *Sium* | Herbaceous | No |
| *Anaphalis virgata* | Asteraceae | *Anaphalis* | Woody | No |
| *Lithospermum erythrorhizon* | Boraginaceae | *Lithospermum* | Herbaceous | No |
| *Solms-laubachia platycarpa* | Brassicaceae | *Solms-laubachia* | Herbaceous | No |
| *Inula racemosa* | Asteraceae | *Inula* | Herbaceous | No |
| *Leymus arjinshanicus* | Poaceae | *Leymus* | Herbaceous | No |
| *Trollius altaicus* | Ranunculaceae | *Trollius* | Herbaceous | No |
| *Galatella altaica* | Asteraceae | *Galatella* | Herbaceous | No |
| *Calamagrostis altaica* | Poaceae | *Calamagrostis* | Herbaceous | No |
| *Juncus aletaiensis* | Juncaceae | *Juncus* | Herbaceous | No |
| *Seriphidium terrae-albae* | Asteraceae | *Seriphidium* | Herbaceous | No |
| *Lepidium perfoliatum* | Brassicaceae | *Lepidium* | Herbaceous | No |
| *Gentiana olgae* | Gentianaceae | *Gentiana* | Herbaceous | No |
| *Astragalus przhevalskianus* | Fabaceae | *Astragalus* | Herbaceous | No |
| *Alisma gramineum* | Alismataceae | *Alisma* | Herbaceous | No |
| *Carlina biebersteinii* | Asteraceae | *Carlina* | Herbaceous | No |
| *Amaranthus spinosus* | Amaranthaceae | *Amaranthus* | Herbaceous | No |
| *Festuca amblyodes* | Poaceae | *Festuca* | Herbaceous | No |
| *Geranium dahuricum* | Geraniaceae | *Geranium* | Herbaceous | No |
| *Euphorbia wallichii* | Euphorbiaceae | *Euphorbia* | Herbaceous | No |
| *Alisma orientale* | Alismataceae | *Alisma* | Herbaceous | No |
| *Fumaria vaillantii* | Papaveraceae | *Fumaria* | Herbaceous | No |
| *Androsace septentrionalis var. breviscapa* | Primulaceae | *Androsace* | Herbaceous | No |
| *Swertia connata* | Gentianaceae | *Swertia* | Herbaceous | No |
| *Leontopodium brachyactis* | Asteraceae | *Leontopodium* | Herbaceous | No |
| *Gagea fragifera* | Liliaceae | *Gagea* | Herbaceous | No |
| *Stellaria aquatica* | Caryophyllaceae | *Stellaria* | Herbaceous | No |
| *Atraphaxis irtyschensis* | Polygonaceae | *Atraphaxis* | Woody | No |
| *Lagochilus diacanthophyllus* | Lamiaceae | *Lagochilus* | Herbaceous | No |
| *Phedimus aizoon* | Crassulaceae | *Phedimus* | Herbaceous | No |
| *Lomatogonium rotatum* | Gentianaceae | *Lomatogonium* | Herbaceous | No |
| *Hedysarum setosum* | Fabaceae | *Hedysarum* | Herbaceous | No |
| *Saussurea alpina* | Asteraceae | *Saussurea* | Herbaceous | No |
| *Pedicularis elata* | Orobanchaceae | *Pedicularis* | Herbaceous | No |
| *Seriphidium grenardii* | Asteraceae | *Seriphidium* | Herbaceous | No |
| *Ranunculus tanguticus* | Ranunculaceae | *Ranunculus* | Herbaceous | No |
| *Leiospora eriocalyx* | Brassicaceae | *Leiospora* | Herbaceous | No |
| *Ponerorchis chusua* | Orchidaceae | *Ponerorchis* | Herbaceous | No |
| *Eremopyrum triticeum* | Poaceae | *Eremopyrum* | Herbaceous | No |
| *Bupleurum scorzonerifolium* | Apiaceae | *Bupleurum* | Herbaceous | No |
| *Arivela viscosa* | Cleomaceae | *Arivela* | Herbaceous | No |
| *Tragopogon orientalis* | Asteraceae | *Tragopogon* | Herbaceous | No |
| *Polygonum acetosum* | Polygonaceae | *Polygonum* | Herbaceous | No |
| *Arnebia fimbriata* | Boraginaceae | *Arnebia* | Herbaceous | No |
| *Pimpinella anisum* | Apiaceae | *Pimpinella* | Herbaceous | No |
| *Eritrichium thymifolium* | Boraginaceae | *Eritrichium* | Herbaceous | No |
| *Bacopa monnieri* | Plantaginaceae | *Bacopa* | Herbaceous | No |
| *Launaea procumbens* | Asteraceae | *Launaea* | Herbaceous | No |
| *Viola acutifolia* | Violaceae | *Viola* | Herbaceous | No |
| *Valeriana ficariifolia* | Caprifoliaceae | *Valeriana* | Herbaceous | No |
| *Plantago maxima* | Plantaginaceae | *Plantago* | Herbaceous | No |
| *Eritrichium petiolare* | Boraginaceae | *Eritrichium* | Herbaceous | No |
| *Eleocharis valleculosa* | Cyperaceae | *Eleocharis* | Herbaceous | No |
| *Saussurea kaschgarica* | Asteraceae | *Saussurea* | Herbaceous | No |
| *Draba sachalinensis* | Brassicaceae | *Draba* | Herbaceous | No |
| *Seriphidium korovinii* | Asteraceae | *Seriphidium* | Herbaceous | No |
| *Oxytropis ladyginii* | Fabaceae | *Oxytropis* | Herbaceous | No |
| *Semenovia malcolmii* | Apiaceae | *Semenovia* | Herbaceous | No |
| *Polypogon maritimus* | Poaceae | *Polypogon* | Herbaceous | No |
| *Eragrostis japonica* | Poaceae | *Eragrostis* | Herbaceous | No |
| *Elymus viridulus* | Poaceae | *Elymus* | Herbaceous | No |
| *Solidago virgaurea* | Asteraceae | *Solidago* | Herbaceous | No |
| *Portulaca pilosa* | Portulacaceae | *Portulaca* | Herbaceous | No |
| *Oxytropis trichophysa* | Fabaceae | *Oxytropis* | Herbaceous | No |
| *Orobanche ombrochares* | Orobanchaceae | *Orobanche* | Herbaceous | No |
| *Kaschgaria brachanthemoides* | Asteraceae | *Kaschgaria* | Woody | No |
| *Suaeda dendroides* | Amaranthaceae | *Suaeda* | Woody | No |
| *Suaeda australis* | Amaranthaceae | *Suaeda* | Woody | No |
| *Jurinea kaschgarica* | Asteraceae | *Jurinea* | Herbaceous | No |
| *Neotorularia torulosa* | Brassicaceae | *Neotorularia* | Herbaceous | No |
| *Senecio vulgaris* | Asteraceae | *Senecio* | Herbaceous | No |
| *Trigonella pamirica* | Fabaceae | *Trigonella* | Herbaceous | No |
| *Poa araratica subsp. oligophylla* | Poaceae | *Poa* | Herbaceous | No |
| *Euphorbia thymifolia* | Euphorbiaceae | *Euphorbia* | Herbaceous | No |
| *Clematis aethusifolia* | Ranunculaceae | *Clematis* | Herbaceous | No |
| *Artemisia caruifolia* | Asteraceae | *Artemisia* | Herbaceous | No |
| *Asparagus trichophyllus* | Asparagaceae | *Asparagus* | Herbaceous | No |
| *Echinops integrifolius* | Asteraceae | *Echinops* | Herbaceous | No |
| *Astragalus ornithorrhynchus* | Fabaceae | *Astragalus* | Herbaceous | No |
| *Salix sajanensis* | Salicaceae | *Salix* | Woody | No |
| *Tripleurospermum limosum* | Asteraceae | *Tripleurospermum* | Herbaceous | No |
| *Ambrosia trifida* | Asteraceae | *Ambrosia* | Herbaceous | No |
| *Salix nipponica* | Salicaceae | *Salix* | Woody | No |
| *Allium bidentatum* | Amaryllidaceae | *Allium* | Herbaceous | No |
| *Acronychia pedunculata* | Rutaceae | *Acronychia* | Woody | No |
| *Hedysarum neglectum* | Fabaceae | *Hedysarum* | Herbaceous | No |
| *Vickifunkia thyrsoidea* | Asteraceae | *Vickifunkia* | Herbaceous | No |
| *Clematis kirilowii* | Ranunculaceae | *Clematis* | Woody | No |
| *Scorzonera sinensis* | Asteraceae | *Scorzonera* | Herbaceous | No |
| *Asparagus cochinchinensis* | Asparagaceae | *Asparagus* | Herbaceous | No |
| *Carum buriaticum* | Apiaceae | *Carum* | Herbaceous | No |
| *Dicranostigma leptopodum* | Papaveraceae | *Dicranostigma* | Herbaceous | No |
| *Parnassia delavayi* | Celastraceae | *Parnassia* | Herbaceous | No |
| *Ligularia sibirica* | Asteraceae | *Ligularia* | Herbaceous | No |
| *Fritillaria ferganensis* | Liliaceae | *Fritillaria* | Herbaceous | No |
| *Crepis sibirica* | Asteraceae | *Crepis* | Herbaceous | No |
| *Potentilla xizangensis* | Rosaceae | *Potentilla* | Herbaceous | No |
| *Stipa himalaica* | Poaceae | *Stipa* | Herbaceous | No |
| *Caragana stenophylla* | Fabaceae | *Caragana* | Woody | No |
| *Cyperus rotundus* | Cyperaceae | *Cyperus* | Herbaceous | No |
| *Saussurea minuta* | Asteraceae | *Saussurea* | Herbaceous | No |
| *Allium pallasii* | Amaryllidaceae | *Allium* | Herbaceous | No |
| *Cirsium semenowii* | Asteraceae | *Cirsium* | Herbaceous | No |
| *Pedicularis venusta* | Orobanchaceae | *Pedicularis* | Herbaceous | No |
| *Astragalus cobresiiphilus* | Fabaceae | *Astragalus* | Herbaceous | No |
| *Suaeda linifolia* | Amaranthaceae | *Suaeda* | Herbaceous | No |
| *Thymus serpyllum* | Lamiaceae | *Thymus* | Woody | No |
| *Leymus yiunensis* | Poaceae | *Leymus* | Herbaceous | No |
| *Aster alpinus var. diversisquamus* | Asteraceae | *Aster* | Herbaceous | No |
| *Lappula semiglabra var. heterocaryoides* | Boraginaceae | *Lappula* | Herbaceous | No |
| *Salsola praecox* | Amaranthaceae | *Salsola* | Herbaceous | No |
| *Nepeta zandaensis* | Lamiaceae | *Nepeta* | Herbaceous | No |
| *Thalictrum squarrosum* | Ranunculaceae | *Thalictrum* | Herbaceous | No |
| *Hylotelephium spectabile* | Crassulaceae | *Hylotelephium* | Herbaceous | No |
| *Androsace longifolia* | Primulaceae | *Androsace* | Herbaceous | No |
| *Doronicum oblongifolium* | Asteraceae | *Doronicum* | Herbaceous | No |
| *Epilobium palustre* | Onagraceae | *Epilobium* | Herbaceous | No |
| *Tragopogon kasachstanicus* | Asteraceae | *Tragopogon* | Herbaceous | No |
| *taraxacum centrasiaticum* | Asteraceae | *Taraxacum* | Herbaceous | No |
| *Puccinellia minuta* | Poaceae | *Puccinellia* | Herbaceous | No |
| *Hedysarum songaricum* | Fabaceae | *Hedysarum* | Herbaceous | No |
| *Thalictrum isopyroides* | Ranunculaceae | *Thalictrum* | Herbaceous | No |
| *Schoenoplectus subulatus* | Cyperaceae | *Schoenoplectus* | Herbaceous | No |
| *Lappula tianschanica var. altaica* | Boraginaceae | *Lappula* | Herbaceous | No |
| *Linum altaicum* | Linaceae | *Linum* | Herbaceous | No |
| *Lappula alatavica* | Boraginaceae | *Lappula* | Herbaceous | No |
| *Thesium alatavicum* | Santalaceae | *Thesium* | Herbaceous | No |
| *Euphorbia alatavica* | Euphorbiaceae | *Euphorbia* | Herbaceous | No |
| *Lappula macra* | Boraginaceae | *Lappula* | Herbaceous | No |
| *Geranium albiflorum* | Geraniaceae | *Geranium* | Herbaceous | No |
| *Dontostemon senilis* | Brassicaceae | *Dontostemon* | Herbaceous | No |
| *Potentilla fruticosa var. albicans* | Rosaceae | *Potentilla* | Woody | No |
| *Bidens leptophylla* | Asteraceae | *Bidens* | Herbaceous | No |
| *Arabis amplexicaulis* | Brassicaceae | *Arabis* | Herbaceous | No |
| *Pedicularis proboscidea* | Orobanchaceae | *Pedicularis* | Herbaceous | No |
| *Astragalus dschimensis* | Fabaceae | *Astragalus* | Herbaceous | No |
| *Corispermum tibeticum* | Amaranthaceae | *Corispermum* | Herbaceous | No |
| *Taraxacum tibetanum* | Asteraceae | *Taraxacum* | Herbaceous | No |
| *Saussurea elliptica* | Asteraceae | *Saussurea* | Herbaceous | No |
| *Phlomoides umbrosa* | Lamiaceae | *Phlomoides* | Herbaceous | No |
| *Hypericum scabrum* | Hypericaceae | *Hypericum* | Woody | No |
| *Carex liparocarpos* | Cyperaceae | *Carex* | Herbaceous | No |
| *Salsola tamariscina* | Amaranthaceae | *Salsola* | Herbaceous | No |
| *Oreomecon crocea* | Papaveraceae | *Oreomecon* | Herbaceous | No |
| *Pedicularis anthemifolia* | Orobanchaceae | *Pedicularis* | Herbaceous | No |
| *Rosa acicularis* | Rosaceae | *Rosa* | Woody | No |
| *Caragana acanthophylla* | Fabaceae | *Caragana* | Woody | No |
| *Carex caespititia* | Cyperaceae | *Carex* | Herbaceous | No |
| *Glycyrrhiza aspera* | Fabaceae | *Glycyrrhiza* | Herbaceous | No |
| *Galinsoga quadriradiata* | Asteraceae | *Galinsoga* | Herbaceous | No |
| *Astragalus dahuricus* | Fabaceae | *Astragalus* | Herbaceous | No |
| *Cerastium davuricum* | Caryophyllaceae | *Cerastium* | Herbaceous | No |
| *Dianthus hoeltzeri* | Caryophyllaceae | *Dianthus* | Herbaceous | No |
| *Lagochilus macrodontus* | Lamiaceae | *Lagochilus* | Herbaceous | No |
| *Orlaya grandiflora* | Apiaceae | *Orlaya* | Herbaceous | No |
| *Vicia bungei* | Fabaceae | *Vicia* | Herbaceous | No |
| *Euphorbia pekinensis* | Euphorbiaceae | *Euphorbia* | Herbaceous | No |
| *Hordeum secalinum* | Poaceae | *Hordeum* | Herbaceous | No |
| *Saussurea baicalensis* | Asteraceae | *Saussurea* | Herbaceous | No |
| *Leymus ramosus* | Poaceae | *Leymus* | Herbaceous | No |
| *Calligonum leucocladum* | Polygonaceae | *Calligonum* | Woody | No |
| *Rehmannia glutinosa* | Orobanchaceae | *Rehmannia* | Herbaceous | No |
| *Androsace filiformis* | Primulaceae | *Androsace* | Herbaceous | No |
| *Elymus breviaristatus* | Poaceae | *Elymus* | Herbaceous | No |
| *Adonis davidii* | Ranunculaceae | *Adonis* | Herbaceous | No |
| *Trigonotis peduncularis var. amblyosepala* | Boraginaceae | *Trigonotis* | Herbaceous | No |
| *Onobrychis tanaitica* | Fabaceae | *Onobrychis* | Herbaceous | No |
| *Jurinea multiflora* | Asteraceae | *Jurinea* | Herbaceous | No |
| *Gagea ova* | Liliaceae | *Gagea* | Herbaceous | No |
| *Lappula ramulosa* | Boraginaceae | *Lappula* | Herbaceous | No |
| *Anthriscus sylvestris* | Apiaceae | *Anthriscus* | Herbaceous | No |
| *Cynanchum chinense* | Apocynaceae | *Cynanchum* | Herbaceous | No |
| *Arabis auriculata* | Brassicaceae | *Arabis* | Herbaceous | No |
| *Oxytropis bicolor* | Fabaceae | *Oxytropis* | Herbaceous | No |
| *Saposhnikovia divaricata* | Apiaceae | *Saposhnikovia* | Herbaceous | No |
| *Primula farinosa* | Primulaceae | *Primula* | Herbaceous | No |
| *Cephalanthus tetrandrus* | Rubiaceae | *Cephalanthus* | Woody | No |
| *Neottia listeroides* | Orchidaceae | *Neottia* | Herbaceous | No |
| *Phlomoides agraria* | Lamiaceae | *Phlomoides* | Herbaceous | No |
| *Tephroseris kirilowii* | Asteraceae | *Tephroseris* | Herbaceous | No |
| *Cousinia leiocephala* | Asteraceae | *Cousinia* | Herbaceous | No |
| *Taraxacum glabrum* | Asteraceae | *Taraxacum* | Herbaceous | No |
| *Populus pilosa var. leiocarpa* | Salicaceae | *Populus* | Woody | No |
| *Erigeron leioreades* | Asteraceae | *Erigeron* | Herbaceous | No |
| *× Bolboschoenoplectus mariqueter* | Cyperaceae | *× Bolboschoenoplectus* | Herbaceous | No |
| *Taraxacum subglaciale* | Asteraceae | *Taraxacum* | Herbaceous | No |
| *Androsace lehmanniana* | Primulaceae | *Androsace* | Herbaceous | No |
| *Potamogeton gramineus* | Potamogetonaceae | *Potamogeton* | Herbaceous | No |
| *Cynoglossum officinale* | Boraginaceae | *Cynoglossum* | Herbaceous | No |
| *Swertia obtusa* | Gentianaceae | *Swertia* | Herbaceous | No |
| *Agrostis clavata* | Poaceae | *Agrostis* | Herbaceous | No |
| *Crataegus chlorocarpa* | Rosaceae | *Crataegus* | Woody | No |
| *Orobanche pycnostachya* | Orobanchaceae | *Orobanche* | Herbaceous | No |
| *Dendrolobium triangulare* | Fabaceae | *Dendrolobium* | Woody | No |
| *Saussurea acutisquama* | Asteraceae | *Saussurea* | Herbaceous | No |
| *Jacobaea vulgaris* | Asteraceae | *Jacobaea* | Herbaceous | No |
| *Androsace ciliifolia* | Primulaceae | *Androsace* | Herbaceous | No |
| *Androsace bulleyana* | Primulaceae | *Androsace* | Herbaceous | No |
| *Puccinellia florida* | Poaceae | *Puccinellia* | Herbaceous | No |
| *Chorispora greigii* | Brassicaceae | *Chorispora* | Herbaceous | No |
| *Astragalus schanginianus* | Fabaceae | *Astragalus* | Herbaceous | No |
| *Poa urssulensis var. kanboensis* | Poaceae | *Poa* | Herbaceous | No |
| *Puccinellia kashmiriana* | Poaceae | *Puccinellia* | Herbaceous | No |
| *Oxytropis platysema* | Fabaceae | *Oxytropis* | Herbaceous | No |
| *Ranunculus kunlunshanicus* | Ranunculaceae | *Ranunculus* | Herbaceous | No |
| *Erigeron vicarius* | Asteraceae | *Erigeron* | Herbaceous | No |
| *Atriplex dimorphostegia* | Amaranthaceae | *Atriplex* | Herbaceous | No |
| *Geranium sylvaticum* | Geraniaceae | *Geranium* | Herbaceous | No |
| *Linaria vulgaris subsp. chinensis* | Plantaginaceae | *Linaria* | Herbaceous | No |
| *Inula salicina* | Asteraceae | *Inula* | Herbaceous | No |
| *Agrimonia pilosa* | Rosaceae | *Agrimonia* | Herbaceous | No |
| *Gymnaconitum gymnandrum* | Ranunculaceae | *Gymnaconitum* | Herbaceous | No |
| *Halenia elliptica* | Gentianaceae | *Halenia* | Herbaceous | No |
| *Scorzonera luntaiensis* | Asteraceae | *Scorzonera* | Herbaceous | No |
| *Chrysosplenium nudicaule* | Saxifragaceae | *Chrysosplenium* | Herbaceous | No |
| *Spenceria ramalana* | Rosaceae | *Spenceria* | Herbaceous | No |
| *Caragana tibetica* | Fabaceae | *Caragana* | Woody | No |
| *Corispermum tylocarpum* | Amaranthaceae | *Corispermum* | Herbaceous | No |
| *Picris hieracioides* | Asteraceae | *Picris* | Herbaceous | No |
| *Oxytropis trichophora* | Fabaceae | *Oxytropis* | Herbaceous | No |
| *Phyllolobium heydei* | Fabaceae | *Phyllolobium* | Herbaceous | No |
| *Oxytropis bella* | Fabaceae | *Oxytropis* | Herbaceous | No |
| *Poa smirnowii subsp. mariae* | Poaceae | *Poa* | Herbaceous | No |
| *Bassia villosissima* | Amaranthaceae | *Bassia* | Woody | No |
| *Rumex confertus* | Polygonaceae | *Rumex* | Herbaceous | No |
| *Suaeda physophora* | Amaranthaceae | *Suaeda* | Woody | No |
| *Corydalis benecincta* | Papaveraceae | *Corydalis* | Herbaceous | No |
| *Christolea niyaensis* | Brassicaceae | *Christolea* | Herbaceous | No |
| *Isodon ternifolius* | Lamiaceae | *Isodon* | Woody | No |
| *Galinsoga parviflora* | Asteraceae | *Galinsoga* | Herbaceous | No |
| *Thalictrum aquilegiifolium* | Ranunculaceae | *Thalictrum* | Herbaceous | No |
| *Draba alajica* | Brassicaceae | *Draba* | Herbaceous | No |
| *Saussurea salsa var. pamirica* | Asteraceae | *Saussurea* | Herbaceous | No |
| *Juncus inflexus* | Juncaceae | *Juncus* | Herbaceous | No |
| *Dysphania pumilio* | Amaranthaceae | *Dysphania* | Herbaceous | No |
| *Astragalus dilutus* | Fabaceae | *Astragalus* | Herbaceous | No |
| *Koenigia fertilis* | Polygonaceae | *Koenigia* | Herbaceous | No |
| *Eritrichium medicarpum* | Boraginaceae | *Eritrichium* | Herbaceous | No |
| *Phlomoides chinghoensis* | Lamiaceae | *Phlomoides* | Herbaceous | No |
| *Eragrostis autumnalis* | Poaceae | *Eragrostis* | Herbaceous | No |
| *Primula denticulata* | Primulaceae | *Primula* | Herbaceous | No |
| *Baccharoides anthelmintica* | Asteraceae | *Baccharoides* | Herbaceous | No |
| *Eutrema integrifolium* | Brassicaceae | *Eutrema* | Herbaceous | No |
| *Galatella punctata* | Asteraceae | *Galatella* | Herbaceous | No |
| *Leymus ruoqiangensis* | Poaceae | *Leymus* | Herbaceous | No |
| *Stellaria umbellata* | Caryophyllaceae | *Stellaria* | Herbaceous | No |
| *Adenophora stricta* | Campanulaceae | *Adenophora* | Herbaceous | No |
| *Atraphaxis bracteata* | Polygonaceae | *Atraphaxis* | Woody | No |
| *Stellaria arenarioides* | Caryophyllaceae | *Stellaria* | Herbaceous | No |
| *Allium senescens* | Amaryllidaceae | *Allium* | Herbaceous | No |
| *Aster lautureanus* | Asteraceae | *Aster* | Herbaceous | No |
| *Hedysarum alpinum* | Fabaceae | *Hedysarum* | Herbaceous | No |
| *Astragalus hoshanbaoanus* | Fabaceae | *Astragalus* | Herbaceous | No |
| *Saussurea leontodontoides* | Asteraceae | *Saussurea* | Herbaceous | No |
| *Gentianopsis paludosa* | Gentianaceae | *Gentianopsis* | Herbaceous | No |
| *Deyeuxia macilenta* | Poaceae | *Deyeuxia* | Herbaceous | No |
| *Lolium remotum* | Poaceae | *Lolium* | Herbaceous | No |
| *Arnebia szechenyi* | Boraginaceae | *Arnebia* | Herbaceous | No |
| *Prangos didyma* | Apiaceae | *Prangos* | Herbaceous | No |
| *Ligustrum obtusifolium* | Oleaceae | *Ligustrum* | Woody | No |
| *Apluda mutica* | Poaceae | *Apluda* | Herbaceous | No |
| *Tragopogon porrifolius* | Asteraceae | *Tragopogon* | Herbaceous | No |
| *Cyperus iria* | Cyperaceae | *Cyperus* | Herbaceous | No |
| *Pedicularis verticillata subsp. tangutica* | Orobanchaceae | *Pedicularis* | Herbaceous | No |
| *Neottia tianschanica* | Orchidaceae | *Neottia* | Herbaceous | No |
| *Leiospora beketovii* | Brassicaceae | *Leiospora* | Herbaceous | No |
| *Echinops tjanschanicus* | Asteraceae | *Echinops* | Herbaceous | No |
| *Cancrinia tianschanica* | Asteraceae | *Cancrinia* | Herbaceous | No |
| *Lithospermum arvense* | Boraginaceae | *Lithospermum* | Herbaceous | No |
| *Beckmannia syzigachne* | Poaceae | *Beckmannia* | Herbaceous | No |
| *Festuca nitidula* | Poaceae | *Festuca* | Herbaceous | No |
| *Russowia sogdiana* | Asteraceae | *Russowia* | Herbaceous | No |
| *Sabulina litwinowii* | Caryophyllaceae | *Sabulina* | Herbaceous | No |
| *Euphorbia tibetica* | Euphorbiaceae | *Euphorbia* | Herbaceous | No |
| *Ranunculus ficariifolius* | Ranunculaceae | *Ranunculus* | Herbaceous | No |
| *Potentilla sischanensis* | Rosaceae | *Potentilla* | Herbaceous | No |
| *Juncus sikkimensis* | Juncaceae | *Juncus* | Herbaceous | No |
| *Bromus gracillimus* | Poaceae | *Bromus* | Herbaceous | No |
| *Takhtajaniantha pusilla* | Asteraceae | *Takhtajaniantha* | Herbaceous | No |
| *Minuartia helmii* | Caryophyllaceae | *Minuartia* | Herbaceous | No |
| *Silene yetii* | Caryophyllaceae | *Silene* | Herbaceous | No |
| *Persicaria viscosa* | Polygonaceae | *Persicaria* | Herbaceous | No |
| *Crotalaria albida* | Fabaceae | *Crotalaria* | Herbaceous | No |
| *Eritrichium sinomicrocarpum* | Boraginaceae | *Eritrichium* | Herbaceous | No |
| *Anemone rivularis var. flore-minore* | Ranunculaceae | *Anemone* | Herbaceous | No |
| *Ixeridium dentatum* | Asteraceae | *Ixeridium* | Herbaceous | No |
| *Deschampsia cespitosa subsp. orientalis* | Poaceae | *Deschampsia* | Herbaceous | No |
| *Caragana microphylla* | Fabaceae | *Caragana* | Woody | No |
| *Cotoneaster microphyllus* | Rosaceae | *Cotoneaster* | Woody | No |
| *Caragana turkestanica* | Fabaceae | *Caragana* | Woody | No |
| *Poa versicolor subsp. relaxa* | Poaceae | *Poa* | Herbaceous | No |
| *Oxytropis xinglongshanica* | Fabaceae | *Oxytropis* | Herbaceous | No |
| *Fumaria schleicheri* | Papaveraceae | *Fumaria* | Herbaceous | No |
| *Fragaria vesca* | Rosaceae | *Fragaria* | Herbaceous | No |
| *Solidago decurrens* | Asteraceae | *Solidago* | Herbaceous | No |
| *Nepeta transiliensis* | Lamiaceae | *Nepeta* | Herbaceous | No |
| *Carex heterostachya* | Cyperaceae | *Carex* | Herbaceous | No |
| *Thymus marschallianus* | Lamiaceae | *Thymus* | Woody | No |
| *Anemone cathayensis* | Ranunculaceae | *Anemone* | Herbaceous | No |
| *Leontopodium souliei* | Asteraceae | *Leontopodium* | Herbaceous | No |
| *Carex melananthiformis* | Cyperaceae | *Carex* | Herbaceous | No |
| *Barbarea intermedia* | Brassicaceae | *Barbarea* | Herbaceous | No |
| *Brachypodium pinnatum* | Poaceae | *Brachypodium* | Herbaceous | No |
| *Stipa pennata* | Poaceae | *Stipa* | Herbaceous | No |
| *Ribes heterotrichum* | Grossulariaceae | *Ribes* | Woody | No |
| *Saussurea prostrata* | Asteraceae | *Saussurea* | Herbaceous | No |
| *Eritrichium villosum* | Boraginaceae | *Eritrichium* | Herbaceous | No |
| *Stuckenia pamirica* | Potamogetonaceae | *Stuckenia* | Herbaceous | No |
| *Trisetum clarkei* | Poaceae | *Trisetum* | Herbaceous | No |
| *Persicaria longiseta* | Polygonaceae | *Persicaria* | Herbaceous | No |
| *Diplotaxis erucoides* | Brassicaceae | *Diplotaxis* | Herbaceous | No |
| *Nepeta nuda* | Lamiaceae | *Nepeta* | Herbaceous | No |
| *Artemisia stricta* | Asteraceae | *Artemisia* | Herbaceous | No |
| *Cystopteris dickieana* | Cystopteridaceae | *Cystopteris* | Herbaceous | No |
| *Schoenoplectiella wallichii* | Cyperaceae | *Schoenoplectiella* | Herbaceous | No |
| *Commelina diffusa* | Commelinaceae | *Commelina* | Herbaceous | No |
| *Oxytropis songarica* | Fabaceae | *Oxytropis* | Herbaceous | No |
| *Taraxacum lilacinum* | Asteraceae | *Taraxacum* | Herbaceous | No |
| *Asterothamnus alyssoides* | Asteraceae | *Asterothamnus* | Woody | No |
| *Puccinellia arjinshanensis* | Poaceae | *Puccinellia* | Herbaceous | No |
| *Thymus altaicus* | Lamiaceae | *Thymus* | Woody | No |
| *Poa glauca subsp. altaica* | Poaceae | *Poa* | Herbaceous | No |
| *Pseudolysimachion alatavicum* | Plantaginaceae | *Pseudolysimachion* | Herbaceous | No |
| *Ephedra minuta* | Ephedraceae | *Ephedra* | Woody | No |
| *Parnassia mysorensis* | Celastraceae | *Parnassia* | Herbaceous | No |
| *Carex melanostachya* | Cyperaceae | *Carex* | Herbaceous | No |
| *Hylotelephium pallescens* | Crassulaceae | *Hylotelephium* | Herbaceous | No |
| *Scleromitrion diffusum* | Rubiaceae | *Scleromitrion* | Herbaceous | No |
| *Solanum lyratum* | Solanaceae | *Solanum* | Herbaceous | No |
| *Angelica dahurica* | Apiaceae | *Angelica* | Herbaceous | No |
| *Euphorbia maculata* | Euphorbiaceae | *Euphorbia* | Herbaceous | No |
| *Ranunculus similis* | Ranunculaceae | *Ranunculus* | Herbaceous | No |
| *Neottia camtschatea* | Orchidaceae | *Neottia* | Herbaceous | No |
| *Epipactis xanthophaea* | Orchidaceae | *Epipactis* | Herbaceous | No |
| *Linnaea borealis* | Caprifoliaceae | *Linnaea* | Woody | No |
| *Tragopogon pseudomajor* | Asteraceae | *Tragopogon* | Herbaceous | No |
| *Equisetum ramosissimum subsp. debile* | Equisetaceae | *Equisetum* | Herbaceous | No |
| *Scutellaria scordiifolia* | Lamiaceae | *Scutellaria* | Herbaceous | No |
| *Solms-laubachia parryoides* | Brassicaceae | *Solms-laubachia* | Herbaceous | No |
| *Ligularia rumicifolia* | Asteraceae | *Ligularia* | Herbaceous | No |
| *Shivparvatia stracheyi* | Caryophyllaceae | *Shivparvatia* | Herbaceous | No |
| *Astragalus webbianus* | Fabaceae | *Astragalus* | Woody | No |
| *Kengyilia hirsuta* | Poaceae | *Kengyilia* | Herbaceous | No |
| *Centaurea scabiosa subsp. adpressa* | Asteraceae | *Centaurea* | Herbaceous | No |
| *Equisetum pratense* | Equisetaceae | *Equisetum* | Herbaceous | No |
| *Koenigia divaricata* | Polygonaceae | *Koenigia* | Herbaceous | No |
| *Eritrichium nanum* | Boraginaceae | *Eritrichium* | Herbaceous | No |
| *Anthemis cotula* | Asteraceae | *Anthemis* | Herbaceous | No |
| *Catolobus pendulus* | Brassicaceae | *Catolobus* | Herbaceous | No |
| *Erigeron philadelphicus* | Asteraceae | *Erigeron* | Herbaceous | No |
| *Anthriscus nemorosa* | Apiaceae | *Anthriscus* | Herbaceous | No |
| *Ranunculus muricatus* | Ranunculaceae | *Ranunculus* | Herbaceous | No |
| *Bolbitis appendiculata* | Dryopteridaceae | *Bolbitis* | Herbaceous | No |
| *Carex scabrisacca* | Cyperaceae | *Carex* | Herbaceous | No |
| *Carex rugulosa* | Cyperaceae | *Carex* | Herbaceous | No |
| *Cardamine hirsuta* | Brassicaceae | *Cardamine* | Herbaceous | No |
| *Pedicularis rhinanthoides subsp. labellata* | Orobanchaceae | *Pedicularis* | Herbaceous | No |
| *Cynoglossum divaricatum* | Boraginaceae | *Cynoglossum* | Herbaceous | No |
| *Sisymbrium altissimum* | Brassicaceae | *Sisymbrium* | Herbaceous | No |
| *Carex rhynchophysa* | Cyperaceae | *Carex* | Herbaceous | No |
| *Rosa macrophylla* | Rosaceae | *Rosa* | Woody | No |
| *Salvia miltiorrhiza* | Lamiaceae | *Salvia* | Herbaceous | No |
| *Lapsanastrum apogonoides* | Asteraceae | *Lapsanastrum* | Herbaceous | No |
| *Sanguisorba officinalis* | Rosaceae | *Sanguisorba* | Herbaceous | No |
| *Hedysarum limitaneum* | Fabaceae | *Hedysarum* | Herbaceous | No |
| *Zygophyllum cuspidatum* | Zygophyllaceae | *Zygophyllum* | Woody | No |
| *Zornia gibbosa* | Fabaceae | *Zornia* | Herbaceous | No |
| *Corispermum orientale* | Amaranthaceae | *Corispermum* | Herbaceous | No |
| *Sisymbrium orientale* | Brassicaceae | *Sisymbrium* | Herbaceous | No |
| *Capsella orientalis* | Brassicaceae | *Capsella* | Herbaceous | No |
| *Caroxylon orientale* | Amaranthaceae | *Caroxylon* | Woody | No |
| *Oxybasis micrantha* | Amaranthaceae | *Oxybasis* | Herbaceous | No |
| *Lolium temulentum* | Poaceae | *Lolium* | Herbaceous | No |
| *Moneses uniflora* | Ericaceae | *Moneses* | Woody | No |
| *Taraxacum brevirostre* | Asteraceae | *Taraxacum* | Herbaceous | No |
| *Cirsium helenioides* | Asteraceae | *Cirsium* | Herbaceous | No |
| *Euphorbia sororia* | Euphorbiaceae | *Euphorbia* | Herbaceous | No |
| *Stenotaphrum helferi* | Poaceae | *Stenotaphrum* | Herbaceous | No |
| *Puccinellia multiflora* | Poaceae | *Puccinellia* | Herbaceous | No |
| *Ranunculus polyanthemos* | Ranunculaceae | *Ranunculus* | Herbaceous | No |
| *Schizonepeta multifida* | Lamiaceae | *Schizonepeta* | Herbaceous | No |
| *Stachyopsis marrubioides* | Lamiaceae | *Stachyopsis* | Herbaceous | No |
| *Elymus kamoji* | Poaceae | *Elymus* | Herbaceous | No |
| *Jacobaea argunensis* | Asteraceae | *Jacobaea* | Herbaceous | No |
| *Limonium otolepis* | Plumbaginaceae | *Limonium* | Herbaceous | No |
| *Limonium bicolor* | Plumbaginaceae | *Limonium* | Herbaceous | No |
| *Potentilla discolor* | Rosaceae | *Potentilla* | Herbaceous | No |
| *Geranium refractum* | Geraniaceae | *Geranium* | Herbaceous | No |
| *Puccinellia phryganodes* | Poaceae | *Puccinellia* | Herbaceous | No |
| *Euphorbia micractina* | Euphorbiaceae | *Euphorbia* | Herbaceous | No |
| *Zygophyllum kansuense* | Zygophyllaceae | *Zygophyllum* | Herbaceous | No |
| *Rubus lambertianus* | Rosaceae | *Rubus* | Woody | No |
| *Ranunculus acris* | Ranunculaceae | *Ranunculus* | Herbaceous | No |
| *Achillea alpina* | Asteraceae | *Achillea* | Herbaceous | No |
| *Allium obliquum* | Amaryllidaceae | *Allium* | Herbaceous | No |
| *Gentiana siphonantha* | Gentianaceae | *Gentiana* | Herbaceous | No |
| *Hypericum perforatum* | Hypericaceae | *Hypericum* | Herbaceous | No |
| *Dracocephalum argunense* | Lamiaceae | *Dracocephalum* | Herbaceous | No |
| *Woodsia glabella* | Woodsiaceae | *Woodsia* | Herbaceous | No |
| *Rorippa indica* | Brassicaceae | *Rorippa* | Herbaceous | No |
| *Ribes nigrum* | Grossulariaceae | *Ribes* | Woody | No |
| *Bupleurum smithii* | Apiaceae | *Bupleurum* | Herbaceous | No |
| *Festuca tristis* | Poaceae | *Festuca* | Herbaceous | No |
| *Taraxacum erythrospermum* | Asteraceae | *Taraxacum* | Herbaceous | No |
| *Lespedeza bicolor* | Fabaceae | *Lespedeza* | Woody | No |
| *Reynoutria japonica* | Polygonaceae | *Reynoutria* | Herbaceous | No |
| *Indigofera kirilowii* | Fabaceae | *Indigofera* | Woody | No |
| *Aconitum jeholense var. angustius* | Ranunculaceae | *Aconitum* | Herbaceous | No |
| *Astragalus contortuplicatus* | Fabaceae | *Astragalus* | Herbaceous | No |
| *Caragana roborovskyi* | Fabaceae | *Caragana* | Woody | No |
| *Crepidiastrum denticulatum* | Asteraceae | *Crepidiastrum* | Herbaceous | No |
| *Glaucium flavum* | Papaveraceae | *Glaucium* | Herbaceous | No |
| *Potentilla chrysantha* | Rosaceae | *Potentilla* | Herbaceous | No |
| *Corydalis pallida* | Papaveraceae | *Corydalis* | Herbaceous | No |
| *Heteropogon contortus* | Poaceae | *Heteropogon* | Herbaceous | No |
| *Scutellaria baicalensis* | Lamiaceae | *Scutellaria* | Herbaceous | No |
| *Oxytropis cana* | Fabaceae | *Oxytropis* | Herbaceous | No |
| *Erysimum canescens* | Brassicaceae | *Erysimum* | Herbaceous | No |
| *Echinops przewalskyi* | Asteraceae | *Echinops* | Herbaceous | No |
| *Kummerowia striata* | Fabaceae | *Kummerowia* | Herbaceous | No |
| *Saussurea subtriangulata* | Asteraceae | *Saussurea* | Herbaceous | No |
| *Gentiana gyirongensis* | Gentianaceae | *Gentiana* | Herbaceous | No |
| *Taraxacum pingue* | Asteraceae | *Taraxacum* | Herbaceous | No |
| *Crepidiastrum sonchifolium* | Asteraceae | *Crepidiastrum* | Herbaceous | No |
| *Clematis corniculata* | Ranunculaceae | *Clematis* | Woody | No |
| *Lespedeza cuneata* | Fabaceae | *Lespedeza* | Woody | No |
| *Ajuga decumbens* | Lamiaceae | *Ajuga* | Herbaceous | No |
| *Berberis wilsoniae* | Berberidaceae | *Berberis* | Woody | No |
| *Pogonatherum crinitum* | Poaceae | *Pogonatherum* | Herbaceous | No |
| *Rosa sericea* | Rosaceae | *Rosa* | Woody | No |
| *Cynoglossum creticum* | Boraginaceae | *Cynoglossum* | Herbaceous | No |
| *Rubus sachalinensis* | Rosaceae | *Rubus* | Woody | No |
| *Oxytropis platonychia* | Fabaceae | *Oxytropis* | Herbaceous | No |
| *Sanguisorba applanata* | Rosaceae | *Sanguisorba* | Herbaceous | No |
| *Galium boreale var. latifolium* | Rubiaceae | *Galium* | Herbaceous | No |
| *Cirsium leo* | Asteraceae | *Cirsium* | Herbaceous | No |
| *Phyllolobium lasaense* | Fabaceae | *Phyllolobium* | Herbaceous | No |
| *Oxytropis penduliflora* | Fabaceae | *Oxytropis* | Herbaceous | No |
| *Wahlenbergia marginata* | Campanulaceae | *Wahlenbergia* | Herbaceous | No |
| *Leptopyrum fumarioides* | Ranunculaceae | *Leptopyrum* | Herbaceous | No |
| *Leptunis trichodes* | Rubiaceae | *Leptunis* | Herbaceous | No |
| *Scutellaria hypericifolia* | Lamiaceae | *Scutellaria* | Herbaceous | No |
| *Alternanthera sessilis* | Amaranthaceae | *Alternanthera* | Herbaceous | No |
| *Orobanche coerulescens* | Orobanchaceae | *Orobanche* | Herbaceous | No |
| *Gagea filiformis* | Liliaceae | *Gagea* | Herbaceous | No |
| *Cynoglossum furcatum* | Boraginaceae | *Cynoglossum* | Herbaceous | No |
| *Humulus scandens* | Cannabaceae | *Humulus* | Herbaceous | No |
| *Codonopsis foetens subsp. nervosa* | Campanulaceae | *Codonopsis* | Herbaceous | No |
| *Phyllolobium chinense* | Fabaceae | *Phyllolobium* | Herbaceous | No |
| *Miscanthus sinensis* | Poaceae | *Miscanthus* | Herbaceous | No |
| *Oxytropis trichocalycina* | Fabaceae | *Oxytropis* | Herbaceous | No |
| *Puccinellia dolicholepis* | Poaceae | *Puccinellia* | Herbaceous | No |
| *Dracocephalum rupestre* | Lamiaceae | *Dracocephalum* | Herbaceous | No |
| *Epilobium amurense* | Onagraceae | *Epilobium* | Herbaceous | No |
| *Pedicularis dasystachys* | Orobanchaceae | *Pedicularis* | Herbaceous | No |
| *Alternanthera paronychioides* | Amaranthaceae | *Alternanthera* | Herbaceous | No |
| *Astragalus membranaceus var. mongholicus* | Fabaceae | *Astragalus* | Herbaceous | No |
| *Caryopteris mongholica* | Lamiaceae | *Caryopteris* | Woody | No |
| *Cirsium eriophoroides* | Asteraceae | *Cirsium* | Herbaceous | No |
| *Hedysarum minjanense* | Fabaceae | *Hedysarum* | Herbaceous | No |
| *Poa membranigluma* | Poaceae | *Poa* | Herbaceous | No |
| *Matricaria chamomilla* | Asteraceae | *Matricaria* | Herbaceous | No |
| *Caragana korshinskii* | Fabaceae | *Caragana* | Woody | No |
| *Galium bullatum* | Rubiaceae | *Galium* | Herbaceous | No |
| *Rubus hirsutus* | Rosaceae | *Rubus* | Woody | No |
| *Leymus pishanicus* | Poaceae | *Leymus* | Herbaceous | No |
| *Elymus komarovii* | Poaceae | *Elymus* | Herbaceous | No |
| *Lactuca undulata* | Asteraceae | *Lactuca* | Herbaceous | No |
| *Veronica polita* | Plantaginaceae | *Veronica* | Herbaceous | No |
| *Ajania khartensis* | Asteraceae | *Ajania* | Herbaceous | No |
| *Sagina japonica* | Caryophyllaceae | *Sagina* | Herbaceous | No |
| *Artemisia anomala* | Asteraceae | *Artemisia* | Herbaceous | No |
| *Rubia cordifolia* | Rubiaceae | *Rubia* | Herbaceous | No |
| *Eremogone qinghaiensis* | Caryophyllaceae | *Eremogone* | Herbaceous | No |
| *Deyeuxia kokonorica* | Poaceae | *Deyeuxia* | Herbaceous | No |
| *Picea crassifolia* | Pinaceae | *Picea* | Woody | No |
| *Pedicularis integrifolia* | Orobanchaceae | *Pedicularis* | Herbaceous | No |
| *Anthoxanthum odoratum subsp. alpinum* | Poaceae | *Anthoxanthum* | Herbaceous | No |
| *Persicaria kawagoeana* | Polygonaceae | *Persicaria* | Herbaceous | No |
| *Artemisia pubescens* | Asteraceae | *Artemisia* | Herbaceous | No |
| *Leptopetalum strigulosum* | Rubiaceae | *Leptopetalum* | Herbaceous | No |
| *Euphorbia esula* | Euphorbiaceae | *Euphorbia* | Herbaceous | No |
| *Oxytropis saposhnikovii* | Fabaceae | *Oxytropis* | Herbaceous | No |
| *Seriphidium junceum* | Asteraceae | *Seriphidium* | Herbaceous | No |
| *Lappula deserticola* | Boraginaceae | *Lappula* | Herbaceous | No |
| *Tournefortia sibirica* | Boraginaceae | *Tournefortia* | Herbaceous | No |
| *Rosa davurica* | Rosaceae | *Rosa* | Woody | No |
| *Erigeron alpicola* | Asteraceae | *Erigeron* | Herbaceous | No |
| *Erysimum hieraciifolium* | Brassicaceae | *Erysimum* | Herbaceous | No |
| *Synurus deltoides* | Asteraceae | *Synurus* | Herbaceous | No |
| *Corallorhiza trifida* | Orchidaceae | *Corallorhiza* | Herbaceous | No |
| *Solanum americanum* | Solanaceae | *Solanum* | Herbaceous | No |
| *Gentiana oligophylla* | Gentianaceae | *Gentiana* | Herbaceous | No |
| *Oxytropis reniformis* | Fabaceae | *Oxytropis* | Herbaceous | No |
| *Digitaria ciliaris* | Poaceae | *Digitaria* | Herbaceous | No |
| *Lycopodium japonicum* | Lycopodiaceae | *Lycopodium* | Herbaceous | No |
| *Artemisia anethoides* | Asteraceae | *Artemisia* | Herbaceous | No |
| *Gymnadenia conopsea* | Orchidaceae | *Gymnadenia* | Herbaceous | No |
| *Pseudognaphalium affine* | Asteraceae | *Pseudognaphalium* | Herbaceous | No |
| *Sporobolus fertilis* | Poaceae | *Sporobolus* | Herbaceous | No |
| *Elsholtzia myosurus* | Lamiaceae | *Elsholtzia* | Woody | No |
| *Fragariastrum biflorum* | Rosaceae | *Fragariastrum* | Herbaceous | No |
| *Pseudolysimachion linariifolium subsp. dilatatum* | Plantaginaceae | *Pseudolysimachion* | Herbaceous | No |
| *Anemone narcissiflora* | Ranunculaceae | *Anemone* | Herbaceous | No |
| *Ammannia baccifera* | Lythraceae | *Ammannia* | Herbaceous | No |
| *Thalictrum foeniculaceum* | Ranunculaceae | *Thalictrum* | Herbaceous | No |
| *Gentiana tetrasticha* | Gentianaceae | *Gentiana* | Herbaceous | No |
| *Galium bungei* | Rubiaceae | *Galium* | Herbaceous | No |
| *Pedicularis spicata* | Orobanchaceae | *Pedicularis* | Herbaceous | No |
| *Erysimum amurense* | Brassicaceae | *Erysimum* | Herbaceous | No |
| *Euphorbia thomsoniana* | Euphorbiaceae | *Euphorbia* | Herbaceous | No |
| *Crepis tianshanica* | Asteraceae | *Crepis* | Herbaceous | No |
| *Lappula tianschanica* | Boraginaceae | *Lappula* | Herbaceous | No |
| *Adenophora lamarckii* | Campanulaceae | *Adenophora* | Herbaceous | No |
| *Rumex thianschanicus* | Polygonaceae | *Rumex* | Herbaceous | No |
| *Lepidium capitatum* | Brassicaceae | *Lepidium* | Herbaceous | No |
| *Geranium krameri* | Geraniaceae | *Geranium* | Herbaceous | No |
| *Dysphania ambrosioides* | Amaranthaceae | *Dysphania* | Herbaceous | No |
| *Ligularia tulupanica* | Asteraceae | *Ligularia* | Herbaceous | No |
| *Heliotropium ellipticum* | Boraginaceae | *Heliotropium* | Herbaceous | No |
| *Erysimum deflexum* | Brassicaceae | *Erysimum* | Herbaceous | No |
| *Eragrostis curvula* | Poaceae | *Eragrostis* | Herbaceous | No |
| *Gentiana curviphylla* | Gentianaceae | *Gentiana* | Herbaceous | No |
| *Saponaria calabrica* | Caryophyllaceae | *Saponaria* | Herbaceous | No |
| *Anemone weixiensis* | Ranunculaceae | *Anemone* | Herbaceous | No |
| *Anaphalis flaccida* | Asteraceae | *Anaphalis* | Herbaceous | No |
| *Delphinium wentsaii* | Ranunculaceae | *Delphinium* | Herbaceous | No |
| *Veronica peregrina* | Plantaginaceae | *Veronica* | Herbaceous | No |
| *Salix cheilophila* | Salicaceae | *Salix* | Woody | No |
| *Hedysarum flavescens* | Fabaceae | *Hedysarum* | Herbaceous | No |
| *Saussurea ussuriensis* | Asteraceae | *Saussurea* | Herbaceous | No |
| *Silene karekirii* | Caryophyllaceae | *Silene* | Herbaceous | No |
| *Astragalus tecti-mundi* | Fabaceae | *Astragalus* | Herbaceous | No |
| *Astragalus laspurensis* | Fabaceae | *Astragalus* | Herbaceous | No |
| *Cotoneaster zabelii* | Rosaceae | *Cotoneaster* | Woody | No |
| *Berberis sibirica* | Berberidaceae | *Berberis* | Woody | No |
| *Primula tibetica* | Primulaceae | *Primula* | Herbaceous | No |
| *Allardia glabra* | Asteraceae | *Allardia* | Herbaceous | No |
| *Saussurea tibetica* | Asteraceae | *Saussurea* | Herbaceous | No |
| *Gymnadenia orchidis* | Orchidaceae | *Gymnadenia* | Herbaceous | No |
| *Puccinellia roshevitsiana* | Poaceae | *Puccinellia* | Herbaceous | No |
| *Rhodiola himalensis* | Crassulaceae | *Rhodiola* | Herbaceous | No |
| *Oxytropis pusilla* | Fabaceae | *Oxytropis* | Herbaceous | No |
| *Pulsatilla turczaninovii* | Ranunculaceae | *Pulsatilla* | Herbaceous | No |
| *Cyclospermum leptophyllum* | Apiaceae | *Cyclospermum* | Herbaceous | No |
| *Cerastium subpilosum* | Caryophyllaceae | *Cerastium* | Herbaceous | No |
| *Corethrodendron scoparium* | Fabaceae | *Corethrodendron* | Woody | No |
| *Ligularia intermedia* | Asteraceae | *Ligularia* | Herbaceous | No |
| *Argentina stenophylla* | Rosaceae | *Argentina* | Herbaceous | No |
| *Curculigo orchioides* | Hypoxidaceae | *Curculigo* | Herbaceous | No |
| *Tragopogon gracilis* | Asteraceae | *Tragopogon* | Herbaceous | No |
| *Inula linariifolia* | Asteraceae | *Inula* | Herbaceous | No |
| *Sanguisorba tenuifolia var. alba* | Rosaceae | *Sanguisorba* | Herbaceous | No |
| *Vicia hirsuta* | Fabaceae | *Vicia* | Herbaceous | No |
| *Camelina microcarpa* | Brassicaceae | *Camelina* | Herbaceous | No |
| *Epilobium parviflorum* | Onagraceae | *Epilobium* | Herbaceous | No |
| *Erysimum cheiranthoides* | Brassicaceae | *Erysimum* | Herbaceous | No |
| *Deyeuxia neglecta* | Poaceae | *Deyeuxia* | Herbaceous | No |
| *Gentiana parvula* | Gentianaceae | *Gentiana* | Herbaceous | No |
| *Medicago minima* | Fabaceae | *Medicago* | Herbaceous | No |
| *Chrysanthemum oreastrum* | Asteraceae | *Chrysanthemum* | Herbaceous | No |
| *Gentiana olivieri* | Gentianaceae | *Gentiana* | Herbaceous | No |
| *Oxytropis semenovii* | Fabaceae | *Oxytropis* | Herbaceous | No |
| *Rhamphospermum arvense* | Brassicaceae | *Rhamphospermum* | Herbaceous | No |
| *Gagea neopopovii* | Liliaceae | *Gagea* | Herbaceous | No |
| *Elymus sinkiangensis* | Poaceae | *Elymus* | Herbaceous | No |
| *Pyrola xinjiangensis* | Ericaceae | *Pyrola* | Woody | No |
| *Tripleurospermum inodorum* | Asteraceae | *Tripleurospermum* | Herbaceous | No |
| *Scrophularia heucheriiflora* | Scrophulariaceae | *Scrophularia* | Herbaceous | No |
| *Malus sieversii* | Rosaceae | *Malus* | Woody | No |
| *Taraxacum xinyuanicum* | Asteraceae | *Taraxacum* | Herbaceous | No |
| *Scrophularia ningpoensis* | Scrophulariaceae | *Scrophularia* | Herbaceous | No |
| *Cyperus michelianus* | Cyperaceae | *Cyperus* | Herbaceous | No |
| *Eremogone brevipetala* | Caryophyllaceae | *Eremogone* | Herbaceous | No |
| *Urtica fissa* | Urticaceae | *Urtica* | Herbaceous | No |
| *Pedicularis anas* | Orobanchaceae | *Pedicularis* | Herbaceous | No |
| *Glycine tabacina* | Fabaceae | *Glycine* | Herbaceous | No |
| *Limonium franchetii* | Plumbaginaceae | *Limonium* | Herbaceous | No |
| *Rupiphila tachiroei* | Apiaceae | *Rupiphila* | Herbaceous | No |
| *Arrhenatherum elatius* | Poaceae | *Arrhenatherum* | Herbaceous | No |
| *Liparis campylostalix* | Orchidaceae | *Liparis* | Herbaceous | No |
| *Elsholtzia cyprianii* | Lamiaceae | *Elsholtzia* | Herbaceous | No |
| *Arundinella hirta* | Poaceae | *Arundinella* | Herbaceous | No |
| *Oxytropis savellanica* | Fabaceae | *Oxytropis* | Herbaceous | No |
| *Lonicera iliensis* | Caprifoliaceae | *Lonicera* | Woody | No |
| *Libanotis iliensis* | Apiaceae | *Libanotis* | Herbaceous | No |
| *Saussurea canescens* | Asteraceae | *Saussurea* | Herbaceous | No |
| *Seriphidium issykkulense* | Asteraceae | *Seriphidium* | Herbaceous | No |
| *Atriplex micrantha* | Amaranthaceae | *Atriplex* | Herbaceous | No |
| *Erigeron allochrous* | Asteraceae | *Erigeron* | Herbaceous | No |
| *Saussurea brunneopilosa* | Asteraceae | *Saussurea* | Herbaceous | No |
| *Leonurus japonicus* | Lamiaceae | *Leonurus* | Herbaceous | No |
| *Taraxacum alatopetiolum* | Asteraceae | *Taraxacum* | Herbaceous | No |
| *Dasiphora glabra* | Rosaceae | *Dasiphora* | Woody | No |
| *Silene gallica* | Caryophyllaceae | *Silene* | Herbaceous | No |
| *Tridax procumbens* | Asteraceae | *Tridax* | Herbaceous | No |
| *Polygala tenuifolia* | Polygalaceae | *Polygala* | Herbaceous | No |
| *Poa palustris* | Poaceae | *Poa* | Herbaceous | No |
| *Macropodium nivale* | Brassicaceae | *Macropodium* | Herbaceous | No |
| *Clematis hexapetala var. tchefouensis* | Ranunculaceae | *Clematis* | Herbaceous | No |
| *Zygophyllum jaxarticum* | Zygophyllaceae | *Zygophyllum* | Herbaceous | No |
| *Allium saxatile* | Amaryllidaceae | *Allium* | Herbaceous | No |
| *Linaria longicalcarata* | Plantaginaceae | *Linaria* | Herbaceous | No |
| *Stipa kirghisorum* | Poaceae | *Stipa* | Herbaceous | No |
| *Artemisia kuschakewiczii* | Asteraceae | *Artemisia* | Herbaceous | No |
| *Ranunculus rigescens* | Ranunculaceae | *Ranunculus* | Herbaceous | No |
| *Ranunculus cheirophyllus* | Ranunculaceae | *Ranunculus* | Herbaceous | No |
| *Comarum palustre* | Rosaceae | *Comarum* | Herbaceous | No |
| *Androsace erecta* | Primulaceae | *Androsace* | Herbaceous | No |
| *Elymus gmelinii* | Poaceae | *Elymus* | Herbaceous | No |
| *Stellaria chinensis* | Caryophyllaceae | *Stellaria* | Herbaceous | No |
| *Zoysia sinica* | Poaceae | *Zoysia* | Herbaceous | No |
| *Juncus turkestanicus* | Juncaceae | *Juncus* | Herbaceous | No |
| *Euonymus semenovii* | Celastraceae | *Euonymus* | Woody | No |
| *Nepeta virgata* | Lamiaceae | *Nepeta* | Herbaceous | No |
| *Sedum bulbiferum* | Crassulaceae | *Sedum* | Herbaceous | No |
| *Pyrethrum songaricum* | Asteraceae | *Pyrethrum* | Herbaceous | No |
| *Poa lipskyi subsp. dschungarica* | Poaceae | *Poa* | Herbaceous | No |
| *Viola philippica* | Violaceae | *Viola* | Herbaceous | No |
| *Bupleurum commelynoideum* | Apiaceae | *Bupleurum* | Herbaceous | No |
| *Parochetus communis* | Fabaceae | *Parochetus* | Herbaceous | No |
| *Symphyotrichum subulatum* | Asteraceae | *Symphyotrichum* | Herbaceous | No |
| *Calligonum caput-medusae* | Polygonaceae | *Calligonum* | Woody | No |

**Note:** “Included in final modeling dataset” indicates whether each species was retained among the 608 species successfully modeled and included in the subsequent analyses. “Yes” indicates inclusion in the final modeling dataset, whereas “No” indicates that the species was not retained after the sequential data-screening and modeling procedures described in the Materials and Methods.
