# Supplementary material for: Changes in Potential Suitable Habitats, Species Turnover, and Conservation Gaps of Wild Plants in the Tarim Basin, China
Source: Ecol Evol. 2026 Sep 23;16(9):e74393. doi: 10.1002/ece3.74393 (PMC13600769; doi:10.1002/ece3.74393)
Supplement: Supplementary file 2 [file ECE3-16-e74393-s002.docx]

**Supplementary Table S2.** Habitat-loss risk scores and temporal risk shifts of the 608 modeled wild plant species under future climate scenarios

| Habitat-loss risk score (2050s) | Habitat-loss risk score (2070s) | Trend in risk status | Species scientific name |
| --- | --- | --- | --- |
| 2 | 1 | Deterioration | *Juniperus sabina* |
| 2 | 1 | Deterioration | *Festuca rubra* |
| 3 | 2 | Deterioration | *Typha domingensis* |
| 3 | 2 | Deterioration | *Plantago himalaica* |
| 3 | 2 | Deterioration | *Lepidium draba* |
| 3 | 2 | Deterioration | *Suaeda heterophylla* |
| 3 | 2 | Deterioration | *Carex alatauensis* |
| 3 | 2 | Deterioration | *Ligustrum quihoui* |
| 3 | 2 | Deterioration | *Myrtama elegans* |
| 3 | 2 | Deterioration | *Tribulus terrestris* |
| 4 | 2 | Deterioration | *Taraxacum officinale* |
| 3 | 2 | Deterioration | *Carex kokanica* |
| 3 | 2 | Deterioration | *Gagea serotina* |
| 4 | 2 | Deterioration | *Comastoma falcatum* |
| 4 | 2 | Deterioration | *Stuckenia pectinata* |
| 4 | 2 | Deterioration | *Oxytropis humifusa* |
| 3 | 2 | Deterioration | *Glycyrrhiza eglandulosa* |
| 3 | 2 | Deterioration | *Nitraria roborowskii* |
| 4 | 2 | Deterioration | *Echinochloa crus-galli var. mitis* |
| 3 | 2 | Deterioration | *Asparagus angulofractus* |
| 4 | 2 | Deterioration | *Plantago griffithii* |
| 4 | 2 | Deterioration | *Cuscuta australis* |
| 4 | 2 | Deterioration | *Erigeron pseudoseravschanicus* |
| 4 | 2 | Deterioration | *Poa albertii* |
| 3 | 2 | Deterioration | *Carex enervis* |
| 3 | 2 | Deterioration | *Xanthium strumarium* |
| 4 | 2 | Deterioration | *Digitaria violascens* |
| 4 | 2 | Deterioration | *Dasiphora fruticosa* |
| 3 | 2 | Deterioration | *Bolboschoenus planiculmis* |
| 3 | 2 | Deterioration | *Allium przewalskianum* |
| 3 | 2 | Deterioration | *Carex orbicularis* |
| 4 | 2 | Deterioration | *Vicia sativa* |
| 4 | 2 | Deterioration | *Salix pycnostachya* |
| 4 | 2 | Deterioration | *Ajania fastigiata* |
| 4 | 2 | Deterioration | *Bassia scoparia* |
| 4 | 2 | Deterioration | *Halostachys caspica* |
| 3 | 2 | Deterioration | *Suaeda rigida* |
| 4 | 2 | Deterioration | *Polygonum argyrocoleon* |
| 4 | 2 | Deterioration | *Tamarix hohenackeri* |
| 3 | 2 | Deterioration | *Sibbaldia tetrandra* |
| 4 | 2 | Deterioration | *Atriplex sibirica* |
| 4 | 2 | Deterioration | *Elaeagnus angustifolia var. angustifolia* |
| 3 | 2 | Deterioration | *Trichophorum pumilum* |
| 4 | 2 | Deterioration | *Carex atrofusca* |
| 4 | 2 | Deterioration | *Typha laxmannii* |
| 4 | 2 | Deterioration | *Krascheninnikovia ewersmannia* |
| 4 | 2 | Deterioration | *Lepyrodiclis holosteoides* |
| 4 | 2 | Deterioration | *Puccinellia distans* |
| 4 | 2 | Deterioration | *Lagochilus grandiflorus* |
| 4 | 2 | Deterioration | *Aeluropus pungens* |
| 4 | 2 | Deterioration | *Melilotus suaveolens* |
| 4 | 2 | Deterioration | *Poa albertii subsp. kunlunensis* |
| 4 | 2 | Deterioration | *Ribes meyeri* |
| 4 | 2 | Deterioration | *Portulaca oleracea* |
| 4 | 2 | Deterioration | *Digitaria ischaemum* |
| 4 | 2 | Deterioration | *Populus pruinosa* |
| 4 | 2 | Deterioration | *Tamarix elongata* |
| 4 | 2 | Deterioration | *Iljinia regelii* |
| 4 | 2 | Deterioration | *Apocynum pictum* |
| 4 | 2 | Deterioration | *Carex deasyi* |
| 4 | 2 | Deterioration | *Triglochin palustris* |
| 4 | 2 | Deterioration | *Potamogeton pusillus* |
| 4 | 2 | Deterioration | *Calligonum yengisaricum* |
| 4 | 2 | Deterioration | *Equisetum ramosissimum* |
| 4 | 2 | Deterioration | *Scirpus wichurae* |
| 4 | 2 | Deterioration | *Tamarix hispida* |
| 3 | 2 | Deterioration | *Bolboschoenus maritimus* |
| 4 | 2 | Deterioration | *Suaeda prostrata* |
| 4 | 2 | Deterioration | *Chenopodium strictum* |
| 4 | 2 | Deterioration | *Polypogon fugax* |
| 4 | 2 | Deterioration | *Sporobolus aculeatus* |
| 4 | 2 | Deterioration | *Lepidium chalepense* |
| 3 | 2 | Deterioration | *Cirsium japonicum* |
| 4 | 2 | Deterioration | *Lepidium latifolium* |
| 4 | 2 | Deterioration | *Inula caspica* |
| 4 | 2 | Deterioration | *Rorippa palustris* |
| 3 | 2 | Deterioration | *Persicaria orientalis* |
| 4 | 2 | Deterioration | *Echinochloa caudata* |
| 4 | 2 | Deterioration | *Bassia prostrata* |
| 4 | 2 | Deterioration | *Karelinia caspia* |
| 4 | 2 | Deterioration | *Poa annua* |
| 4 | 2 | Deterioration | *Populus euphratica* |
| 4 | 2 | Deterioration | *Takhtajaniantha mongolica* |
| 4 | 2 | Deterioration | *Elaeagnus angustifolia var. orientalis* |
| 4 | 2 | Deterioration | *Salsola collina* |
| 4 | 2 | Deterioration | *Tamarix karelinii* |
| 4 | 2 | Deterioration | *Myricaria pulcherrima* |
| 4 | 2 | Deterioration | *Oxybasis glauca* |
| 4 | 2 | Deterioration | *Salix wilhelmsiana* |
| 4 | 2 | Deterioration | *Halocnemum strobilaceum* |
| 4 | 2 | Deterioration | *Haloxylon ammodendron* |
| 4 | 2 | Deterioration | *Kalidium foliatum* |
| 4 | 2 | Deterioration | *Zygophyllum macropodum* |
| 3 | 2 | Deterioration | *Lythrum salicaria* |
| 4 | 2 | Deterioration | *Typha angustifolia* |
| 4 | 2 | Deterioration | *Calamagrostis macrolepis* |
| 4 | 2 | Deterioration | *Salsola paulsenii* |
| 4 | 2 | Deterioration | *Hippophae rhamnoides subsp. mongolica* |
| 4 | 2 | Deterioration | *Artemisia leucophylla* |
| 4 | 2 | Deterioration | *Persicaria lapathifolia* |
| 4 | 2 | Deterioration | *Convolvulus arvensis* |
| 4 | 2 | Deterioration | *Poa pratensis subsp. angustifolia* |
| 4 | 2 | Deterioration | *Caragana halodendron* |
| 4 | 2 | Deterioration | *Fraxinus sogdiana* |
| 4 | 2 | Deterioration | *Sporobolus schoenoides* |
| 4 | 2 | Deterioration | *Carex melanantha* |
| 4 | 2 | Deterioration | *Sphaerophysa salsula* |
| 4 | 2 | Deterioration | *Solanum villosum* |
| 4 | 2 | Deterioration | *Amaranthus blitum* |
| 4 | 2 | Deterioration | *Lotus tenuis* |
| 4 | 2 | Deterioration | *Phelipanche aegyptiaca* |
| 4 | 2 | Deterioration | *Bolboschoenus yagara* |
| 3 | 2 | Deterioration | *Plantago cornuti* |
| 4 | 2 | Deterioration | *Glycyrrhiza glabra* |
| 4 | 2 | Deterioration | *Echinochloa crus-galli* |
| 4 | 2 | Deterioration | *Polygonum schischkinii* |
| 4 | 2 | Deterioration | *Artemisia desertorum* |
| 4 | 2 | Deterioration | *Medicago lupulina* |
| 4 | 2 | Deterioration | *Elaeagnus angustifolia* |
| 4 | 2 | Deterioration | *Launaea polydichotoma* |
| 4 | 2 | Deterioration | *Sonchus wightianus* |
| 4 | 2 | Deterioration | *Sophora alopecuroides* |
| 4 | 2 | Deterioration | *Abutilon theophrasti* |
| 4 | 2 | Deterioration | *Tamarix chinensis* |
| 4 | 2 | Deterioration | *Cynanchum acutum subsp. sibiricum* |
| 4 | 2 | Deterioration | *Tamarix ramosissima* |
| 4 | 2 | Deterioration | *Phragmites australis* |
| 4 | 2 | Deterioration | *Malva verticillata* |
| 4 | 2 | Deterioration | *Ixeris polycephala* |
| 4 | 2 | Deterioration | *Hibiscus trionum* |
| 4 | 2 | Deterioration | *Glycyrrhiza inflata* |
| 4 | 2 | Deterioration | *Bassia hyssopifolia* |
| 4 | 2 | Deterioration | *Zygophyllum brachypterum* |
| 4 | 2 | Deterioration | *Persicaria maculosa* |
| 4 | 2 | Deterioration | *Solanum nigrum* |
| 4 | 2 | Deterioration | *Salsola tragus* |
| 4 | 2 | Deterioration | *Alhagi camelorum* |
| 4 | 2 | Deterioration | *Lycium ruthenicum* |
| 4 | 2 | Deterioration | *Lotus frondosus* |
| 4 | 3 | Deterioration | *Geranium saxatile* |
| 4 | 3 | Deterioration | *Elymus nutans* |
| 4 | 3 | Deterioration | *Pedicularis dolichorrhiza* |
| 4 | 3 | Deterioration | *Caragana jubata* |
| 4 | 3 | Deterioration | *Chamerion latifolium* |
| 4 | 3 | Deterioration | *Astragalus nivalis* |
| 4 | 3 | Deterioration | *Primula algida* |
| 4 | 3 | Deterioration | *Ligularia alpigena* |
| 4 | 3 | Deterioration | *Polygonum cognatum* |
| 4 | 3 | Deterioration | *Braya rosea* |
| 4 | 3 | Deterioration | *Rhodiola gelida* |
| 4 | 3 | Deterioration | *Christolea crassifolia* |
| 4 | 3 | Deterioration | *Lotus corniculatus* |
| 4 | 3 | Deterioration | *Arnebia euchroma* |
| 4 | 3 | Deterioration | *Festuca kryloviana* |
| 4 | 3 | Deterioration | *Stellaria soongorica* |
| 4 | 3 | Deterioration | *Stipa purpurea* |
| 4 | 3 | Deterioration | *Geranium wilfordii* |
| 4 | 3 | Deterioration | *Lomatogonium carinthiacum* |
| 4 | 3 | Deterioration | *Pedicularis rhinanthoides* |
| 4 | 3 | Deterioration | *Zygophyllum obliquum* |
| 4 | 3 | Deterioration | *Potentilla multifida var. ornithopoda* |
| 4 | 3 | Deterioration | *Calamagrostis pseudophragmites* |
| 4 | 3 | Deterioration | *Dracocephalum moldavica* |
| 4 | 3 | Deterioration | *Bromus japonicus* |
| 4 | 3 | Deterioration | *Salsola pellucida* |
| 4 | 3 | Deterioration | *Datura stramonium* |
| 4 | 3 | Deterioration | *Nitraria pamirica* |
| 4 | 3 | Deterioration | *Plantago major* |
| 4 | 3 | Deterioration | *Cistanche deserticola* |
| 4 | 3 | Deterioration | *Polygonum plebeium* |
| 4 | 3 | Deterioration | *Amaranthus retroflexus* |
| 4 | 3 | Deterioration | *Schoenoplectus tabernaemontani* |
| 4 | 3 | Deterioration | *Nitraria sphaerocarpa* |
| 4 | 3 | Deterioration | *Setaria viridis* |
| 4 | 3 | Deterioration | *Leymus chinensis* |
| 1 | 3 | Improvement | *Stipa caucasica subsp. glareosa* |
| 3 | 4 | Improvement | *Dracocephalum stamineum* |
| 1 | 1 | Stable | *Puccinellia himalaica* |
| 1 | 1 | Stable | *Oxytropis chionobia* |
| 1 | 1 | Stable | *Suaeda corniculata var. olufsenii* |
| 1 | 1 | Stable | *Leymus secalinus var. pubescens* |
| 1 | 1 | Stable | *Saxifraga pulvinaria* |
| 1 | 1 | Stable | *Eritrichium canum* |
| 1 | 1 | Stable | *Rubia tinctorum* |
| 1 | 1 | Stable | *Leiospora exscapa* |
| 1 | 1 | Stable | *Potamogeton crispus* |
| 1 | 1 | Stable | *Allardia tridactylites* |
| 1 | 1 | Stable | *Oxytropis gorbunovii* |
| 1 | 1 | Stable | *Allium carolinianum* |
| 1 | 1 | Stable | *Silene songarica* |
| 1 | 1 | Stable | *Artemisia rupestris* |
| 1 | 1 | Stable | *Juncus bufonius* |
| 1 | 1 | Stable | *Carex pseudofoetida* |
| 1 | 1 | Stable | *Stipa subsessiliflora* |
| 1 | 1 | Stable | *Pedicularis cheilanthifolia* |
| 1 | 1 | Stable | *Androsace maxima* |
| 1 | 1 | Stable | *Astragalus pamirensis* |
| 1 | 1 | Stable | *Delphinium lacostei* |
| 1 | 1 | Stable | *Galium verum* |
| 1 | 1 | Stable | *Cicer microphyllum* |
| 1 | 1 | Stable | *Ephedra fedtschenkoae* |
| 1 | 1 | Stable | *Comastoma pedunculatum* |
| 1 | 1 | Stable | *Oxytropis poncinsii* |
| 1 | 1 | Stable | *Poa calliopsis* |
| 1 | 1 | Stable | *Juncus triglumis* |
| 1 | 1 | Stable | *Puccinellia hackeliana* |
| 1 | 1 | Stable | *Anchusa ovata* |
| 1 | 1 | Stable | *Lappula semiglabra* |
| 1 | 1 | Stable | *Hordeum brevisubulatum subsp. turkestanicum* |
| 1 | 1 | Stable | *Braya scharnhorstii* |
| 1 | 1 | Stable | *Swertia marginata* |
| 1 | 1 | Stable | *Stipa caucasica* |
| 1 | 1 | Stable | *Stipa orientalis* |
| 1 | 1 | Stable | *Leymus secalinus* |
| 1 | 1 | Stable | *Koeleria litvinowii* |
| 1 | 1 | Stable | *Saxifraga hirculus* |
| 1 | 1 | Stable | *Thermopsis turkestanica* |
| 1 | 1 | Stable | *Elymus burchan-buddae* |
| 1 | 1 | Stable | *Sisymbrium brassiciforme* |
| 1 | 1 | Stable | *Blysmus compressus* |
| 2 | 2 | Stable | *Leymus angustus* |
| 2 | 2 | Stable | *Cynodon dactylon* |
| 2 | 2 | Stable | *Digitaria sanguinalis* |
| 2 | 2 | Stable | *Saussurea glacialis* |
| 2 | 2 | Stable | *Primula pamirica* |
| 2 | 2 | Stable | *Poa attenuata* |
| 2 | 2 | Stable | *Allium caricoides* |
| 2 | 2 | Stable | *Echinochloa oryzoides* |
| 2 | 2 | Stable | *Erigeron canadensis* |
| 2 | 2 | Stable | *Acantholimon diapensioides* |
| 2 | 2 | Stable | *Puccinellia hauptiana* |
| 2 | 2 | Stable | *Dracocephalum bipinnatum* |
| 2 | 2 | Stable | *Lepidium appelianum* |
| 2 | 2 | Stable | *Peganum multisectum* |
| 2 | 2 | Stable | *Lagotis decumbens* |
| 2 | 2 | Stable | *Setaria pumila* |
| 2 | 2 | Stable | *Draba oreades* |
| 2 | 2 | Stable | *Echinochloa colona* |
| 2 | 2 | Stable | *Astragalus chomutowii* |
| 2 | 2 | Stable | *Cyperus fuscus* |
| 2 | 2 | Stable | *Elaeagnus oxycarpa* |
| 2 | 2 | Stable | *Stipa breviflora* |
| 2 | 2 | Stable | *Typha minima* |
| 2 | 2 | Stable | *Artemisia mongolica* |
| 4 | 4 | Stable | *Dysphania botrys* |
| 4 | 4 | Stable | *Artemisia dracunculus var. pamirica* |
| 4 | 4 | Stable | *Kengyilia alatavica* |
| 4 | 4 | Stable | *Paropyrum anemonoides* |
| 4 | 4 | Stable | *Carex capillifolia* |
| 4 | 4 | Stable | *Saussurea salsa* |
| 4 | 4 | Stable | *Silene conoidea* |
| 4 | 4 | Stable | *Krascheninnikovia ceratoides* |
| 4 | 4 | Stable | *Hordeum brevisubulatum* |
| 4 | 4 | Stable | *Festuca ovina* |
| 4 | 4 | Stable | *Eutrema altaicum* |
| 4 | 4 | Stable | *Zygophyllum rosowii* |
| 4 | 4 | Stable | *Medicago falcata* |
| 4 | 4 | Stable | *Pedicularis abrotanifolia* |
| 4 | 4 | Stable | *Dasiphora dryadanthoides* |
| 4 | 4 | Stable | *Draba altaica* |
| 4 | 4 | Stable | *Lindelofia stylosa* |
| 4 | 4 | Stable | *Corydalis adiantifolia* |
| 4 | 4 | Stable | *Chorispora sibirica* |
| 4 | 4 | Stable | *Artemisia sieversiana* |
| 4 | 4 | Stable | *Cuscuta europaea* |
| 4 | 4 | Stable | *Lappula squarrosa* |
| 4 | 4 | Stable | *Seriphidium rhodanthum* |
| 4 | 4 | Stable | *Dactylorhiza hatagirea* |
| 4 | 4 | Stable | *Draba nemorosa* |
| 4 | 4 | Stable | *Artemisia annua* |
| 4 | 4 | Stable | *Campanula glomerata* |
| 4 | 4 | Stable | *Allium oreoprasum* |
| 4 | 4 | Stable | *Stipa sareptana var. krylovii* |
| 4 | 4 | Stable | *Lonicera webbiana* |
| 4 | 4 | Stable | *Equisetum arvense* |
| 4 | 4 | Stable | *Crepidiastrum tenuifolium* |
| 4 | 4 | Stable | *Klasea procumbens* |
| 4 | 4 | Stable | *Rumex crispus* |
| 4 | 4 | Stable | *Oxytropis merkensis* |
| 4 | 4 | Stable | *Strigosella hispida* |
| 4 | 4 | Stable | *Halerpestes cymbalaria* |
| 4 | 4 | Stable | *Asterothamnus poliifolius* |
| 4 | 4 | Stable | *Suaeda corniculata* |
| 4 | 4 | Stable | *Thlaspi arvense* |
| 4 | 4 | Stable | *Stipa roborowskyi* |
| 4 | 4 | Stable | *Artemisia dracunculus* |
| 4 | 4 | Stable | *Nitraria schoberi* |
| 4 | 4 | Stable | *Oxytropis coerulea* |
| 4 | 4 | Stable | *Festuca olgae* |
| 4 | 4 | Stable | *Lonicera humilis* |
| 4 | 4 | Stable | *Sonchus brachyotus* |
| 4 | 4 | Stable | *Rudolf-kamelinia korolkowii* |
| 4 | 4 | Stable | *Ephedra regeliana* |
| 4 | 4 | Stable | *Silene gonosperma* |
| 4 | 4 | Stable | *Plantago salsa* |
| 4 | 4 | Stable | *Chorispora tenella* |
| 4 | 4 | Stable | *Rhodiola pamiroalaica* |
| 4 | 4 | Stable | *Rumex patientia* |
| 4 | 4 | Stable | *Bothriochloa ischaemum* |
| 4 | 4 | Stable | *Valeriana fedtschenkoi* |
| 4 | 4 | Stable | *Elymus schrenkianus* |
| 4 | 4 | Stable | *Corispermum lehmannianum* |
| 4 | 4 | Stable | *Geranium pseudosibiricum* |
| 4 | 4 | Stable | *Phlomoides oreophila* |
| 4 | 4 | Stable | *Thermopsis alpina* |
| 4 | 4 | Stable | *Androsace flavescens* |
| 4 | 4 | Stable | *Farinopsis salesoviana* |
| 4 | 4 | Stable | *Lactuca tatarica* |
| 4 | 4 | Stable | *Gueldenstaedtia verna* |
| 4 | 4 | Stable | *Iris lactea* |
| 4 | 4 | Stable | *Astragalus membranaceus* |
| 4 | 4 | Stable | *Leontopodium ochroleucum* |
| 4 | 4 | Stable | *Ranunculus nephelogenes* |
| 4 | 4 | Stable | *Thalictrum foetidum* |
| 4 | 4 | Stable | *Polypogon monspeliensis* |
| 4 | 4 | Stable | *Inula japonica* |
| 4 | 4 | Stable | *Eleocharis quinqueflora* |
| 4 | 4 | Stable | *Juncus articulatus* |
| 4 | 4 | Stable | *Lomatogonium brachyantherum* |
| 4 | 4 | Stable | *Scrophularia incisa* |
| 4 | 4 | Stable | *Clematis florida* |
| 4 | 4 | Stable | *Festuca coelestis* |
| 4 | 4 | Stable | *Aristida adscensionis* |
| 4 | 4 | Stable | *Stephanachne pappophorea* |
| 4 | 4 | Stable | *Chenopodiastrum hybridum* |
| 4 | 4 | Stable | *Hordeum roshevitzii* |
| 4 | 4 | Stable | *Eragrostis pilosa* |
| 4 | 4 | Stable | *Tamarix laxa* |
| 4 | 4 | Stable | *Blitum virgatum* |
| 4 | 4 | Stable | *Geranium collinum* |
| 4 | 4 | Stable | *Youngia heterophylla* |
| 4 | 4 | Stable | *Eragrostis minor* |
| 4 | 4 | Stable | *Deschampsia koelerioides* |
| 4 | 4 | Stable | *Myricaria squamosa* |
| 4 | 4 | Stable | *Thalictrum alpinum* |
| 4 | 4 | Stable | *Takhtajaniantha pseudodivaricata* |
| 4 | 4 | Stable | *Gentianopsis barbata* |
| 4 | 4 | Stable | *Atriplex centralasiatica* |
| 4 | 4 | Stable | *Hippuris vulgaris* |
| 4 | 4 | Stable | *Agriophyllum pungens* |
| 4 | 4 | Stable | *Scrophularia kiriloviana* |
| 4 | 4 | Stable | *Corispermum heptapotamicum* |
| 4 | 4 | Stable | *Rosa fedtschenkoana* |
| 4 | 4 | Stable | *Taraxacum monochlamydeum* |
| 4 | 4 | Stable | *Potentilla multifida* |
| 4 | 4 | Stable | *Pedicularis oederi* |
| 4 | 4 | Stable | *Polygonum aviculare* |
| 4 | 4 | Stable | *Apocynum venetum* |
| 4 | 4 | Stable | *Ajania trilobata* |
| 4 | 4 | Stable | *Taraxacum dealbatum* |
| 4 | 4 | Stable | *Acantholimon hedinii* |
| 4 | 4 | Stable | *Tamarix leptostachya* |
| 4 | 4 | Stable | *Suaeda salsa* |
| 4 | 4 | Stable | *Hippophae rhamnoides* |
| 4 | 4 | Stable | *Ulmus pumila* |
| 4 | 4 | Stable | *Echinops gmelinii* |
| 4 | 4 | Stable | *Ephedra monosperma* |
| 4 | 4 | Stable | *Asparagus breslerianus* |
| 4 | 4 | Stable | *Taraxacum pseudominutilobum* |
| 4 | 4 | Stable | *Ajania fruticulosa* |
| 4 | 4 | Stable | *Rhodiola recticaulis* |
| 4 | 4 | Stable | *Braya humilis* |
| 4 | 4 | Stable | *Caragana polourensis* |
| 4 | 4 | Stable | *Carduus nutans* |
| 4 | 4 | Stable | *Calligonum mongolicum* |
| 4 | 4 | Stable | *Suaeda stellatiflora* |
| 4 | 4 | Stable | *Corydalis stricta* |
| 4 | 4 | Stable | *Chenopodium karoi* |
| 4 | 4 | Stable | *Parnassia oreophila* |
| 4 | 4 | Stable | *Potentilla conferta* |
| 4 | 4 | Stable | *Euphrasia pectinata* |
| 4 | 4 | Stable | *Artemisia demissa* |
| 4 | 4 | Stable | *Caragana sinica* |
| 4 | 4 | Stable | *Agropyron cristatum* |
| 4 | 4 | Stable | *Poa lipskyi* |
| 4 | 4 | Stable | *Senecio thianschanicus* |
| 4 | 4 | Stable | *Chloris virgata* |
| 4 | 4 | Stable | *Acantholimon alatavicum* |
| 4 | 4 | Stable | *Oxybasis rubra* |
| 4 | 4 | Stable | *Ephedra przewalskii* |
| 4 | 4 | Stable | *Artemisia lavandulifolia* |
| 4 | 4 | Stable | *Pulsatilla campanella* |
| 4 | 4 | Stable | *Asterothamnus centraliasiaticus* |
| 4 | 4 | Stable | *Erigeron acris* |
| 4 | 4 | Stable | *Inula britannica* |
| 4 | 4 | Stable | *Zygophyllum fabago* |
| 4 | 4 | Stable | *Viola biflora* |
| 4 | 4 | Stable | *Gymnocarpos przewalskii* |
| 4 | 4 | Stable | *Capsella bursa-pastoris* |
| 4 | 4 | Stable | *Phlomoides pratensis* |
| 4 | 4 | Stable | *Schoenoplectus triqueter* |
| 4 | 4 | Stable | *Oreosalsola laricifolia* |
| 4 | 4 | Stable | *Nitraria sibirica* |
| 4 | 4 | Stable | *Artemisia gmelinii* |
| 4 | 4 | Stable | *Artemisia vulgaris* |
| 4 | 4 | Stable | *Artemisia songarica* |
| 4 | 4 | Stable | *Ephedra glauca* |
| 4 | 4 | Stable | *Clematis songorica var. aspleniifolia* |
| 4 | 4 | Stable | *Salsola sinkiangensis* |
| 4 | 4 | Stable | *Elymus atratus* |
| 4 | 4 | Stable | *Leymus multicaulis* |
| 4 | 4 | Stable | *Carex turkestanica* |
| 4 | 4 | Stable | *Elymus sibiricus* |
| 4 | 4 | Stable | *Lappula brachycentra* |
| 4 | 4 | Stable | *Halerpestes sarmentosa* |
| 4 | 4 | Stable | *Salix alba* |
| 4 | 4 | Stable | *Artemisia scoparia* |
| 4 | 4 | Stable | *Calligonum roborowskii* |
| 4 | 4 | Stable | *Plantago minuta* |
| 4 | 4 | Stable | *Androsace septentrionalis* |
| 4 | 4 | Stable | *Chenopodium iljinii* |
| 4 | 4 | Stable | *Taraxacum scariosum* |
| 4 | 4 | Stable | *Parnassia laxmannii* |
| 4 | 4 | Stable | *Typha davidiana* |
| 4 | 4 | Stable | *Taraxacum dissectum* |
| 4 | 4 | Stable | *Convolvulus fruticosus* |
| 4 | 4 | Stable | *Silene graminifolia* |
| 4 | 4 | Stable | *Gentianella turkestanorum* |
| 4 | 4 | Stable | *Gentiana squarrosa* |
| 4 | 4 | Stable | *Rosa beggeriana* |
| 4 | 4 | Stable | *Orobanche cernua* |
| 4 | 4 | Stable | *Odontites vulgaris* |
| 4 | 4 | Stable | *Hordeum bogdanii* |
| 4 | 4 | Stable | *Potentilla pamiroalaica* |
| 4 | 4 | Stable | *Callianthemum alatavicum* |
| 4 | 4 | Stable | *Salix capusii* |
| 4 | 4 | Stable | *Atriplex tatarica* |
| 4 | 4 | Stable | *Plantago arachnoidea* |
| 4 | 4 | Stable | *Avena fatua* |
| 4 | 4 | Stable | *Rhodiola kashgarica* |
| 4 | 4 | Stable | *Blysmus sinocompressus* |
| 4 | 4 | Stable | *Descurainia sophia* |
| 4 | 4 | Stable | *Taraxacum mongolicum* |
| 4 | 4 | Stable | *Astragalus laxmannii* |
| 4 | 4 | Stable | *Leontopodium fedtschenkoanum* |
| 4 | 4 | Stable | *Grubovia melanoptera* |
| 4 | 4 | Stable | *Cirsium esculentum* |
| 4 | 4 | Stable | *Arnebia guttata* |
| 4 | 4 | Stable | *Astragalus densiflorus* |
| 4 | 4 | Stable | *Rheum reticulatum* |
| 4 | 4 | Stable | *Argentina anserina* |
| 4 | 4 | Stable | *Saussurea ovata* |
| 4 | 4 | Stable | *Euphorbia humifusa* |
| 4 | 4 | Stable | *Leontopodium leontopodioides* |
| 4 | 4 | Stable | *Aster flaccidus* |
| 4 | 4 | Stable | *Kalidium schrenkianum* |
| 4 | 4 | Stable | *Ajania scharnhorstii* |
| 4 | 4 | Stable | *Elymus dahuricus var. cylindricus* |
| 4 | 4 | Stable | *Artemisia macrocephala* |
| 4 | 4 | Stable | *Aster alpinus* |
| 4 | 4 | Stable | *Bidens tripartita* |
| 4 | 4 | Stable | *Chondrilla piptocoma* |
| 4 | 4 | Stable | *Pedicularis violascens* |
| 4 | 4 | Stable | *Seriphidium nitrosum* |
| 4 | 4 | Stable | *Picea schrenkiana* |
| 4 | 4 | Stable | *Agrostis stolonifera* |
| 4 | 4 | Stable | *Ephedra intermedia* |
| 4 | 4 | Stable | *Eriocycla pelliotii* |
| 4 | 4 | Stable | *Sorbus tianschanica* |
| 4 | 4 | Stable | *Gentiana leucomelaena* |
| 4 | 4 | Stable | *Caragana leucophloea* |
| 4 | 4 | Stable | *Orostachys thyrsiflora* |
| 4 | 4 | Stable | *Asperugo procumbens* |
| 4 | 4 | Stable | *Triglochin maritima* |
| 4 | 4 | Stable | *Limonium lacostei* |
| 4 | 4 | Stable | *Artemisia stechmanniana* |
| 4 | 4 | Stable | *Ephedra equisetina* |
| 4 | 4 | Stable | *Juniperus semiglobosa* |
| 4 | 4 | Stable | *Codonopsis clematidea* |
| 4 | 4 | Stable | *Strigosella africana* |
| 4 | 4 | Stable | *Astragalus polycladus* |
| 4 | 4 | Stable | *Leontopodium nanum* |
| 4 | 4 | Stable | *Lepidium ferganense* |
| 4 | 4 | Stable | *Glechoma hederacea* |
| 4 | 4 | Stable | *Rosa webbiana* |
| 4 | 4 | Stable | *Poa litwinowiana* |
| 4 | 4 | Stable | *Tamarix arceuthoides* |
| 4 | 4 | Stable | *Sympegma regelii* |
| 4 | 4 | Stable | *Euphrasia regelii* |
| 4 | 4 | Stable | *Ranunculus popovii* |
| 4 | 4 | Stable | *Salicornia europaea* |
| 4 | 4 | Stable | *Neobrachyactis roylei* |
| 4 | 4 | Stable | *Sibbaldianthe bifurca* |
| 4 | 4 | Stable | *Oxyria digyna* |
| 4 | 4 | Stable | *Astragalus tibetanus* |
| 4 | 4 | Stable | *Pycnoplinthus uniflora* |
| 4 | 4 | Stable | *Leontopodium pusillum* |
| 4 | 4 | Stable | *Clematis songorica* |
| 4 | 4 | Stable | *Aster altaicus* |
| 4 | 4 | Stable | *Androsace ovczinnikovii* |
| 4 | 4 | Stable | *Neopallasia pectinata* |
| 4 | 4 | Stable | *Cirsium sairamense* |
| 4 | 4 | Stable | *Zygophyllum loczyi* |
| 4 | 4 | Stable | *Caragana pleiophylla* |
| 4 | 4 | Stable | *Corispermum mongolicum* |
| 4 | 4 | Stable | *Leymus paboanus* |
| 4 | 4 | Stable | *Schismus arabicus* |
| 4 | 4 | Stable | *Lappula myosotis* |
| 4 | 4 | Stable | *Trollius dschungaricus* |
| 4 | 4 | Stable | *Elsholtzia densa* |
| 4 | 4 | Stable | *Aster semiprostratus* |
| 4 | 4 | Stable | *Richteria pyrethroides* |
| 4 | 4 | Stable | *Androsace squarrosula* |
| 4 | 4 | Stable | *Thalictrum flavum* |
| 4 | 4 | Stable | *Corydalis kashgarica* |
| 4 | 4 | Stable | *Gentiana prostrata var. karelinii* |
| 4 | 4 | Stable | *Berberis kaschgarica* |
| 4 | 4 | Stable | *Arctium lappa* |
| 4 | 4 | Stable | *Glycyrrhiza uralensis* |
| 4 | 4 | Stable | *Pennisetum flaccidum* |
| 4 | 4 | Stable | *Eruca vesicaria subsp. sativa* |
| 4 | 4 | Stable | *Clematis intricata* |
| 4 | 4 | Stable | *Plantago depressa* |
| 4 | 4 | Stable | *Arctopoa tibetica* |
| 4 | 4 | Stable | *Kalidium caspicum* |
| 4 | 4 | Stable | *Saussurea cinerea* |
| 4 | 4 | Stable | *Geranium pratense* |
| 4 | 4 | Stable | *Gentiana pseudoaquatica* |
| 4 | 4 | Stable | *Gentianella azurea* |
| 4 | 4 | Stable | *Limonium aureum* |
| 4 | 4 | Stable | *Lonicera microphylla* |
| 4 | 4 | Stable | *Populus afghanica* |
| 4 | 4 | Stable | *Iris halophila* |
| 4 | 4 | Stable | *Erigeron aurantiacus* |
| 4 | 4 | Stable | *Ligularia macrophylla* |
| 4 | 4 | Stable | *Potentilla chinensis* |
| 4 | 4 | Stable | *Cynomorium songaricum* |
| 4 | 4 | Stable | *Hylotelephium ewersii* |
| 4 | 4 | Stable | *Reaumuria kaschgarica* |
| 4 | 4 | Stable | *Parnassia palustris* |
| 4 | 4 | Stable | *Anabasis aphylla* |
| 4 | 4 | Stable | *Poa pratensis* |
| 4 | 4 | Stable | *Ephedra sinica* |
| 4 | 4 | Stable | *Oxytropis densa* |
| 4 | 4 | Stable | *Acantholimon tianschanicum* |
| 4 | 4 | Stable | *Zygophyllum xanthoxylum* |
| 4 | 4 | Stable | *Chondrilla ambigua* |
| 4 | 4 | Stable | *Capparis spinosa* |
| 4 | 4 | Stable | *Saussurea gnaphalodes* |
| 4 | 4 | Stable | *Viola kunawarensis* |
| 4 | 4 | Stable | *Braya tibetica* |
| 4 | 4 | Stable | *Salsola junatovii* |
| 4 | 4 | Stable | *Suaeda acuminata* |
| 4 | 4 | Stable | *Limonium kaschgaricum* |
| 4 | 4 | Stable | *Juniperus pseudosabina* |
| 4 | 4 | Stable | *Carum carvi* |
| 4 | 4 | Stable | *Rosa platyacantha* |
| 4 | 4 | Stable | *Glaucium squamigerum* |
| 4 | 4 | Stable | *Clematis sibirica* |
| 4 | 4 | Stable | *Dracocephalum imberbe* |
| 4 | 4 | Stable | *Pedicularis physocalyx* |
| 4 | 4 | Stable | *Askellia flexuosa* |
| 4 | 4 | Stable | *Smelowskia tibetica* |
| 4 | 4 | Stable | *Stevenia canescens* |
| 4 | 4 | Stable | *Elymus tangutorum* |
| 4 | 4 | Stable | *Rhodiola coccinea* |
| 4 | 4 | Stable | *Rosularia platyphylla* |
| 4 | 4 | Stable | *Knorringia sibirica subsp. thomsonii* |
| 4 | 4 | Stable | *Convolvulus tragacanthoides* |
| 4 | 4 | Stable | *Lysimachia maritima* |
| 4 | 4 | Stable | *Ajania tibetica* |
| 4 | 4 | Stable | *Saussurea subulata* |
| 4 | 4 | Stable | *Carex microglochin* |
| 4 | 4 | Stable | *Phelipanche caesia* |
| 4 | 4 | Stable | *Taraxacum compactum* |
| 4 | 4 | Stable | *Epilobium minutiflorum* |
| 4 | 4 | Stable | *Artemisia austriaca* |
| 4 | 4 | Stable | *Clematis tangutica* |
| 4 | 4 | Stable | *Dilophia salsa* |
| 4 | 4 | Stable | *Adenophora himalayana* |
| 4 | 4 | Stable | *Berberis nummularia* |
| 4 | 4 | Stable | *Bistorta vivipara* |
| 4 | 4 | Stable | *Potentilla virgata* |
| 4 | 4 | Stable | *Cotoneaster soongoricus* |
| 4 | 4 | Stable | *Knorringia sibirica* |
| 4 | 4 | Stable | *Potentilla sericea* |
| 4 | 4 | Stable | *Saussurea andryaloides* |
| 4 | 4 | Stable | *Ligusticum mucronatum* |
| 4 | 4 | Stable | *Leymus ovatus* |
| 4 | 4 | Stable | *Saussurea thomsonii* |
| 4 | 4 | Stable | *Reaumuria songarica* |
| 4 | 4 | Stable | *Gentianopsis stricta* |
| 4 | 4 | Stable | *Krascheninnikovia compacta* |
| 4 | 4 | Stable | *Agrostis gigantea* |
| 4 | 4 | Stable | *Rosa albertii* |
| 4 | 4 | Stable | *Iris loczyi* |
| 4 | 4 | Stable | *Taraxacum luridum* |
| 4 | 4 | Stable | *Artemisia frigida* |
| 4 | 4 | Stable | *Thalictrum minus* |
| 4 | 4 | Stable | *Berberis amurensis* |
| 4 | 4 | Stable | *Caragana turfanensis* |
| 4 | 4 | Stable | *Carex moorcroftii* |
| 4 | 4 | Stable | *Oxytropis falcata* |
| 4 | 4 | Stable | *Dracocephalum heterophyllum* |
| 4 | 4 | Stable | *Elytrigia repens* |
| 4 | 4 | Stable | *Artemisia rutifolia* |
| 4 | 4 | Stable | *Epipactis helleborine* |
| 4 | 4 | Stable | *Oreosalsola abrotanoides* |
| 4 | 4 | Stable | *Saussurea lacostei* |
| 4 | 4 | Stable | *Taraxacum albiflos* |
| 4 | 4 | Stable | *Dilophia ebracteata* |
| 4 | 4 | Stable | *Asterothamnus fruticosus* |
| 4 | 4 | Stable | *Caragana dasyphylla* |
| 4 | 4 | Stable | *Crepis rigescens* |
| 4 | 4 | Stable | *Primula nutans* |
| 4 | 4 | Stable | *Saussurea thoroldii* |
| 4 | 4 | Stable | *Berberis heteropoda* |
| 4 | 4 | Stable | *Aster hispidus* |

**Note:** Habitat-loss risk scores were assigned according to the number of future climate scenarios in which a species experienced severe habitat loss (LSH ≥ 80%). Scores of 1, 2, 3, and 4 indicate that the severe-loss threshold was reached under three, two, one, and zero SSP scenarios, respectively; therefore, lower scores indicate higher habitat-loss risk. “Trend in risk status” describes the change in risk score from the 2050s to the 2070s: deterioration indicates a decrease in score, stable indicates no change, and improvement indicates an increase in score.
